# *Diaporthe* Species on Palms: Molecular Re-Assessment and Species Boundaries Delimitation in the *D. arecae* Species Complex

**DOI:** 10.3390/microorganisms11112717

**Published:** 2023-11-06

**Authors:** Diana S. Pereira, Sandra Hilário, Micael F. M. Gonçalves, Alan J. L. Phillips

**Affiliations:** 1 Faculdade de Ciências, Biosystems and Integrative Sciences Institute (BioISI), Universidade de Lisboa, Campo Grande, 1749-016 Lisboa, Portugal; santospereira.dsp@gmail.com; 2 Interdisciplinary Centre of Marine and Environmental Research (CIIMAR), Terminal de Cruzeiros do Porto de Leixões, Av. General Norton de Matos s/n, 4450-208 Porto, Portugal; hilario.sandra@fc.up.pt; 3 Faculty of Sciences, Biology Department, University of Porto, Rua do Campo Alegre, Edifício FC4, 4169-007 Porto, Portugal; 4 Centre for Environmental and Marine Studies, Department of Biology, Campus Universitário de Santiago, University of Aveiro, 3810-193 Aveiro, Portugal

**Keywords:** coalescent models, GCPSR, leaf diseases, palm fungi, species boundaries, taxonomy

## Abstract

Due to cryptic diversification, phenotypic plasticity and host associations, multilocus phylogenetic analyses have become the most important tool in accurately identifying and circumscribing species in the *Diaporthe* genus. However, the application of the genealogical concordance criterion has often been overlooked, ultimately leading to an exponential increase in novel *Diaporthe* spp. Due to the large number of species, many lineages remain poorly understood under the so-called species complexes. For this reason, a robust delimitation of the species boundaries in *Diaporthe* is still an ongoing challenge. Therefore, the present study aimed to resolve the species boundaries of the *Diaporthe arecae* species complex (DASC) by implementing an integrative taxonomic approach. The Genealogical Phylogenetic Species Recognition (GCPSR) principle revealed incongruences between the individual gene genealogies. Moreover, the Poisson Tree Processes’ (PTPs) coalescent-based species delimitation models identified three well-delimited subclades represented by the species *D. arecae*, *D. chiangmaiensis* and *D. smilacicola*. These results evidence that all species previously described in the *D. arecae* subclade are conspecific, which is coherent with the morphological indistinctiveness observed and the absence of reproductive isolation and barriers to gene flow. Thus, 52 *Diaporthe* spp. are reduced to synonymy under *D. arecae*. Recent population expansion and the possibility of incomplete lineage sorting suggested that the *D. arecae* subclade may be considered as ongoing evolving lineages under active divergence and speciation. Hence, the genetic diversity and intraspecific variability of *D. arecae* in the context of current global climate change and the role of *D. arecae* as a pathogen on palm trees and other hosts are also discussed. This study illustrates that species in *Diaporthe* are highly overestimated, and highlights the relevance of applying an integrative taxonomic approach to accurately circumscribe the species boundaries in the genus *Diaporthe*.

## 1. Introduction

*Diaporthe* (syn. *Phomopsis*) species are well known as pathogens, endophytes and saprobes in economically important crops, ornamentals and forest trees, but also occur as pathogens in humans and other mammals [1,2,3,4,5]. Along with its diverse host ranges and cosmopolitan distribution, the interest in this genus has increased over the decades due to its recurrent association with plant diseases [4,6,7,8,9,10,11,12,13]. Several studies have reported that *Diaporthe* spp. cause diverse suites of diseases, including leaf spots, blights, root and fruit rots, seed decay, stem cankers, dieback and wilting [14,15,16,17,18,19,20,21]. Given that the implementation of international phytosanitary measures relies on the correct identification of the phytopathogenic fungi [22], the taxonomy of *Diaporthe* has often been re-evaluated to construct a reliable and natural framework for species identification [13,23,24,25,26,27].

Species identification in the genus *Diaporthe* was formerly based on morphological characters and host association [4,5,6,24], leading to a proliferation of more than 2000 species names [28]. However, due to phenotypic plasticity, morphological characters and host association proved to be inadequate for species identification in the genus [4,14,29,30]. Currently, the circumscription of species in *Diaporthe* relies mostly on multi-gene phylogenies based on the nuclear ribosomal internal transcribed spacer region (ITS) and partial sequences of the translation elongation factor 1-α (*tef1*), β-tubulin (*tub2*), histone H3 (*his3*), and calmodulin (*cal*) genes [5,9,23,24,26,31,32].

Molecular studies have greatly clarified the taxonomy of the genus *Diaporthe*, for instance, by unveiling its paraphyletic nature [26,33]. However, defining species boundaries remains a major challenge in *Diaporthe*. Researchers have often found difficulties in interpreting their phylogenetic analyses, which may be related either to limited sampling in many clades, or the use of DNA barcodes with insufficient phylogenetic resolution [34]. As a consequence, many studies of the genus have grouped some species into species complexes, such as *D. amygdali*, *D. arecae*, *D. eres* and *D. sojae*, thus assisting in an accurate identification of taxa [7,9,27,35,36,37]. Recently, Norphanphoun et al. [27] formalized the concept of species complexes in *Diaporthe* based on an inferred phylogenetic analysis of a comprehensive dataset of the five most common loci used to identify species in *Diaporthe*. While several efforts have been made over the last years to resolve the species boundaries of some of those complexes, the accurate identification of species within the *D. arecae* species complex (DASC) has been overlooked.

*Diaporthe arecae* was introduced by Srivastava et al. [38] as *Subramanella arecae* associated with a severe post-harvest fruit rot of *Areca catechu* in India. The species was later assigned to *Diaporthe* based on an ex-isotype culture by Gomes et al. [24]. However, these authors revealed that most loci used to infer the phylogenetic relationships in *Diaporthe* failed to resolve the phylogenetic position of *D. arecae* and its related species. Later, based on morpho-molecular analyses, Tan et al. [39] introduced three new closely related species to *D. arecae*, but they showed low bootstrap support values. The problematic clade was first designated as the *D. arecae* species complex by Huang et al. [35], who isolated 13 endophytic strains from *Citrus* spp. in different provinces of China that were clustered in a poorly supported clade with the ex-isotype strain of *D. arecae*. Huang and co-workers were the first to recognize that the species boundaries within the DASC should be carefully re-evaluated, so they “refrained from defining novel taxa within the complex” [35]. Although a few authors have followed the same strategy [40], over the years more than 40 species, including important phytopathogens, distributed worldwide, have been introduced in the DASC. For instance, Guarnaccia and Crous [10] introduced *D. limonicola* and *D. melitensis* in the DASC as a devasting dieback disease affecting *Citrus* in Europe. Contrarily, minor pathogens, such as *D. pescicola* and *D. taoicola* [41] and *D. guangxiensis* and *D. viniferae* [21], were introduced in the same species complex associated with dieback symptoms in *Prunus persica* and *Vitis vinifera* in China, respectively. Moreover, *D. oculi* and *D. pseudooculi* were introduced to the DASC by Ozawa et al. [42] as human pathogens causing eye diseases. This evidence suggests that the ecology of the DASC is complex and may include, besides phytopathogens, some species involved in human invasive infections.

It has long been recommended that new *Diaporthe* species should be carefully introduced [26,32,43]. However, most species belonging to the DASC were introduced based on the concatenation of sequences from different loci, disregarding the application of the Genealogical Concordance Phylogenetic Species Recognition (GCPSR) principle [44]. This common practice often misleads tree species estimation and tends to overestimate the true species diversity, since each clade in combined-gene genealogies is often recognized as a distinct lineage [45,46,47,48]. The GCPSR principle was proposed by Taylor et al. [44] based on the Genealogical Concordance Species Concept (GCSC) by Avise and Ball [49]. The GCPSR assumes that recombination within a lineage creates conflict between individual gene genealogies; thus, the phylogenetic concordance represented by the transition from conflict to congruence detects the lack of gene flow and defines the limit of a species [44]. Nonetheless, delimiting species boundaries in closely related taxa using multilocus phylogenies is not always straightforward. Genes can exhibit different evolutionary histories, which result in conflicts between individual gene genealogies and ultimately mislead the relationships among closely related taxa [46,47,50,51,52]. These conflicts may arise not only from recombination events, but also from incomplete lineage sorting (ILS), in which some alleles are not expected to be reciprocally monophyletic in the initial stages of speciation [34,51,53,54].

For the above reasons, complementary methods, such as haplotype networks, splits graphs (phylogenetic networks), population genetic diversity analyses and coalescent-based species delimitation methods, have recently been proposed to determine species boundaries in *Diaporthe* more accurately [36,37,55]. As an alternative to the GCPSR criteria, the coalescent methods, based on the Multispecies Coalescent (MSC) model [56], provide a framework for phylogenetic inference based on ancestral polymorphisms and the so-called gene-tree/species-tree conflict [51,54,57,58,59]. Such methods provide a more comprehensive view of speciation events, since they can infer species trees and estimate species boundaries even when there is incongruence between individual gene genealogies and a lack of reciprocal monophyly among lineages [57,60,61]. Despite the utility of coalescent-based methods to support species delimitation, they have rarely been used in phytopathogenic fungi, namely *Alternaria* [47], *Beauveria* [62], *Colletotrichum* [34], *Fusarium* [48], genera of lichenized fungi [63] and, more recently, *Diaporthe* [36,37]. Hilário et al. [37] have resolved the *D. amygdali* species complex, providing evidence that it constitutes a single species through the application of the GCPSR principle, along with coalescent-based models. Likewise, the same methodology has been applied to successfully resolve the *D. eres* species complex [36], which has been shown to constitute a population with intraspecific variability rather than different lineages.

During a survey leaf spotting fungi associated with palm trees in Lisbon, Portugal, several *Diaporthe* taxa have been isolated and preliminary results have been reported in [64]. The purpose of the present study is to: (1) re-assess the morphological and molecular characterization of the isolates obtained from foliar lesions of palms that belong to the DASC; and (2) resolve the species boundaries of the DASC by implementing an integrative taxonomic approach comprising single and multilocus phylogenetic analyses, coalescent-based species delimitation methods, phylogenetic networks, hierarchical cluster analysis of phenotypic data and assessment of recombination and population genetic diversity.

## 2. Material and Methods

### 2.1. Specimen Collection, Examination, and Single-Spore Isolation

In 2018, diseased leaf segments and leaflets with foliar lesions were collected from ornamental palm trees in Lisbon, Portugal. Plant material was transported to the laboratory in paper envelopes and examined with a Leica MZ9.5 stereo microscope (Leica Microsystems GmbH, Wetzlar, Germany) for observation of lesion morphology and associated fungi. Isolations were made directly from foliar lesions following the methods described by Pereira and Phillips [65].

The isolates used in the present study, CDP 0047, CDP 0358 (*D. pseudophoenicicola*) and CDP 0460 (*D. chamaeropicola*), belong to the DASC and were previously reported in a preliminary study on *Diaporthe* occurring on palms published in [64]. Their morphological observation and characterization were re-accessed here.

### 2.2. Morphological Observation and Characterization

Cultures were induced to sporulate by culturing on 2% water Agar (WA) (Bacteriological Agar Type E; BIOKAR Diagnostics, Allonne, France) bearing healthy double-autoclaved palm leaf pieces. After incubating at 28 °C under a 12 h near-ultraviolet light/12 h dark cycle, from 3 days to 1 week, conidiomata were cut through vertically, and the conidiogenous layer was dissected. Microscopic structures (pycnidia, conidiophores, conidiogenous cells and conidia) were mounted in 100% lactic acid and examined by differential interference contrast (DIC) microscopy. Observations on micromorphological features were made using Leica MZ9.5 and Leica DMR microscopes (Leica Microsystems GmbH, Wetzlar, Germany), and digital images were recorded with Leica DFC300 and Leica DFC320 cameras (Leica Microsystems GmbH, Wetzlar, Germany), respectively. Measurements were made with the measurement module of the Leica IM500 Image Management System (Leica Microsystems GmbH, Wetzlar, Germany). Mean, standard deviation (SD) and 95% confidence intervals were calculated from n = total of measured structures. Measurements are given as minimum and maximum dimensions with mean and SD in parenthesis. Photoplates were prepared with Adobe Photoshop CS6 Extended (Adobe, San Jose, CA, USA).

### 2.3. Sequence Alignment and Phylogenetic Analyses

A preliminary identification, based on BLASTn searches with the ITS sequences of the isolates from the present study, was carried out to determine the most closely related taxa, whose sequences were subsequently retrieved from GenBank. Species of *Diaporthe* isolated from palm tissues listed in the recent literature [26,66,67] or deposited in GenBank were also used. A total of 127 strains currently accepted in the genus *Diaporthe* were used to perform an initial phylogenetic analysis based on the ITS sequences. The ingroup taxa included three isolates from this study (CDP 0047, CDP 0358 and CDP 460), 22 strains isolated from palm tissues retrieved from recent literature or from GenBank (BR74, HNHK01, HNHK02, HNHK03, HNQH02, HNQH03, HNQZ01, HNWC01, HNWC02, HNWN03, LC 6150, LC 6151, SM28, SM29, SM30, SM35, SM36, SM38, SM39, SM41, SM45 and SM49) and 94 strains of related *Diaporthe* species retrieved from GenBank (Table 1). This analysis was conducted to select the species recognized within the DASC. The resulting tree was compared with the recent literature on *Diaporthe* and a highly supported clade representing the DASC was selected for further analyses.

Sequences for each locus were aligned with ClustalX version 2.1 [68] using the following parameters: pairwise alignment parameters (gap opening = 10, gap extension = 0.1) and multiple alignment parameters (gap opening = 10, gap extension = 0.2, DNA transition weight = 0.5, delay divergent sequences = 25%). Alignments were checked, and manual adjustments were made wherever necessary with BioEdit version 7.0.5.3 [69]. Terminal regions with missing data and ambiguously aligned regions were excluded from the analysis. Sequences were combined in concatenated matrices using MEGA X version 10.2.6 [70]. Partition homogeneity was assessed using the incongruence length difference (ILD) test [71] performed in PAUP version 4.0a165 [72] to determine the congruency of genes and whether they could be combined.

Maximum likelihood (ML), maximum parsimony (MP) and Bayesian analyses (BA) were used for phylogenetic inferences of the single gene and concatenated alignments and were implemented on the CIPRES Science Gateway portal version 3.3 [73] using RAxML-NG version 1.1.0 [74], PAUP version 4.0a165 [72] and MrBayes version 3.2.7a [75], respectively. The resulting trees were visualized with FigTree version 1.4.4 [76] and prepared with Adobe Illustrator CS2 version 12.0.0 (Adobe, San Jose, CA, USA).

For ML and BA inferences, the best-fit nucleotide substitution model for each locus was determined using MEGA X version 10.2.6 [70] under the Akaike information criterion (AIC), except for the primary phylogenetic analyses of the concatenated alignment containing all species in the DASC. In this case, ML and BA inferences were performed using a general time reversible (GTR) nucleotide substitution model including a discrete gamma distribution and estimation of proportion of invariable sites (GTR + G + I) to accommodate variable rates across sites. Clade stability and robustness of the branches of the best scoring ML tree were estimated by conducting a rapid bootstrap (BS) analysis with iterations halted automatically by RAxML.

MP were performed using the heuristic search option with 1000 random taxa additions and tree bisection and reconnection (TBR) as the branch-swapping algorithm. All characters were unordered and of equal weight, and alignment gaps were treated as missing data. Maxtrees were set to 10,000, branches of zero length were collapsed and all multiple, and equally parsimonious, trees were saved. Clade stability and robustness of the most parsimonious trees were assessed using BS analysis with 1000 pseudoreplicates each with 10 replicates of random stepwise addition of taxa. Descriptive tree statistics for parsimony such as tree length (TL), homoplasy index (HI), consistency index (CI), retention index (RI) and rescaled consistency index (RC) were calculated.

BA were computed with four simultaneous Markov Chain Monte Carlo chains for two runs, 10,000,000 generations and a sampling frequency of 10 generations, ending the run automatically when standard deviation of split frequencies fell below 0.01. The first 25% of trees were discarded as the burn-in fraction, while the remaining 75% were used to calculate the 50% majority rule consensus tree and posterior probability (PP) values.

### 2.4. Phylogenetic Species Recognition

Concatenation methods have been shown to work well with missing data if they are evenly distributed among taxa and gene regions and if a sufficiently large number of genes are sampled [77]. However, the concatenated dataset used to infer the phylogenetic relationships among taxa within the DASC did not have fairly evenly distributed missing data among the five gene regions (Table 1). Thus, given the lack of *cal* and *his3* partial sequences for several species of the DASC, multilocus phylogenetic analyses based on five (ITS, *tef1*, *tub2*, *cal* and *his3*), four (ITS, *tef1*, *tub2* and *cal*) and three (ITS, *tef1* and *tub2*) loci were conducted to properly resolve the species complex. Each analysis included only those species whose five, four and three loci, respectively, were available. Individual gene trees for each of these multilocus phylogenetic analyses conducted were accessed to compare highly supported clades (ML-BS and MP-BS ≥ 70%) in order to detect conflict between the individual phylogenies and to accordingly apply the GCPSR principle [44] to determine the species boundaries of the DASC.

Moreover, the operational criteria of the two-step process described by Dettman et al. [78] were also applied to resolve certain clades which were not clarified after strictly following the GCPSR principle. For these assessments, ML and MP inferences were conducted for single gene sequence alignments. Briefly, the two-step process was applied as follows: clades were genealogically concordant if they were present in at least some of the individual gene genealogies, and genealogically non-discordant if they were well-supported (ML-BS and MP-BS ≥ 70%) in a single gene tree and not contradicted at or above this level of support in more than one other single-gene tree. This criterion prohibited poorly supported non-monophyly at one locus from impairing well-supported monophyly at another locus. In addition, the selected independent evolutionary lineages (IEL) were determined conclusively if resolved with high support values (ML-BS/MP-BS ≥ 70% and PP ≥ 0.95) in most phylogenetic analyses of the combined datasets. Each IEL was ranked as phylogenetic species based on genetic differentiation (lineages must be well-differentiated to prevent minor tip clades from being recognized as phylogenetic species) and exhaustive subdivision (all individuals must be placed into a phylogenetic species to avoid unclassified individuals) criteria [78,79].

ML individual gene trees of the DASC, comprising all available species for each locus, were also constructed to aid conclusions for certain taxa for which a limited number of loci was available and thus were excluded from the multilocus phylogenetic analyses. All phylogenetic inferences included eight well-delimitated outgroup taxa, corresponding to four well-established *Diaporthe* species (*D. citri*, *D. corylicola*, *D. longicolla* and *D. sennicola*).

### 2.5. Phylogenetic Informativeness Analysis

To determine the loci most suitable for phylogenetic inference in the DASC, the phylogenetic informativeness (PI) profiling method [80] was employed. The analysis was implemented in the PhyDesign [81] web server (http://phydesign.townsend.yale.edu/, accessed on 15 June 2023). PI was measured from a partitioned combined dataset of the ITS, *tef1*, *tub2*, *cal* and *his3* loci for 37 isolates, including 20 type strains and related taxa belonging to the DASC and two outgroup taxa. The ML inference from RAxML analysis of the combined dataset was performed using the GTR + G + I substitution model and was used to build a time tree using MEGA X version 10.2.6 [70] as described by Melo [82]. Relative divergence times were estimated for all branching points by applying the RelTime-ML method [83,84] with no calibration constraints. Branch lengths were calculated using the same substitution model as previously used to estimate the phylogenetic tree. The PI for all five partitions were determined using the rates of change for each site under the HyPhy criteria [85].

### 2.6. Coalescent-Based Species Delimitation Analyses

To infer the species boundaries of the DASC, the coalescent-based models Poisson tree processes (PTP) [86] and multi-rate PTP (mPTP), which accommodates different degrees of intraspecific genetic diversity within a phylogeny and has an improved delimitation accuracy compared to the former [87], were performed. Both analyses were conducted using the newick format of the ML inferences produced by FigTree version 1.4.4 [76]. PTP analyses were performed with 500,000 MCMC generations, thinning set to 100, burn-in of 10% and conducted on the web server for PTP (http://species.h-its.org/ptp/, accessed on 15 May 2023). Convergence of the MCMC iterations was assessed by visualizing the log-likelihood trace plot. mPTP analyses were conducted on the web server for mPTP (http://mptp.h-its.org, accessed on 15 May 2023). Including outgroups that are distantly related to the remaining taxa on the phylogenetic inference may worsen the delimitation results provided by the coalescent-based models applied here. Therefore, both analyses were initially run with and without the outgroup taxa to evaluate their impact on the PTP and mPTP species delimitation hypothesis. As results were qualitatively similar, all subsequent analyses were performed with the outgroup taxa to avoid taxonomic discrepancy among analyses. The resulting trees were prepared with Adobe Illustrator CS2 version 12.0.0 (Adobe, San Jose, CA, USA).

Like concatenation methods, coalescent-based species tree estimation methods have been shown to work reliably and produce accurate species trees even when there are substantial amounts of missing data [88], especially if they are randomly distributed (per gene and/or per taxa) and if a sufficiently large number of genes are sampled [77]. Given the lack of *cal* and *his3* partial sequences for several species in the DASC, the coalescent-based PTP and mPTP models applied included those species whose five (ITS, *tef1*, *tub2*, *cal* and *his3*), four (ITS, *tef1*, *tub2* and *cal*) and three (ITS, *tef1* and *tub2*) loci were available, and were conducted using the ML inferences of the 5-, 4- and 3-loci combined datasets, respectively.

### 2.7. Pairwise Homoplasy Index Test and Phylogenetic Network Analyses

The concatenated alignments were used to infer the occurrence of recombination events within the DASC through the pairwise homoplasy index (PHI, Φ_w_) test [89] implemented in SplitsTree4 version 4.19.0 [90]. To detect intragenic recombination, the PHI test was also applied to the single gene sequence alignments. Significant recombination was considered when the probably of the Φ_w_-statistic was below 0.05 (*p*-value < 0.05).

To evaluate and visualize the impact of the potential recombination events, the relationships between closely related taxa within the DASC were visualized through phylogenetic networks based on the concatenated sequence alignments. The phylogenetic networks were constructed using the LogDet transformation [91] for the distance matrix and the Neighbor-Net algorithm [92] implemented using SplitsTree4 version 4.19.0. The resulting phylogenetic networks were prepared with Adobe Illustrator CS2 version 12.0.0 (Adobe, San Jose, CA, USA).

### 2.8. Population Genetic Diversity

Genetic diversity within the DASC was estimated using DnaSP version 6.12.03 [93]. The following molecular diversity indices were calculated for the concatenated and single gene sequence alignments: number of haplotypes (h), number of polymorphic (segregating) sites (S), haplotype (gene) diversity (hd) [94], nucleotide diversity (π) [95], total number of mutations (η) and Watterson estimator (θ) [96]. Neutrality statistical information to understand the potential departure from an equilibrium model of evolution was also obtained through Tajima’s D statistical test [97].

### 2.9. Hierarchical Cluster Analysis of Phenotypic Data

To assess the correlation between species phylogenetic boundaries and taxa morphology, measurements of the length and width of alpha and beta conidia of all species belonging to the DASC with published taxonomic descriptions were used. A hierarchical cluster analysis (HCA) was conducted using R Statistical Software version 4.3.1 [98]. Pairwise distance among taxa were estimated using Euclidean distance index to generate the dissimilarity matrices, and dendrograms were constructed by the unweighted pair group method with arithmetic mean (UPGMA) as the clustering algorithm. Dendrograms were generated using the following R packages: cluster version 2.1.4 [99], factoextra version 1.0.7 [100] and dendextend version 1.17.1 [101]. The optimal number of clusters was determined using the R package nbclust version 3.0.1 [102] according to the majority rule approach. Goodness-of-fit of the dendrograms was evaluated by means of the cophenetic correlation coefficient (c) [103]. Dendrograms were generated based on the length-to-width (L/W) ratios of alpha and beta conidia. These were calculated for all taxa following Equation (1) to standardize and make the data comparable among taxa.

(1) $$L/W=\frac{L_{min}+L_{max}}{W_{min}+W_{max}},$$

where, for a given taxa and a given micromorphological structure, *L_min_* and *L_max_* stand for the length minimum and maximum dimensions, respectively, and *W_min_* and *W_max_* stand for the width minimum and maximum dimensions, respectively.

## 3. Results

### 3.1. Preliminary Phylogenetic Analyses

One hundred twenty-seven isolates of *Diaporthe* species, either from this study or retrieved from GenBank, were included in the phylogenetic analyses (Table 1). The partition homogeneity test for the concatenated alignment resulted in a low *p*-value (*p* = 0.01), indicating that the genes are unsuitable to be combined. Nevertheless, despite the observed incongruences, multilocus analyses were conducted based on the five loci. The ITS, *tef1*, *tub2*, *cal* and *his3* alignment of 119 ingroup and eight outgroup taxa comprised 2124 characters (including alignment gaps) (490 characters for ITS, 341 characters for *tef1*, 376 characters for *tub2*, 461 characters for *cal* and 456 characters for *his3*).

**Figure 1:**
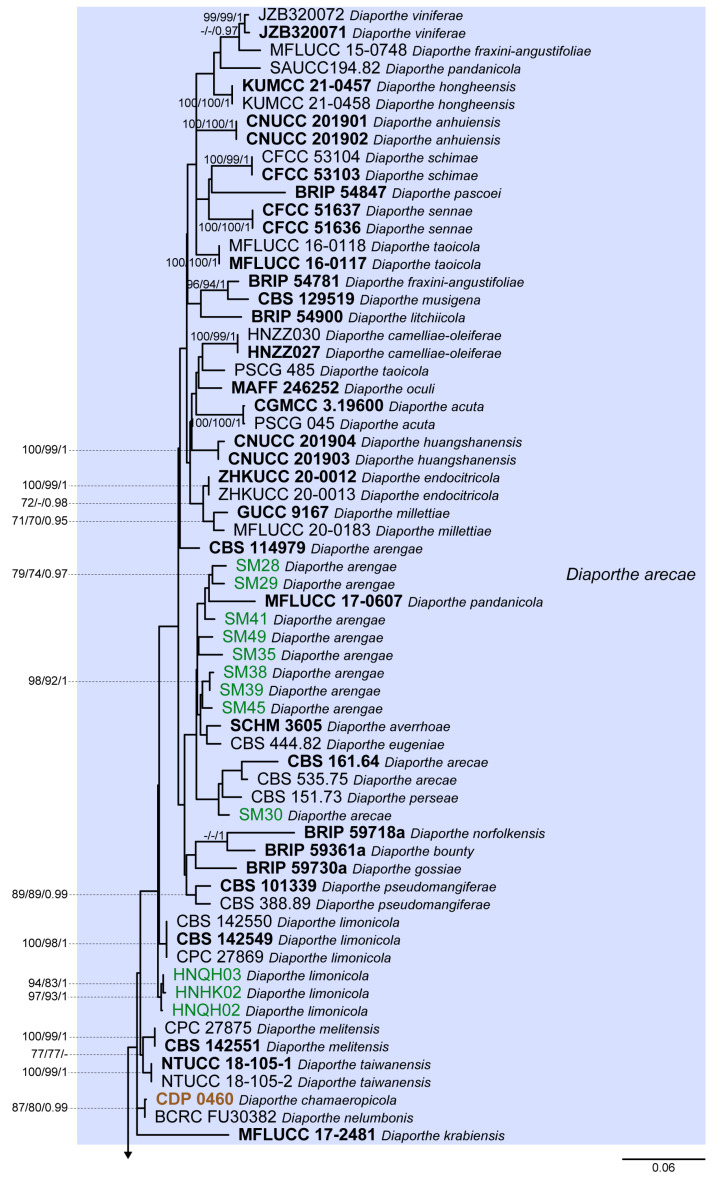

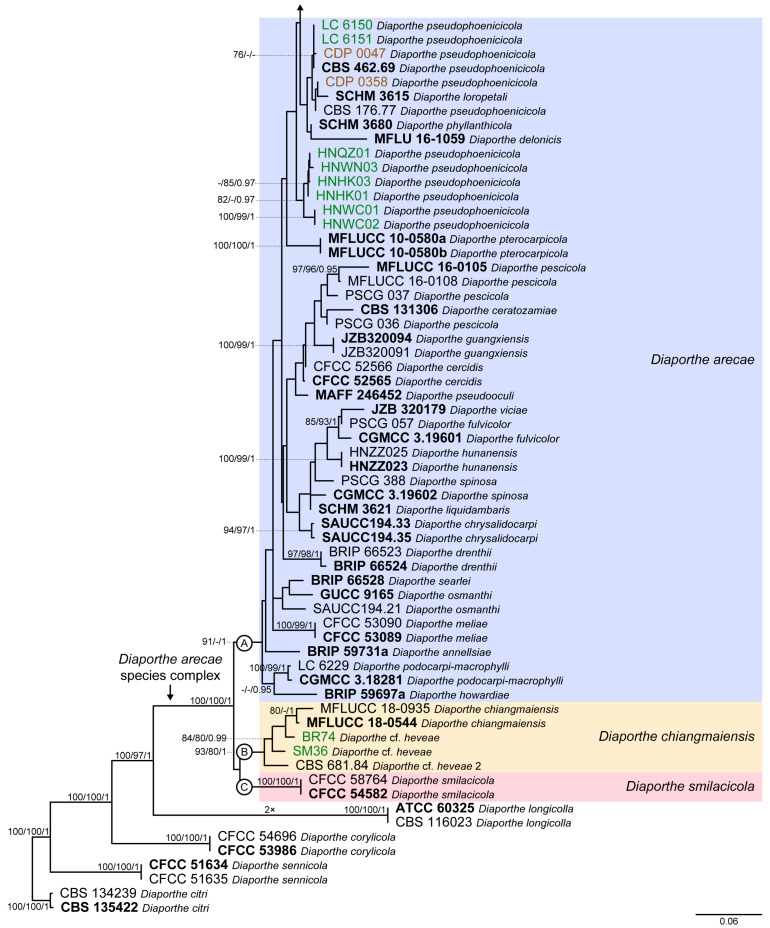
Phylogenetic tree generated from maximum likelihood analysis based on combined ITS, *tef1*, *tub2*, *cal* and *his3* sequence data for the *Diaporthe arecae* species complex and related species. Bootstrap support values for maximum likelihood, maximum parsimony (ML-BS/MP-BS ≥ 70%) and Bayesian posterior probabilities (PP ≥ 0.90) are shown at the nodes. Strains with type status are indicated in bold font. The isolates from this study are presented in brown typeface and the additional isolates from palm tissues included in the analyses are presented in green typeface. Species boundaries within the *D. arecae* species complex are delimited by colored blocks and their respective branches are indicated by lettered circles (A–C). The scale bar represents the expected number of nucleotide changes per site. The tree is rooted to *D. citri* (CBS 134239 and CBS 135422).

Tree topologies resulting from ML, MP and BA inferences were similar, presenting roughly the same well-resolved clades for each species included in the analyses, mostly supported by high maximum likelihood and maximum parsimony bootstrap support values (ML-BS/MP-BS ≥ 70%) and high Bayesian posterior probabilities values (PP ≥ 0.90). The ML tree is shown in Figure 1 with ML-BS/MP-BS/PP values at the nodes.

The final likelihood score for the best scoring ML tree was –15,929.918209. The matrix had 862 distinct alignment patterns, with 27.34% undetermined characters or gaps. Estimated base frequencies were as follows: A = 0.216757, C = 0.321740, G = 0.233831 and T = 0.227672; substitution rates AC = 1.397152, AG = 4.220434, AT = 1.064332, CG = 0.913406, CT = 5.405515 and GT = 1.000000; tree-length = 2.835210; gamma distribution shape parameter α = 0.494944; and proportion of invariable sites = 0.416215. BA inference had an average standard deviation of split frequencies (SDSF) and an average potential scale-reduction factor (PSRF) of 0.074169 and 1.012, respectively, after 10,000,000 generations, resulting in 1,500,002 trees being sampled.

Concerning MP analysis, of the 2124 characters, 1336 characters were constant (62.9%), and 107 variable characters were parsimony uninformative. MP analysis of the remaining 681 parsimony-informative characters (32.1%) resulted in 1000 equally parsimonious trees of 2447 steps with a moderate level of homoplasy as indicated by a CI of 0.449, HI of 0.551, RI of 0.750 and RC of 0.337. The topology of trees differed from one another only in the positions of the isolates within terminal groupings.

According to the phylogenetic analyses of the concatenated alignment (Figure 1), the three isolates from this study, obtained from foliar lesions of palms, clustered in a highly supported monophyletic clade (100% ML-BS/100% MP-BS/1 PP) containing 57 species, which is designated here as the *D. arecae* species complex (DASC). Moreover, three well-supported sister subclades were observed within the DASC, which were noted as subclades A, B and C (Figure 1). The three isolates from this study, along with 20 strains from palm tissues, clustered together in a subclade comprising 55 species with high ML-BS/PP support values (91%/1; subclade A). The remaining two strains from palm tissues clustered in a highly supported subclade (93% ML-BS/81% MP-BS/1 PP) together with *D. chiangmaiensis* (MFLUCC 18-0935 and MFLUCC 18-0544, ex-type) and the strain CBS 681.84 (“*Diaporthe* cf. *heveae 2*”) (subclade B). Subclade C corresponds to *D. smilacicola* (CFCC 54582, ex-type, and CFCC 58764), which form a highly supported branch (100% ML-BS/100% MP-BS/1 PP) in the DASC. The subclades A, B and C identified are here reported as three putative phylogenetic species—*D. arecae*, *D. chiangmaiensis* and *D. smilacicola*—and further analyses were conducted to validate their species boundaries.

The ML individual gene trees of the DASC comprising all available species for each locus (Figures S1–S5) also showed that the isolates from this study, along with other strains from palm tissues, clustered in a monophyletic clade with high ML-BS values (94%, 100%, 93%, 72% and 84% in ITS-, *tef1*-, *tub2*-, *cal*- and *his3*-phylogram, respectively). Thus, the DASC as defined in the present study was similarly observed in all individual gene genealogies. Nonetheless, tree topologies between the individual gene trees varied substantially and most of the internal nodes received low bootstrap support. Moreover, individual gene trees, except for the *his3*-phylogram, failed to clearly resolve the three subclades structure of the DASC as observed in the multilocus phylogenetic analyses (Figure 1 and Figures S1–S5). In general, tree topology of the *his3*-phylogram (Figure S5), and to a lesser extent of the *cal*-phylogram (Figure S4), were more similar to the phylogenetic analyses of the combined dataset. The multilocus phylogenetic analyses showed a better delimitation of the DASC when compared to the individual gene genealogies.

### 3.2. Species Delimitation Based on the GCPSR Principle

Although in the present study five loci were used to infer the phylogenetic relationships among taxa within the DASC, many taxa were missing sequences of *his3* and *cal* loci (Table 1). These loci were not available for 62 (49%) and 44 (35%) strains, respectively, out of the 127 taxa included in the analyses, while only nine (7%) strains did not have sequences of *tub2* and/or *tef1* loci.

**Figure 2:**
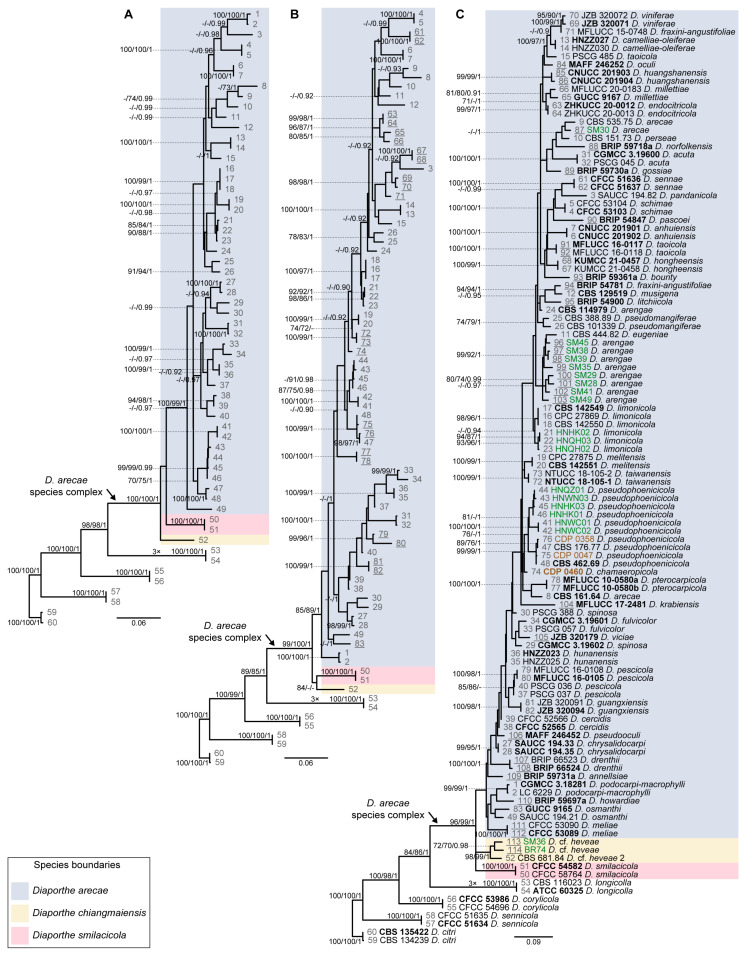
Phylogenetic trees generated from maximum likelihood analysis of the *Diaporthe arecae* species complex and related species. (**A**). Based on combined dataset of 5 loci (ITS, *tef1*, *tub2*, *cal* and *his3*). (**B**). Based on combined dataset of 4 loci (ITS, *tef1*, *tub2* and *cal*). (**C**). Based on combined dataset of 3 loci (ITS, *tef1* and *tub2*). Bootstrap support values for maximum likelihood, maximum parsimony (ML-BS/MP-BS ≥ 70%) and Bayesian posterior probabilities (PP ≥ 0.90) are shown at the nodes. Taxa numbers were generated, and the corresponding strains and species are shown in the 3-loci phylogram (panel (**C**)). Underlined numbers denote taxa that were excluded in the previous dataset due to lack of sequence data. Strains with type status are indicated in bold font. The isolates from this study are presented in brown typeface and the additional isolates from palm tissues included in the analyses are presented in green typeface. Species boundaries within the *D. arecae* species complex are delimited with colored blocks and referred to in the chart legend. The scale bars represent the expected number of nucleotide changes per site. The trees are rooted to *D. citri* (CBS 134239 and CBS 135422).

Given the lack of *cal* and *his3* sequences for several species of the DASC, multilocus phylogenetic analyses were also conducted based on combined datasets of five (ITS, *tef1*, *tub2*, *his3* and *cal*), four (ITS, *tef1*, *tub2* and *cal*) and three loci (ITS, *tef1* and *tub2*) to properly aid conclusions about the species for which those loci were missing on the primary combined dataset phylogenetic analyses (Figure 1). Thus, each analysis included only the species whose respective loci were available. The partition homogeneity test for the five-, four- and three-loci concatenated alignments resulted in low *p*-values (*p* = 0.01), indicating that the genes are unsuitable to be combined. Nevertheless, despite the observed incongruences, multilocus ML, BA and MP phylogenetic inferences were conducted for the five-, four- and three-loci combined datasets, and the resulting trees were compared. The ML trees are shown in Figure 2 with ML-BS/MP-BS/PP values at the nodes. Moreover, the single gene genealogies corresponding to each combined dataset were analyzed separately using ML and MP inferences. Tree topologies (Figures S6–S8) were also compared to evaluate phylogenetic congruencies in the DASC through the implementation of the GCPSR principle. Statistics for the different datasets and respective phylogenetic trees are summarized in Table 2.

The combined datasets of five, four and three loci included 52, 75 and 106 ingroup, and eight outgroup taxa and comprised 2124, 1668 and 1207 characters (including alignment gaps), respectively (Table 2). The ML, MP and BI inferences for each combined dataset resulted in topologically similar trees. All three combined datasets produced trees with a similar backbone structure (Figure 2), which was also similar to that obtained for the primary combined dataset phylogenies (Figure 1). Overall, a highly supported monophyletic clade corresponding to the DASC was obtained on the five- (100% ML-BS/100% MP-BS/1 PP) (Figure 2A), four- (99% ML-BS/100% MP-BS/1 PP) (Figure 2B) and three-loci phylogram (96% ML-BS/99% MP-BS/1 PP) (Figure 2C), each presenting the three monophyletic subclades as noted for the primary combined dataset phylogenies (Figure 1). Therefore, tree topologies resulting from the five-, four- and three-loci combined datasets were congruent and recognized three putative phylogenetic species within the DASC, namely *D. arecae*, *D. smilacicola* and a clade comprising the strains identified as “*Diaporthe* cf. *heveae*” (Figure 2).

Although “*Diaporthe* cf. *heveae*” strains have been putatively recognized as *D. chiangmaiensis* in the primary combined dataset phylogenies (Figure 1), no partial *tub2*, *cal* and *his3* sequence data were available for *D. chiangmaiensis* (MFLUCC 18-0935 and MFLUCC 18-0544, ex-type). Therefore, these two strains were excluded from all three combined dataset analyses. Since only ITS and *tef1* sequence data were available for the above-mentioned strains of *D. chiangmaiensis*, a multilocus ML phylogenetic analysis was conducted for all the taxa for which those two loci were available to aid conclusions regarding the relationship between *D. chiangmaiensis* and “*Diaporthe* cf. *heveae*”. The tree obtained presented a highly supported monophyletic clade (98% ML-BS) with three well-supported sister subclades, confirming the predictions of all previous phylograms constructed. Moreover, the *D. chiangmaiensis* strains MFLUCC 18-0544 (ex-type) and MFLUCC 18-0935 clustered with the “*Diaporthe* cf. *heveae*” strains with high ML-BS support (99%), similar to what was obtained in the primary combined dataset phylogenies (Figure 1).

According to the inferences based on the combined datasets of four and three loci, isolates from this study clustered in the *D. arecae* subclade, together with other strains isolated from palm tissues (Figure 2B,C). The combined phylogenetic analyses suggested that the *D. arecae* subclade may putatively represent a single species sister to *D. smilacicola* and *D. chiangmaiensis*. Most independent evolutionary branches within the *D. arecae* subclade showed a low or complete lack of support values and only terminal branches for some of the species clustered in highly supported clades (Figure 1 and Figure 2).

To understand the boundaries of the DASC, the GCPSR principle was followed, and the individual ML and MP gene trees produced for each of the combined datasets were compared to identify concordant branches. All individual ML and MP gene trees were topologically similar, presenting the same well-delimited clades. However, this analysis also revealed conflicts between the individual phylogenies, with incongruent branches and most nodes lacking phylogenetic support (Figures S6–S8). Considering the individual phylogenies corresponding to the five-loci combined dataset (Figure S6), it is evident that isolates from the same species cluster in different clades depending on the individual gene tree. For instance, two isolates of *D. arecae*, including the ex-isotype strain CBS 161.64, are phylogenetically distant in the ITS phylogram (Figure S6A), while they group together in the remaining individual phylograms (Figure S6B–E). Likewise, two isolates of *D. pseudomangiferae*, including the ex-type strain CBS 101339, are paraphyletic in the *tef1* and *cal* phylogram (Figure S6B,D), but cluster together in a highly supported monophyletic branch in the remaining individual phylogenies (Figure S6A,C,E). Moreover, the relationships between different species are highly discordant among the individual phylogenies. For example, while *D. melitensis* is phylogenetically indistinguishable from *D. limonicola* in the *tef1*, *cal* and *his3* phylograms (Figure S6B,D,E), they are phylogenetically distant in both ITS and *tub2* individual phylogenies (Figure S6A,C). Moreover, *D. perseae*, *D. eugeniae* and *D. musigena* are closely related to *D. arecae* in the *tef1* phylogram (Figure S6B), but are distributed throughout the remaining individual phylograms, clustering with other species (Figure S6A,C–E). A similar pattern of incongruencies was observed for the individual phylogenies corresponding to the combined datasets of four and three loci (Figures S7 and S8). In both cases, the greater the number of taxa included in the analyses, the greater the inconsistencies between the individual phylogenies. For instance, *D. viniferae* clusters in a highly supported monophyletic clade in the ITS and *tub2* phylogram (Figures S7A,C and S8A,C), while it is phylogenetically indistinguishable from *D. guangxiensis*, *D. camelliae-*oleiferae (Figure S7B) and *D. viciae* (Figure S8B) in the *tef1* phylogram and from *D. guangxiensis* and *D. cercidis* in the *cal* phylogram (Figure S7D).

Following the GCPSR principle, based on the comparison of individual gene genealogies, it was verified that the node delimiting the transition from concordant branches to incongruencies corresponds to the DASC (Figure 2). Contrarily, individual gene trees are concordant regarding the four well-delimited species (*D. citri*, *D. corylicola*, *D. longicolla* and *D. sennicola*) included as outgroup taxa, and represented by highly supported monophyletic clades (Figures S6–S8). This provides solid evidence that these clades represent different species as opposite to the different species included in the DASC.

To further resolve the putative phylogenetic species previously recognized as three distinct well-supported subclades within the DASC (Figure 1 and Figure 2), an operational framework to identify independent evolutionary lineages (IEL) was applied. Due to the presence of discordant nodes, conflicting branches and a lack of phylogenetic support between taxa of the *D. arecae* subclade among all individual gene genealogies, subclade A was recognized as a single IEL following the criteria of genealogical concordance and genealogical non-discordance. The backbone structure of three well-supported subclades (A, B and C) within the DASC observed in the combined datasets (Figure 1 and Figure 2) were noted in both the *his3* (Figure S6) and *cal* phylogram (Figures S6 and S7), which was also observed in the initial individual gene trees (Figures S4 and S5). Although these well-supported subclades were not recovered from ITS, *tef1* and *tub2* individual phylogenies (Figures S6–S8), strains of *D. smilacicola* and “*Diaporthe* cf. *heveae*” formed two monophyletic IEL in all individual phylogenies, except for the *tub2* phylogram from the combined dataset of three loci (Figure S8C). Thus, the GCPSR principle also supports the existence of three putative phylogenetic species within the DASC, with most strains falling into the *D. arecae* subclade that seems to represent a single phylogenetic species sister to *D. smilacicola* and *D. chiangmaiensis*.

As estimated by the initial ITS, tef1 or tub2 phylograms (Figures S1–S3), the species *D. averrhoae*, *D. ceratozamiae*, *D. delonicis*, *D. liquidambaris*, *D. loropetali*, *D. nelumbonis*, *D. phyllanthicola*, *D. searlei*, and the ex-type strain of *D. pandanicola* (MFLUCC 17-0607), belong to the DASC, more exactly to the *D. arecae* subclade. However, given the limited number of loci available for these species, they were not included in the five-, four- and three-loci combined datasets. Nonetheless, considering the structure of the individual gene trees (Figures S1–S3), and given the position of the aforementioned species within the DASC, it is here advocated that they should be assigned to *D. arecae*.

### 3.3. Phylogenetic Informativeness and Informative Characters of Each Locus

**Figure 3:**
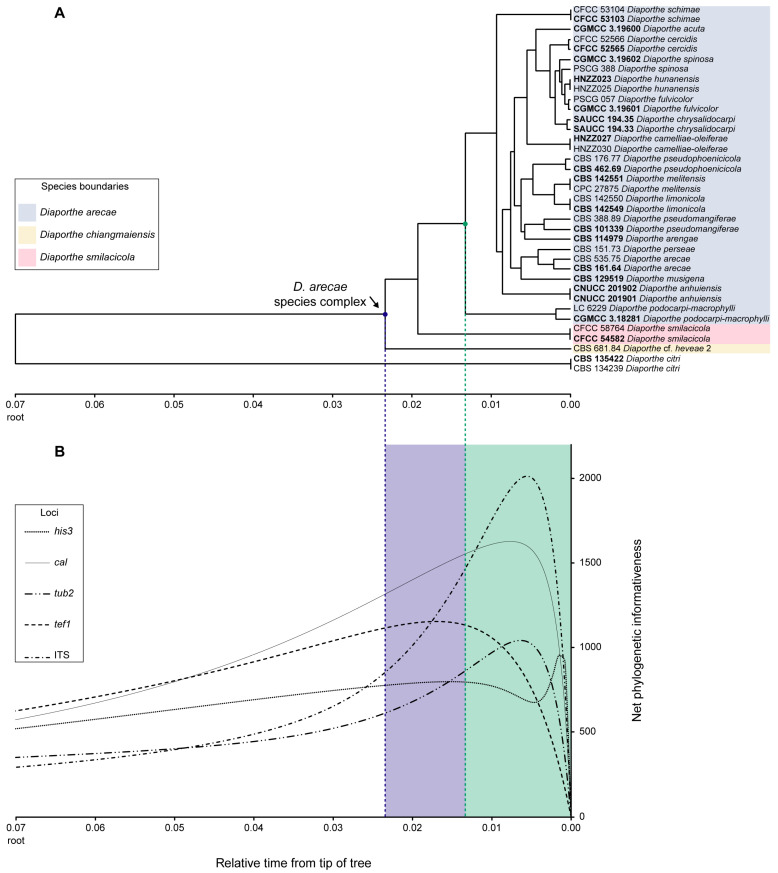
Phylogenetic informativeness profiles of the *Diaporthe arecae* species complex and related species through a relative time scale for a combined dataset of five loci (ITS, *tef1*, *tub2*, *cal* and *his3*). (**A**). Time tree inferred by applying the RelTime-ML method. All divergence times shown are relative times as no calibrations were used. Strains with type status are indicated in bold font. Species boundaries within the *D. arecae* species complex are delimited by colored blocks and referred to in the chart legend. The tree is rooted to *D. citri* (CBS 134239 and CBS 135422). (**B**). Net phylogenetic informativeness profiles in arbitrary units matched to the time tree scale with lines representing individual loci profiles and referred to in the chart legend. Nodes of interest are highlighted by blue and green dots and dashed lines through both panels, and the corresponding graph areas are emphasized by colored blocks.

The Phylogenetic Informativeness (PI) profiles indicated that, in general terms, *cal*, *tef1* and *his3* are the most informative markers for phylogenetic inference of the DASC, while ITS and *tub2* are the least informative (Figure 3). Integrating PI over specific periods of time provides information for ranking loci. The PI analysis showed a peak for the ITS curve corresponding to the *D. arecae* subclade (green dot and dashed line in Figure 3) and for that specific relative period of time ITS ranks as the most informative marker. Nonetheless, ITS is the least informative locus as the tree approaches its root. According to the informative characters provided by the phylogenetic analyses, ITS displayed the least informative sequences, with the lowest percentage of parsimony-informative characters (17.3%) and unique alignment patterns (23.1%) (Table 2, 5-loci dataset), suggesting that this locus might not be suitable for species delimitation within the DASC. However, phylogenetic analyses excluding the ITS locus were performed in the present study and, except for a slightly improvement in the support values for some nodes, the backbone structure of the trees obtained was similar to those in which the ITS locus was included.

Opposite to ITS, *cal*, *tef1* and, to a lesser extent, *his3* ranked as the most informative loci to infer species limits of the DASC (blue dot and dashed line in Figure 3) and to resolve the backbone structure of three well-supported subclades observed in the multilocus phylogenetic inferences (Figure 1 and Figure 2), which is congruent with the results obtained for the *his3* and *cal* phylogram (Figures S4–S7). In comparison with the percentage of parsimony-informative characters and unique alignment patterns of each locus (Table 2, five-loci dataset), *tef1* (48.4% and 52.3%, respectively) and *cal* (33.6% and 39.7%, respectively) showed a congruent result with the PI profiles as the most informative loci. Nonetheless, although the PI profile of *tub2* was apparently one of the least informative to resolve species boundaries in the DASC, it exhibited some value in terms of the percentage of parsimony-informative characters (30.6%) and unique alignment patterns (35.4%) (Table 2, 5-loci dataset), ranking as the third out of five most informative loci for phylogenetic inference in the DASC.

The increase in the number of taxa, and subsequently increase the amount of data in each locus, from the five- to the three-loci datasets, we increased the amount of homoplasy detected in each locus (Table 2). For instance, according to the descriptive tree statistics provided by the MP analyses, ITS presented an increasingly moderate level of homoplasy in all three analyses from 0.44 to 0.51 (Table 2, five- and three-loci datasets, respectively). Similarly, while the remaining loci presented low level of homoplasy in the five- and four-loci dataset analyses, *tef1* and *tub2* presented moderate levels of homoplasy in the three-loci dataset analysis (0.41 and 0.43, respectively; Table 2, three-loci dataset). Homoplasy may arise from reticulation events during the evolutionary history and, as a consequence, can be seen as an indirect measure of recombination. Therefore, increasing the number of taxa seems to reveal the presence of recombination within the DASC and further analyses were conducted to validate this hypothesis.

### 3.4. Species Delimitation Based on Poisson Tree Processes Models

As previously referred to, missing data were very unevenly distributed among the different genes used, corresponding mostly to sequences of *his3* and *cal* loci (Table 1). Given the lack of these data for several species of the DASC, the coalescent-based PTP and mPTP models applied included those species whose five, four or three loci were available. Therefore, the analyses were conducted using the ML inferences of the five-, four- and three-loci combined datasets, respectively (Figure 2).

**Figure 4:**
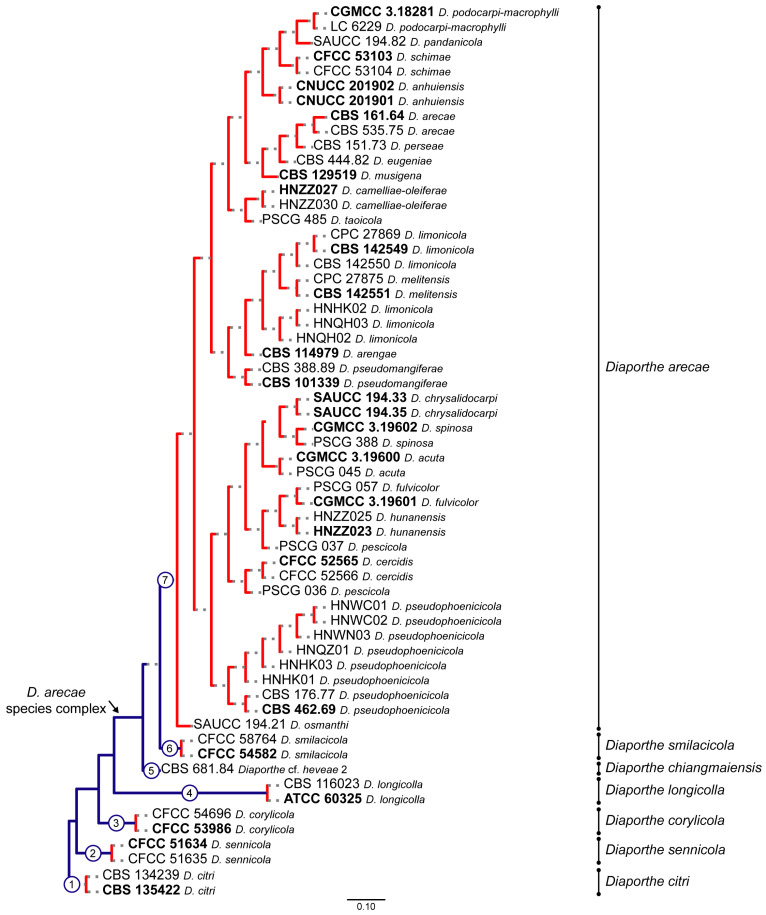
Maximum-likelihood species delimitation scheme obtained from the Poisson tree process (PTP) analysis of the *Diaporthe arecae* species complex and related species, based on combined dataset of 5 loci (ITS, *tef1*, *tub2*, *cal* and *his3*). Blue-colored branches illustrate the speciation process and red-colored branches illustrate the coalescent/population process. Putative species clusters are represented as transitions from blue-colored to red-colored branches or as terminal, blue-colored branches and are highlighted by numbered circles (1–7). Strains with type status are indicated in bold font. The scale bar represents the expected number of nucleotide changes per site.

**Figure 5:**
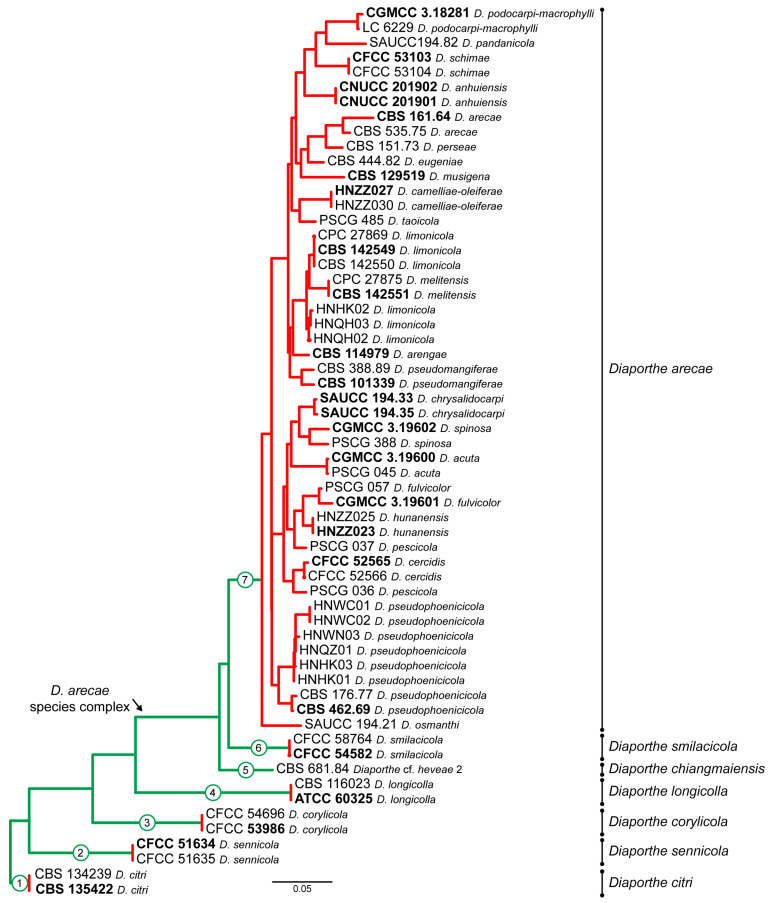
Maximum-likelihood species delimitation scheme obtained from the multi-rate Poisson Tree Process (mPTP) analysis of the *Diaporthe arecae* species complex and related species, based on combined dataset of 5-loci (ITS, *tef1*, *tub2*, *cal* and *his3*). Green-colored branches illustrate the speciation process and red-colored branches illustrate the coalescent process. Putative species clusters are represented as transitions from green-colored to red-colored branches or as terminal, green-colored branches and are highlighted by numbered circles (1–7). Strains with type status are indicated in bold font. The scale bar represents the expected number of nucleotide changes per site.

The PTP and mPTP analyses performed gave congruent species delimitation results both for each combined dataset and between the different combined datasets. Only the PTP and mPTP trees with a species delimitation hypothesis obtained for the combined dataset of five loci are shown in Figure 4 and Figure 5, respectively, as illustrative results. The web server for PTP outputs a maximum likelihood solution and a highest Bayesian supported solution as species delimitation schemes. The highest Bayesian solution or bPTP corresponds to a Bayesian implementation of the original maximum likelihood PTP model for species delimitation (https://species.h-its.org/ptp/, accessed on 15 May 2023) and adds Bayesian support values to delimited species on the input tree. Although both solutions obtained in the present study gave congruent species delimitation results for all the combined datasets tested, with moderate acceptance rates of more than 60%, the Bayesian support values were inconsistent between the different combined datasets and most were below 0.9. Taking into consideration that the web server for PTP has a limit of 500,000 MCMC generations, the low Bayesian support values might be related to a lack of sufficient MCMC iterations to produce more accurate support values. Therefore, to avoid reporting meaningless results, only the maximum likelihood solution is provided in Figure 4.

According to the estimated species trees, the transition from blue-colored to red-colored branches (in PTP, Figure 4), and the transition from green-colored to red-colored branches (in mPTP, Figure 5) was evidence that both coalescent-based methods returned ML partitions of seven putative species. Both analyses inferred three putative species within the *D. arecae* species complex. Both models recognized that all species within the *D. arecae* subclade were comprised in a single monophyletic branch, i.e., they constitute a single species, and thus the strains should be considered as individuals within a population, rather than different taxa. Moreover, PTP and mPTP analyses also showed concordant results regarding the four well-delimited species included as an outgroup and recognized these taxa as monophyletic clades. Therefore, the results obtained with the coalescent-based methods were consistent with the phylogenetic inferences of the DASC (Figure 1 and Figure 2) and the results obtained following the GCPSR principle.

To properly assist in the phylogenetic relationship between the *D. chiangmaiensis* and “*Diaporthe* cf. *heveae*” strains, PTP and mPTP analyses were performed based on the combined dataset of ITS and *tef1* sequence data due to the lack of *tub2*, *cal* and *his3* sequences for these taxa. Both analyses gave similar results, and only the mPTP tree with the species delimitation hypothesis is shown in Figure S9.

The mPTP species delimitation result obtained was congruent with the previous coalescent-based analyses and inferred a ML partition of seven putative species. Moreover, considering the transition between green-colored and red-colored branches, the mPTP analysis recognized *D. chiangmaiensis* and “*Diaporthe* cf. *heveae*” as conspecific, as previously predicted.

### 3.5. Pairwise Homoplasy Test and Phylogenetic Network Analyses

The PHI test performed on the five-, four- and three-loci combined datasets gave congruent results and found statistically significant evidence for recombination (*p* = 0.00, Table 3), denoting that there is no reproductive isolation within the DASC. Moreover, the PHI test also revealed that ITS and *tef1* loci are subjected to a significant rate of recombination on the combined dataset of five (*p* = 4.34 × 10^−4^ and 0.02, respectively), four (*p* = 0.01 and 2.94 × 10^−3^, respectively) and three loci (*p* = 0.02 and 1.12 × 10^−3^, respectively) (Table 3). Likewise, the *tub2* locus tested positive for recombination on the combined dataset of four loci (*p* = 9.92 × 10^−3^), although no recombination was detected when performing the combined datasets of five and three loci (*p* = 0.07 and 0.23, respectively) (Table 3). The results obtained are congruent with the predicted occurrence of recombination by the measures of homoplasy provided by the MP analyses (Table 2).

**Figure 6:**
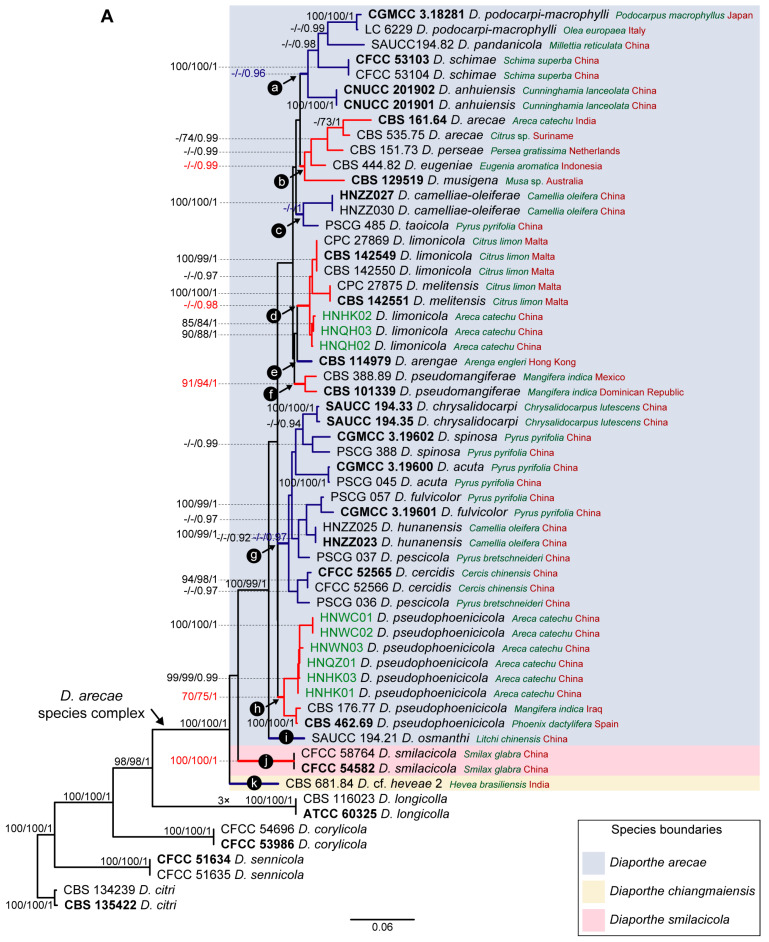

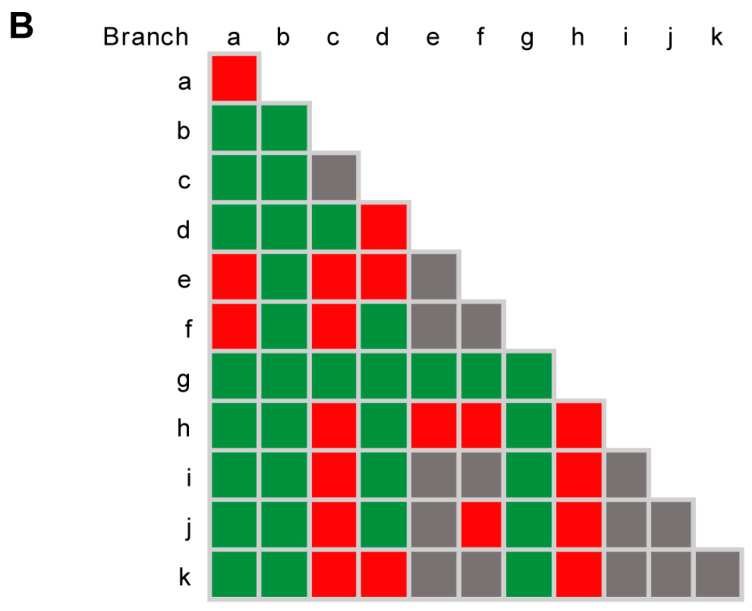
Pairwise homoplasy index (PHI) test for recombination among taxa within the *Diaporthe arecae* subclade. (**A**). Phylogenetic tree generated from maximum likelihood analysis of the *D. arecae* species complex and related species based on combined dataset of 5 loci (ITS, *tef1*, *tub2*, *cal* and *his3*). Bootstrap support values for maximum likelihood, maximum parsimony (ML-BS/MP-BS ≥ 70%) and Bayesian posterior probabilities (PP ≥ 0.90) are shown at the nodes. Strains with type status are indicated in bold font. The additional isolates from palm tissues included in the analyses are presented in green typeface. Species names within the *D. arecae* species complex are followed by the host genus/species from which it was isolated (green) and the country of origin (red). Species boundaries within the *D. arecae* species complex are delimited by colored blocks and referred to in the chart legend. Black circles represent hypothetical populations or “species” within the *D. arecae* species complex as inferred by either the support values of ML, MP or BA. The support values and the branches of each hypothetical population or “species” are highlighted by alternating blue and red colours. The scale bar represents the expected number of nucleotide changes per site. The tree is rooted to *D. citri* (CBS 134239 and CBS 135422). (**B**). Matrix of recombination results of paired branches in the *D. arecae* species complex based on combined dataset of 5 loci (ITS, *tef1*, *tub2*, *cal* and *his3*). Green squares indicate positive results for recombination (*p* < 0.05), red squares indicate negative results for recombination (*p* ≥ 0.05) and grey squares indicate that the PHI test was not performed as only one isolate was available, or there were too few informative characters.

To further analyze the occurrence of recombination among taxa within the *D. arecae* subclade, highly supported monophyletic branches or singletons by either ML-BS, MP-BS or PP from the phylogenetic inference of the five-loci combined dataset were selected as hypothetical populations or “species”, respectively, and the PHI test was performed for and between every pair of branches (branch a to i, Figure 6A). Moreover, the PHI test was also performed between these monophyletic branches and *D. chiangmaiensis* and *D. smilacicola* subclades (branch j and k, Figure 6A). The matrix of the recombination results is shown in Figure 6B.

Most well-supported branches in the complex showed a wide geographical distribution and were not restricted to a specific locality or host plant (Figure 6A). Even so, an exception is observed in branches c and g, which include taxa that were exclusively collected from different provinces of China, although associated with a variety of plant hosts (Figure 6A). However, significant recombination was detected within branches b and g (*p* = 6.85 × 10^−8^ and 5.41 × 10^−10^, respectively, Figure 6B), revealing the absence of reproductive isolation between *D. arecae*, *D. eugeniae*, *D. musigena* and *D. perseae*, and *D. acuta*, *D. cercidis*, *D. chrysalidocarpi*, *D. fulvicolor*, *D. hunanensis*, *D. pescicola* and *D. spinosa*, respectively (Figure 6A). Moreover, all tested paired branches that included the b or g branches gave positive results for recombination, which are likely to be influenced by the presence of significant recombination among the taxa that compose those branches.

Nonetheless, many other paired branches tested positive for recombination without significant recombination within the branches themselves (Figure 6B). For instance, significant recombination was found among taxa from branch a (isolated from five different hosts and three countries) and the species *D. osmanthi* (branch i, isolated from Litchi chinensis in China) (*p* = 1.40 × 10^−5^); among taxa from branch d (isolated from *Citrus limon* in Malta and *Areca catechu* in China) and the species *D. pseudomangiferae* (branch f, isolated from *Mangifera indica* in Mexico and Dominican Republic) (*p* = 3.86 × 10^−2^) and *D. osmanthi* (branch i) (*p* = 4.53 × 10^−2^); and among taxa from branches a and c (isolated from *Camellia oleifera* and *Pyrus pyrifolia*) and the species *D. pseudophoenicicola* (branch h, isolated from *A. catechu* in China, *M. indica* in Iraq and *Phoenix dactylifera* in Spain) (*p* = 1.00 × 10^−2^ and 2.00 × 10^−3^, respectively) (Figure 6). Significant recombination was also detected between *D. chiangmaiensis* (branch k, isolated from *Heveae brasiliensis* in India) and *D. smilacicola* (branch j, isolated from *Smilax glabra* in China) and taxa from branch a (*p* = 9.80 × 10^−4^ and 1.90 × 10^−2^, respectively), as well as between *D. smilacicola* and taxa from branch d (*p* = 2.76 × 10^−2^) (Figure 6).

**Figure 7:**
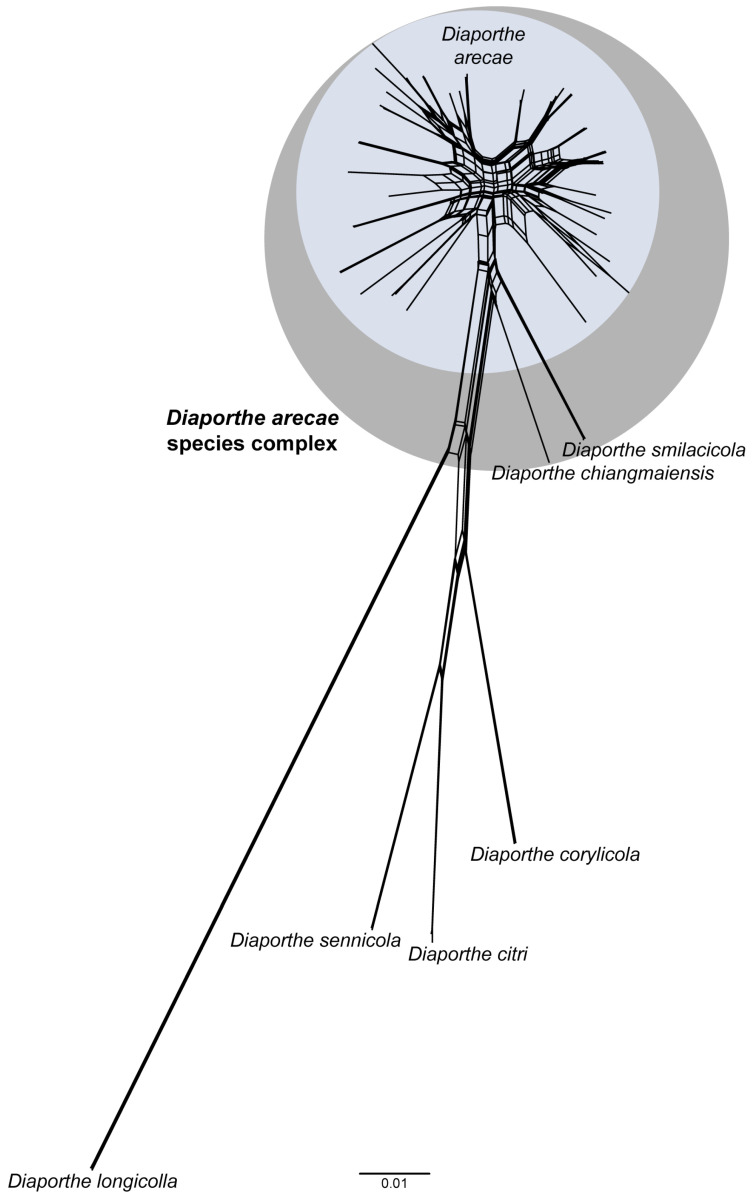
NeighborNet phylogenetic network of the *Diaporthe arecae* species complex and related species based on the LogDet transformation for a combined dataset of 5 loci (ITS, *tef1*, *tub2*, *cal* and *his3*). *Diaporthe arecae* species complex and *D. arecae* are delimited by colored blocks. The scale bar represents the expected number of nucleotide changes per site.

The phylogenetic networks built for the combined dataset of five, four and three loci gave very similar results and showed fit values greater than 99% (fit = 99.84%, 99.71% and 99.43%, respectively), indicating that the displayed networks represent well the LogDet distance matrices from which they were computed. Only the splits-graph for the combined dataset of five loci is shown in Figure 7, as an illustrative result. According to the networked relationships, the DASC presents many contradicting edges, representing incompatible and ambiguous signals within the dataset.

These conflicting signals are particularly present among taxa belonging to the *D. arecae* subclade (subclade A in Figure 1), where parallel edges and boxlike polygons are found between virtually all taxa, revealing the presence of reticulate events, such as recombination, within the group. On the contrary, the four well-delimited species included as outgroup taxa are clearly placed apart from the DASC by an assemblage of long branches and bifurcating evolutionary relationships (Figure 7). Thus, the presence of boxlike polygons in the networked relationships among taxa of the *D. arecae* subclade imply likelihood of recombination between them, suggesting, together with the relative distances of taxa, that all strains within the *D. arecae* subclade should be regarded as conspecific.

The phylogenetic network analyses were congruent with the previous phylogenetic inferences and the GCPSR principle, regarding the existence of three distinct species within the DASC. While the networked relationships among taxa of the *D. arecae* subclade appear to exhibit inherently non-treelike evolutionary events, the relative distance and phylogenetic network structure of the branches corresponding to *D. chiangmaiensis* (subclade B in Figure 1) and *D. smilacicola* (subclade C in Figure 1) seem to clearly approach a bifurcated evolutionary relationship without conflicting phylogenetic signals, i.e., without recognition of expressive recombination.

Although similar topological results were observed between the phylogenetic networks built for the different combined datasets, according to what was observed in the previous analyses, the increase in the number of taxa within the DASC, amplified the deviations from a treelike pattern for the *D. arecae* subclade, revealing a higher number of conflicting phylogenetic signals illustrated by parallel edges. These increasing conflicting signals among isolates within the *D. arecae* subclade in successive analyses are in line with the extensive topological incongruences of previous analyses and further suggests that it should be regarded as a single species.

### 3.6. Population Genetic Diversity

Molecular diversity indices and the Tajima’s D test for neutrality were computed for individual gene alignments and concatenated alignments of each combined dataset (five-, four- and three-loci) for the DASC, and a summary of the genetic diversity is presented in Table 4. Overall, the increasing of sample size, i.e., the number of sequences (taxa) in the combined datasets, led to an increase in genetic diversity. However, the observed results were congruent among the three combined datasets and therefore only those for the combined dataset of five loci will be quoted here.

The analyses of genetic diversity within the DASC showed a considerable number of haplotypes (h), segregating sites (S) and mutations (η) for each individual locus and for the concatenated loci. Nonetheless, *cal* and *tef1* presented the highest number of haplotypes (33 and 21, respectively), segregating sites (73 and 72, respectively) and mutations (91 and 75, respectively) (Table 4), which is congruent with the previous analyses that depicted these loci as the most informative to resolve the DASC (Table 2, Figure 3). All loci presented high haplotype diversity (Hd), but low nucleotide diversity (π), suggesting population expansion for the DASC. While haplotype diversity values for each locus and for the combined loci were greater than 95%, reflecting high genetic diversity; the same was not reflected by the nucleotide diversity values, which ranged from 2.1% to 3.7%.

Population expansion in the DASC was also suggested by the neutrality results of the Tajima’s D test, which presented negative values for all loci and for the combined dataset, although associated probabilities did not reach statistical significance (Table 4). However, Tajima’s D test showed a statistically significant difference from the neutral expectations at the 5% level for *cal* (4-loci dataset in Table 4), *tef1* and *tub2* (3-loci dataset in Table 4). Thus, the significant departure from neutrality appears to be influenced by the number of taxa included in the DASC, indicating that a similar result would likely be obtained for the combined dataset of five loci if all sequences were available for all species. In addition, comparisons between the nucleotide diversity (π) values and the Watterson estimator (θ) values, which is an expectation of π, also suggest a departure from neutrality in the DASC, since those values are different for most datasets tested.

### 3.7. Hierarchical Cluster Analysis of Phenotypic Data

**Figure 8:**
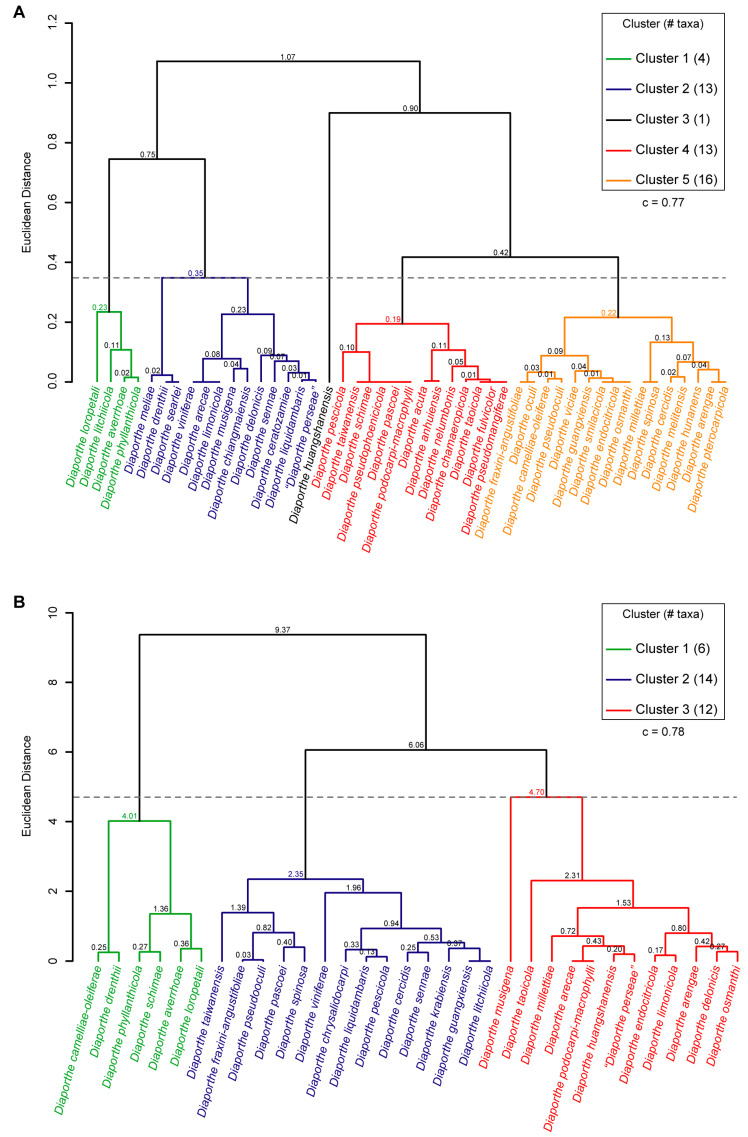

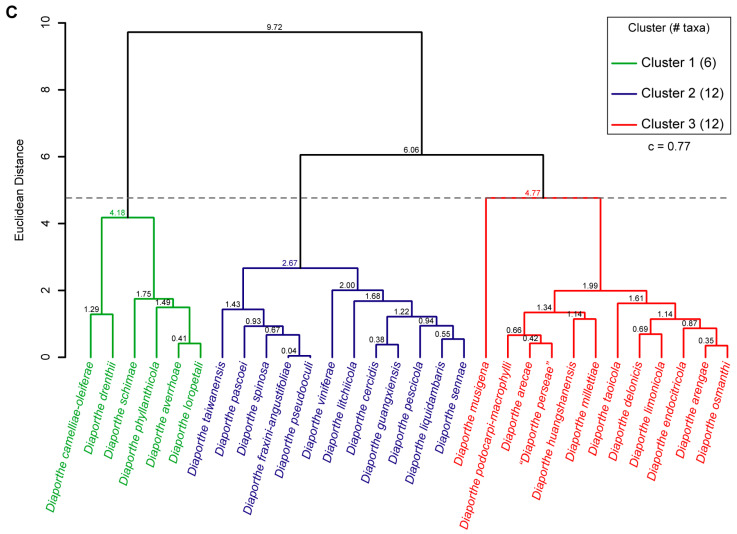
Dendrograms obtained by hierarchical cluster analysis of the *Diaporthe arecae* species complex using Euclidean distance and UPGMA algorithm. (**A**). Based on the length-to-width ratio (L/W) of alpha conidia. (**B**). Based on the L/W ratio of beta conidia. (**C**). Based on the L/W ratios of alpha and beta conidia. The species or strains included in each analysis were those with available measurements for the micromorphological structure(s) used to infer the respective dendrogram. The horizontal dashed lines indicate the distance cut-of level used to produce clusters. Euclidean distance values greater than 0.00 are shown at the nodes. Clusters are highlighted by colored lines and referred to in the chart legend. The number (#) of taxa in each cluster is noted in brackets. The cophenetic correlation coefficients (c) are noted below the chart legend.

Three dendrograms were constructed using hierarchical cluster analysis based on published taxonomic descriptions of species belonging to the DASC (Figure 8). All dendrograms presented cophenetic correlation coefficient (c) values greater than 0.75, revealing that the clustering obtained is reliable and well fit. The phenotypic data used to construct the dendrograms were the alpha and beta conidia dimensions. Since some of the species with published taxonomic descriptions do not have alpha or beta conidia, the species included in the analyses were those with available dimensions for the respective feature in which a given dendrogram is based, which should be considered when comparing the dendrograms.

While the dendrogram based on the length-to-width (L/W) ratios of alpha conidia yielded five clusters comprising one to sixteen taxa (Figure 8A), the dendrograms based on L/W of beta conidia yielded three clusters comprising six to fourteen taxa (Figure 8B). Moreover, comparing both dendrograms, clustering patterns are highly discordant and L/W ratios of alpha and beta conidia seem to differently discriminate species in the DASC.

The dendrogram based on the L/W ratio of beta conidia (Figure 8B) was highly congruent with the combined dendrogram based on the L/W ratio of alpha and beta conidia, which also yielded three clusters comprising six to twelve taxa (Figure 8C).

Therefore, dimensions of beta conidia appear to discriminate more strongly between taxa within the DASC, as none of the clusters formed based on the L/W ratio of alpha conidia (Figure 8A) were recovered when both conidia were used to group taxa. However, none of the dendrograms obtained were congruent with the previous analyses based on molecular approaches. While phylogenetic-based analyses showed that the DASC include three putative phylogenetic species (*D. arecae*, *D. chiangmaiensis* and *D. smilacicola*), the hierarchical cluster analyses did not discriminate *D. chiangmaiensis* (cluster 2 in Figure 8A) and *D. smilacicola* (cluster 5 in Figure 8A) from other taxa belonging to the *D. arecae* subclade.

## 4. Taxonomy

The present study combined phylogenetic analyses, coalescent-based models (PTP and mPTP), phylogenetic networks, recombination and population genetic diversity analyses and hierarchical cluster analysis of phenotypic data to determine the species boundaries in the *D. arecae* species complex. According to the aforementioned analyses, three sister species (*D. arecae*, *D. chiangmaiensis* and *D. smilacicola*) have been delimited in the DASC. All species previously described in the *D. arecae* lineage were shown to be conspecific, rather than different taxa. Fifty-two species are thus reduced to synonymy under *D. arecae* and morphological descriptions of the *D. arecae* isolates from foliar lesions of palms are provided. Moreover, a synopsis of the morphological data available for the species synonymized here is provided in Table 5, and the host and country, along with the ecological group of all type specimens proposed as synonyms in the present study, are summarized in Table 6.

**Figure 9:**
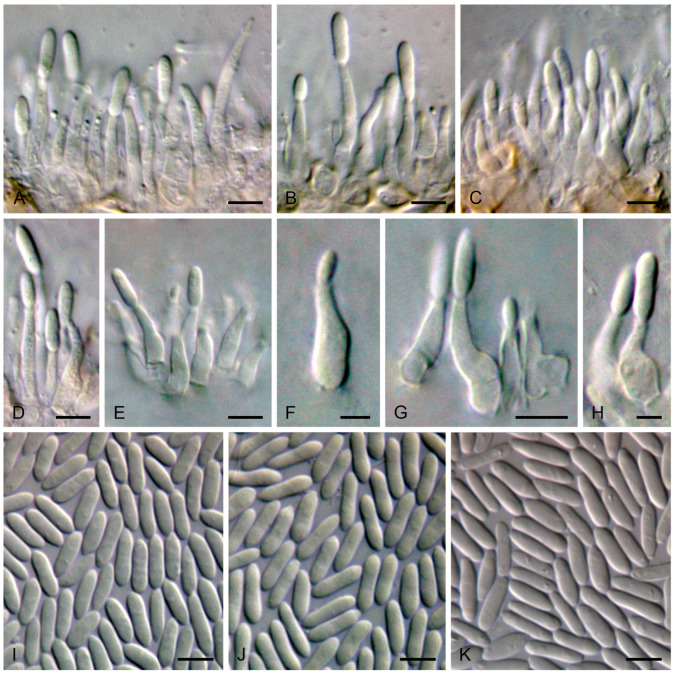
*Diaporthe arecae*. (**A**–**I**): CDP 0358; (**J**): CDP 0047; (**K**): CDP 0460. (**A**–**C**). Conidiogenous layer. (**D**–**H**). Conidiogenous cells. (**I**–**K**). Conidia. Scale bars: (**A**–**E**,**G**,**I**–**K**) = 5 μm, (**F**,**H**) = 2.5 μm.

***Diaporthe arecae*** (H.C. Srivast., Zakia & Govindar.) R.R. Gomes, Glienke & Crous, *Persoonia* 31: 16 (2013), MycoBank MB802924 (Figure 9).

*Basionym*: *Subramanella arecae* H.C. Srivast., Zakia & Govindar., *Mycologia* 54: 7 (1962), MycoBank MB339830

= *Diaporthe acuta* Y.S. Guo & G.P. Wang, *Persoonia* 45: 140 (2020), MycoBank MB830655

= *Diaporthe anhuiensis* H. Zhou & C.L. Hou, *Phytotaxa* 422: 165 (2019), MycoBank MB832081

= *Diaporthe annellsiae* Y.P. Tan & R.G. Shivas, *Index of Australian Fungi* 2: 1 (2022), MycoBank MB559559

= *Diaporthe arengae* R.R. Gomes, Glienke & Crous, *Persoonia* 31: 16 (2013), MycoBank MB802925

= *Diaporthe averrhoae* (C.Q. Chang, Z.D. Jiang & P.K. Chi) Y.H. Gao & L. Cai, *IMA Fungus* 8: 183 (2017), MycoBank MB821437

≡ *Phomopsis averrhoae* C.Q. Chang, Z.D. Jiang & P.K. Chi, *Mycosystema* 24: 6 (2005), MycoBank MB344467

= *Diaporthe bounty* Y.P. Tan & R.G. Shivas, *Index of Australian Fungi* 2: 3 (2022), MycoBank MB559562

= *Diaporthe camelliae-oleiferae* Q. Yang, *MycoKeys* 84: 22 (2021), MycoBank MB840451

= *Diaporthe ceratozamiae* Crous & R.G. Shivas, *Persoonia* 27: 133 (2011), MycoBank MB560695

= *Diaporthe cercidis* C.M. Tian & Q. Yang, *MycoKeys* 39: 124 (2018), MycoBank MB824707

= *Diaporthe chamaeropicola* D.S. Pereira & A.J.L. Phillips, *Fungal Diversity* 111: 166 (2021), MycoBank MB557847

= *Diaporthe chrysalidocarpi* S.T. Huang, J.W. Xia, W.X. Sun & X.G. Zhang, *MycoKeys* 78: 59 (2021), MycoBank MB837812

= *Diaporthe delonicis* R.H. Perera, E.B.G. Jones & K.D. Hyde, *Mycosphere* 11: 2129 (2020), MycoBank MB556855

= *Diaporthe drenthii* Y.P. Tan, Akinsanmi & R.G. Shivas, *Plant Pathology* 69: 916 (2020), MycoBank MB833828

= *Diaporthe endocitricola* Z.Y. Dong, M. Luo, M.M. Xiang & K.D. Hyde, *Frontiers in Microbiology* 11: 9 (2021), MycoBank MB557628

= *Diaporthe fraxini-angustifoliae* R.G. Shivas, J. Edwards & Y.P. Tan, *Fungal Diversity* 61: 255 (2013), MycoBank MB802384

= *Diaporthe fulvicolor* Y.S. Guo & G.P. Wang, *Persoonia* 45: 146 (2020), MycoBank MB830657

= *Diaporthe gossiae* Y.P. Tan & R.G. Shivas, *Index of Australian Fungi* 2: 5 (2022), MycoBank MB559565

= *Diaporthe guangxiensis* Dissanayake, X.H. Li & K.D. Hyde, *Frontiers in Microbiology* 10: 14 (2019), MycoBank MB552578

= *Diaporthe hongheensis* E.F. Yang & Tibpromma, *Journal of Fungi* 8: 15 (2022), MycoBank MB559411

= *Diaporthe howardiae* Y.P. Tan & R.G. Shivas, *Index of Australian Fungi* 2: 6 (2022), MycoBank MB559570

= *Diaporthe huangshanensis* H. Zhou & C.L. Hou, *Phytotaxa* 422: 169 (2019), MycoBank MB832082

= *Diaporthe hunanensis* Q. Yang, *MycoKeys* 84: 26 (2021), MycoBank MB840452

= *Diaporthe krabiensis* (Dayarathne) M.S. Calabon & E.B.G. Jones, *Botanica Marina* 66: 219 (2023), MycoBank MB848522

≡ *Diaporthe krabiensis* Dayarathne, *Mycosphere* 11: 92 (2020), MycoBank MB635831

= *Diaporthe limonicola* Guarnaccia & Crous, *IMA Fungus* 8: 328 (2017), MycoBank MB821731

= *Diaporthe liquidambaris* (C.Q. Chang, Z.D. Jiang & P.K. Chi) Udayanga & Castl., *IMA Fungus* 7: 291 (2016), MycoBank MB819021

≡ *Phomopsis liquidambaris* C.Q. Chang, Z.D. Jiang & P.K. Chi, *Mycosystema* 24: 9 (2005), MycoBank MB344462

≡ *Diaporthe liquidambaris* (C.Q. Chang, Z.D. Jiang & P.K. Chi) Y.H. Gao & L. Cai, *IMA Fungus* 8: 183 (2017), MycoBank MB821446

= *Diaporthe litchiicola* R.G. Shivas, K.R.E. Grice & Y.P. Tan [as “*litchicola*”], *Fungal Diversity* 61: 256 (2013), MycoBank MB545033

= *Diaporthe loropetali* (C.Q. Chang, Z.D. Jiang & P.K. Chi) Y.H. Gao & L. Cai, *IMA Fungus* 8: 183 (2017), MycoBank MB821448

≡ *Phomopsis loropetali* C.Q. Chang, Z.D. Jiang & P.K. Chi, *Mycosystema* 24: 148 (2005), MycoBank MB344460

= *Diaporthe meliae* C.M. Tian & Qin Yang, *MycoKeys* 91: 38 (2022), MycoBank MB829523

= *Diaporthe melitensis* Guarnaccia & Crous, *IMA Fungus* 8: 329 (2017), MycoBank MB821732

= *Diaporthe millettiae* H. Long, K.D. Hyde & Yong Wang bis, *MycoKeys* 57: 119 (2019), MycoBank MB829563

= *Diaporthe musigena* Crous & R.G. Shivas, *Persoonia* 26: 119 (2011), MycoBank MB560160

= *Diaporthe nelumbonis* Sawada ex R. Kirschner, *Mycological Progress* 17: 280 (2017), MycoBank MB821926

≡ Phyllosticta nelumbonis Sawada, Special Publication College of Agriculture National Taiwan University 8: 140 (1959), MycoBank MB336860

= *Diaporthe norfolkensis* Y.P. Tan & R.G. Shivas, *Index of Australian Fungi* 2: 8 (2022), MycoBank MB559574

= *Diaporthe oculi* Mochiz. & Kaz. Tanaka, *Journal of Infection and Chemotherapy* 25: 98 (2018), MycoBank MB825540

= *Diaporthe osmanthi* H. Long, K.D. Hyde & Yong Wang bis, *MycoKeys* 57: 120 (2019), MycoBank MB829564

= *Diaporthe pandanicola* Tibpromma & K.D. Hyde, *MycoKeys* 33: 44 (2018), MycoBank MB823840

= *Diaporthe pascoei* R.G. Shivas, J. Edwards & Y.P. Tan, *Fungal Diversity* 61: 258 (2013), MycoBank MB802387

= *Diaporthe pescicola* Dissanayake, J.Y. Yan, X.H. Li & K.D. Hyde, *Mycosphere* 8 (5): 542 (2017), MycoBank MB551988

= *Diaporthe phyllanthicola* (C.Q. Chang, Z.D. Jiang & P.K. Chi) Y.H. Gao & L. Cai, *IMA Fungus* 8: 184 (2017), MycoBank MB821461

≡ *Phomopsis phyllanthicola* C.Q. Chang, Z.D. Jiang & P.K. Chi, *Mycosystema* 24: 10 (2005), MycoBank MB344466

= *Diaporthe podocarpi-macrophylli* Y.H. Gao & L. Cai, *IMA Fungus* 8: 176 (2017), MycoBank MB820682

= *Diaporthe pseudomangiferae* R.R. Gomes, Glienke & Crous, *Persoonia* 31: 30 (2013), MycoBank MB802945

= *Diaporthe pseudooculi* Mochiz. & Kaz. Tanaka, *Journal of Infection and Chemotherapy* 25: 100 (2018), MycoBank MB825541

= *Diaporthe pseudophoenicicola* R.R. Gomes, Glienke & Crous, *Persoonia* 31: 30 (2013), MycoBank MB803839

= *Diaporthe pterocarpicola* Udayanga, Xing Z. Liu & K.D. Hyde, *Cryptogamie*, Mycologie 33: 303 (2012), MycoBank MB801053

= *Diaporthe schimae* C.M. Tian & Q. Yang, *MycoKeys* 77: 55 (2021), MycoBank MB829526

= *Diaporthe searlei* R.G. Shivas, Akinsanmi & Y.P. Tan, *Plant Pathology* 69: 918 (2020), MycoBank MB833830

= *Diaporthe sennae* C.M. Tian & Qin Yang, *Phytotaxa* 302: 149 (2017), MycoBank MB820452

= *Diaporthe spinosa* Y.S. Guo & G.P. Wang, *Persoonia* 45: 154 (2020), MycoBank MB830659

= *Diaporthe taiwanensis* H.A. Ariyaw. & I. Tsai, *Phytotaxa* 461: 161 (2020), MycoBank MB835116

= *Diaporthe taoicola* Dissanayake, J.Y. Yan, X.H. Li & K.D. Hyde, *Mycosphere* 8: 543 (2017), MycoBank MB551989

= *Diaporthe viciae* W.S. Zhao, Q. Ning & J.Y. Yan, *Mycosphere* 14: 34 (2023), MycoBank MB558423

= *Diaporthe viniferae* Dissanayake, X.H. Li & K.D. Hyde, *Frontiers in Microbiology* 10: 21 (2019), MycoBank MB552002

*Type*: **INDIA**, on fruit of *Areca catechu* (*Arecaceae*), during 1958–59, H.C. Srivastava (**holotype** of *Subramanella arecae* IMI, anon. s. n., IARI, anon. s. n.). **INDIA**, on fruit of *A. catechu*, Feb 1964, H.C. Srivastava (**isotype** of *S. arecae* CBS H-7808, ex-isotype culture CBS 161.64).

See [38] for illustrations and descriptions of asexual morph. Sexual morph was not reported for any of the specimens but was reported under the species names *D. hongheensis* [126] and *D. spinosa* [104].

Isolate CDP 0358. **Sexual morph**: Undetermined. **Asexual morph**: Conidiomata on palm leaflets in culture pycnidial, globose to subglobose, non-stromatic, uniloculate, black, solitary, occasionally aggregated in small groups, immersed in the host becoming erumpent through the ostiolar region, occasionally superficial, exuding a yellowish mucoid mass or cirrus of conidia, up to 220 μm diam. *Conidiophores* reduced to conidiogenous cells. *Conidiogenous cells* lining the pycnidial cavity, hyaline, smooth- and thin-walled, discrete, determinate, cylindrical to broadly lageniform, tapering towards the apex, straight or slightly curved, aseptate, rarely 1-septate, unbranched, rarely with one branch below the septum, rarely with minute and inconspicuous collarette, enteroblastic, proliferating at the same level giving rise to periclinal thickenings, (4.99–)7.17–16.46(–22.54) × 1.73–4.43 μm, 95% confidence limits = 10.62–11.83 × 2.41–2.63 μm (mean ± SD = 11.22 ± 2.77 × 2.52 ± 0.50 μm, n = 80). *Alpha conidia* fusoid to ellipsoid, tapering towards both ends, acute to subacute base, often slightly subtruncate with a flattened hilum, subobtuse to obtuse apex, often narrower in the middle, smooth- and thin-walled, hyaline, aseptate, eguttulate, often with granular contents, 5.76–8.88(–11.52) × 1.62–3.08 μm, 95% confidence limits = 7.26–7.50 × 2.20–2.26 μm (mean ± SD = 7.38 ± 0.75 × 2.23 ± 0.20 μm), mean ± SD conidium length/width ratio = 3.33 ± 0.40 (n = 150). *Beta* and *gamma conidia* not observed.

*Culture characteristics*: Colonies on 1/2 PDA, reaching 55 mm diameter after 7 days at 20 °C in darkness. Surface flat, with filiform margin, circular shape, whitish to pale, opaque. Reverse pale to yellowish orange. No diffusible pigment. Conidiomata black, formed in poorly defined concentric rings after about 2 weeks.

*Material examined*: **PORTUGAL**, Lisbon, Parque das Nações, Jardins da Água, Pomar do Mediterrâneo, on foliar lesions of segments of *Chamaerops humilis* (*Arecaceae*), 16 October 2018, Diana S. Pereira (specimen HDP 039), living culture CDP 0047 (*cal* sequence MT011065, ITS sequence MT002357, *tef1* sequence MT011069, *tub2* sequence MT011075); Parque das Nações, Jardins da Água, near Oceanário de Lisboa, on foliar lesions of segments of *C. humilis* (*Arecaceae*), 16 October 2018, Diana S. Pereira (specimen HDP 034), living culture CDP 0460 (ex-type culture of *D. chamaeropicola*, **holotype** AVE-F-8) (*cal* sequence MT011068, ITS sequence MT022111, *tef1* sequence MT011074, *tub2* sequence MT011080); Parque das Nações, on foliar lesions of leaflets of *Phoenix dactylifera* (*Arecaceae*), 16 October 2018, Diana S. Pereira (specimen HDP 044), living culture CDP 0358 (*cal* sequence MT011067, ITS sequence MT004743, *tef1* sequence MT011073, *tub2* sequence MT011079).

*Hosts*: Reported from more than 45 genera and 50 species in 32 families, including *Altingiaceae* (*Liquidambar formosana*), *Anacardiaceae* (*Mangifera indica*), *Arecaceae* (*Areca catechu*, *Arenga engleri*, *Chamaerops humilis*, *Chrysalidocarpus lutescens*, *Phoenix canariensis*, *P. dactylifera*), *Asparagaceae* (*Agave* sp.), *Betulaceae* (*Corylus avellana*), *Cannabaceae* (*Celtis formosana*), *Convolvulaceae* (*Ipomoea batatas*), *Cupressaceae* (*Cunninghamia lanceolata*), *Euphorbiaceae* (*Hevea brasiliensis*), *Fabaceae* (*Cercis chinensis*, *Delonix regia*, *Millettia reticulata*, *Pongamia pinnata*, *Pterocarpus indicus*, *Senna bicapsularis*, *Sesbania* sp., *Vicia villosa*), *Ginkgoaceae* (*Ginkgo biloba*), *Hamamelidaceae* (*Loropetalum chinense*), *Lauraceae* (*Persea americana*, *P. gratissima*), *Meliaceae* (*Melia azedarach*), *Moraceae* (*Ficus ampelos*), *Musaceae* (*Musa* sp.), *Nelumbonaceae* (*Nelumbo nucifera*), *Oleaceae* (*Fraxinus angustifolia*, *Olea europaea*, *Osmanthus fragrans*), *Oxalidaceae* (*Averrhoa carambola*), *Pandanaceae* (*Pandanus* sp.), *Phyllanthaceae* (*Phyllanthus emblica*), *Poaceae* (*Dendrocalamus latiflorus*), *Podocarpaceae* (*Podocarpus macrophyllus*), *Proteaceae* (*Macadamia* sp.), *Rhizophoraceae* (*Bruguiera* sp.), *Rosaceae* (*Malus domestica*, *Prunus persica*, *Pyrus bretschneideri*, *P. communis*, *P. pyrifolia*), *Rubiaceae* (*Ixora chinensis*), *Rutaceae* (*Citrus grandis*, *C. limon*, *C. reticulata*, *C. sinensis*, *Citrus* sp., *C. unshiu*), *Sapindaceae* (*Acer palmatum*, *A. Pictum*, *Litchi chinensis*), *Theaceae* (*Camellia oleifera*, *Schima superba*), *Vitaceae* (*Vitis vinifera*) and *Zamiaceae* (*Ceratozamia robusta*) ([128], present study).

*Distribution*: Australia (including the Norfolk Island), Caucasia, China, Dominican Republic, Hong Kong, India, Iran, Iraq, Italy, Japan, Malaysia, Malta, Mexico, Netherlands, Portugal, Puerto Rico, South Africa (including KwaZulu-Natal and Mpumalanga provinces), Spain, Suriname, Taiwan, Thailand, Turkey, USA ([128], present study).

*Notes*: *Diaporthe arecae* was introduced by Srivastava et al. [38] as *Subramanella arecae* from *Areca catechu* in India and was later assigned to *Diaporthe* by Gomes et al. [24]. Several studies have revealed that most loci used to infer the phylogeny of *Diaporthe* species failed to resolve the phylogenetic position of *D. arecae* and its related species, insomuch that the clade has been treated as a species complex [35]. Over the years, more than 50 species from various hosts distributed worldwide have been introduced to the *D. arecae* species complex (DASC) (Table 5 and Table 6). The integrative taxonomic approach conducted in this study revealed that all “species” introduced in the *D. arecae* subclade represent intraspecific variation and were therefore synonymized under *D. arecae*. According to the analyses conducted here, the strains “*D. eugeniae*” CBS 444.82 and “*D. perseae*” CBS 151.73 were shown to be synonyms of *D. arecae*. However, the species *D. eugeniae* and *D. perseae* were not considered in the synonyms proposed here, since no type strains have been formally linked to these species. *Diaporthe eugeniae* (as *Phomopsis eugeniae*) was originally described on *Eugenia aromatica* from West Sumatra, Indonesia [129]. Later, Gomes et al. [24] analyzed the strain CBS 444.82 from *E. aromatica* in Lampung, Indonesia and considered this isolate to be authentic for *D. eugeniae*, but no epitype was formally designated since the isolate proved to be sterile. *Diaporthe perseae* (as *P. perseae*) was originally described from branches of dying *Persea gratissima* trees in Russia [130]. Later, Gomes et al. [24] analyzed the strain CBS 151.73 from young a fruit of *P. gratissima* in the Netherlands Antilles and considered this strain to be authentic to *D. perseae* based on the morphology of its alpha conidia, but no epitype was formally designated. As no ex-type cultures exist either for *D. eugeniae* or *D. perseae*, the strains “*D. eugeniae*” CBS 444.82 and “*D. perseae*” CBS 151.73 were here assigned to *D. arecae*. In spite of this, since neither of these two strains are linked to the holotypes, the species epithets *eugeniae* and *perseae* could not be made synonyms of *D. arecae*. Although it is clear through the analyses conducted here that all “species” in the *D. arecae* subclade are conspecific; internal nodes and sub-branches were observed in this subclade, indicating the possibility of active divergence and speciation. Morphologically speaking, all “species” harbor fusoid to ellipsoid alpha conidia and filiform, curved to hamate beta conidia of considerably overlapping dimensions, a common absence of gamma conidia (observed only in *D. limonicola*, *D. musigena* and “*D. perseae*”), as well as conidiomata, conidiophores and/or conidiogenous cells that lie within the same size ranges (Table 5). Considering the morphological data available for the “species” synonymized here, the mean dimensions of the alpha and beta conidia produced by *D. arecae* strains are 6.07–8.49 × 1.93–2.7 μm (mean L/W = 1.96–4.60) and 18.60–29.14 × 1.02–1.53 μm (mean L/W = 10.25–30.00), respectively, which clearly overlap the dimensions reported for the type specimen of *D. arecae* (CBS H-7808; alpha and beta conidia dimensions = 7.2–9.6 × 2.4 μm and 14.4–24 × 1.2 μm, respectively) (Table 5). Thus, except for the production of gamma conidia observed in the aforementioned “species”, the morphology of the asexual morph of all *D. arecae* strains match the original description reported by Srivastava et al. [38]. The three isolates from foliar lesions of palms in Lisbon, Portugal (CDP 0047, CDP 0358 and CDP 0460) are also morphologically similar to the type specimen of *D. arecae* [38] (Figure 9). Considering the strain characterized here (CDP 0358) and the type specimen of *D. arecae* (CBS H-7808), both produce hyaline, aseptate and ellipsoid alpha conidia of overlapping dimensions (5.76–8.88 × 1.62–3.08 μm and 7.2–9.6 × 2.4 μm, respectively) [38]. Nevertheless, the production of beta conidia has not been observed for any of the strains characterized in the present study, as already reported for other “species” introduced in the *D. arecae* subclade. The morphological differences observed among the *D. arecae* strains fit in well with the extensive plasticity that the *Diaporthe* genus is known to exhibit. The phenotypic plasticity of *D. arecae* has been well observed in the three isolates from foliar lesions of palms characterized in this study. While all three isolates tend to develop stromatic, uni- to multilocular, inostiolate pycnidial conidiomata of variable shape and size when grown on PDA, the pycnidia produced when grown on WA are non-stromatic, unilocular, ostiolate, globose to subglobose and much less variable in size. Interestingly, the stromatic pycnidial conidiomata observed on PDA highly resemble the pycnosclerotia described by Srivastava et al. [38] for the type of *D. arecae* specimen, which are also multiloculate and inostiolate. Moreover, while long, cylindrical, unbranched or branched paraphyses, that later often function as conidiogeneous cells, are observed in the conidiogeneous layer of all three isolates when they are grown on PDA, the pycnidia produced when grown on WA lack paraphyses. Thus, the morphological variability among taxa belonging to the *D. arecae* subclade, such as the absence or presence of paraphyses, beta- or gamma-conidia (Table 5), are likely to be a result of character plasticity due to environmental conditions. No relevant variation in micromorphology was observed between the strains from foliar lesions of palms and all three strains present very similar alpha conidial dimensions and remarkably similar alpha conidia L/W ratios (mean = 8.24 × 2.38 μm, L/W = 3.49 for CDP 0047, 7.38 × 2.23, L/W = 3.33 for CDP 0358 and 7.53 × 2.31 µm, L/W = 3.33 for CDP 0460). *Diaporthe arecae* has not previously been reported in Portugal, representing a new geographical record. Moreover, this is the first time this species has been recorded on *Chamaerops humilis*, representing a new host record. The isolates of *D. arecae* studied here were recorded from foliar lesions of palms, but their pathogenicity has not been tested. However, *D. arecae* has been introduced as causing the severe post-harvest fruit rot of *A. catechu* [38] and has already been recorded on leaf spots of *A. catechu* [65] and *Chrysalidocarpus lutescens* [110]. Other palm tree species known to be hosts of *D. arecae* include *Arenga engleri* [24], *Calamus castaneus* [67], *Phoenix canariensis* [26] and *P. dactylifera* ([24], present study). Although *D. arecae* has primarily been described from palms and is frequently reported on *Arecaceae* hosts, the geo–ecological data for the isolates recognized here as *D. arecae* suggests that this species has a widespread distribution and a broad host range as a pathogen, endophyte or saprobe, e.g., refs. [21,104,105,107,114,124] (Table 6).

## 5. Discussion

Given the overlap in morphological features, coupled with morphological plasticity, Phylogenetic Species Recognition (PSR) has become the standard methodology for the identification of species in *Diaporthe* [13,23,24,25,26,27]. However, most *Diaporthe* spp. have been introduced in recent years as well-supported terminal clades based on gene concatenation, without looking for incongruences between individual gene trees or evaluating the lack of gene flow between populations. Therefore, a spurious proliferation in the number of *Diaporthe* species has been observed. This is largely attributed to the intraspecific variability of the genus, that hinders the interpretation of phylogenetic analyses and has been erroneously used to delimit species [13,23,24,26]. In this regard, following a survey of leaf-spotting fungi associated with palm trees in Lisbon, Portugal, the present study aimed to clarify the boundaries of species within the *Diaporthe arecae* species complex (DASC) by implementing an integrative taxonomic approach. Three species—*D. arecae*, *D. chiangmaiensis* and *D. smilacicola*—have been recognized in the complex, and fifty-two previously introduced species were shown to be synonyms of *D*. *arecae*. To the best of authors’ knowledge, this is the first study to establish a robust circumscription of species in the DASC.

It has long been argued that species circumscription should be based on the simultaneous and rigorous application of multilocus analyses and genealogical concordance [44,131]. Genealogical Concordance Phylogenetic Species Recognition (GCPSR) has been shown to have profound implications for accurate species recognition, and resolution of complexes of cryptic taxa [78,132,133] and has already been successfully applied to resolve cryptic species of common phytopathogenic genera, such as *Armillaria* [134], *Fusarium* [135,136], *Plagiostoma* [137], *Phyllosticta* [138], *Colletotrichum* [139,140,141] and *Calonectria* [142], as well as *Diaporthe* [9,23,36,37]. In the present study, phylogenetic analyses of combined datasets revealed some well-supported clades within the DASC, previously interpreted as different species. However, most of the taxa composing these clades showed phylogenetic discordance in the individual phylograms, revealing incongruent nodes, conflicting branches, a lack of phylogenetic support and frequently displayed a polyphyletic or paraphyletic nature in some individual phylograms. Moreover, genealogical concordance and genealogical non-discordance criteria indicated that the node delimiting the DASC represents the transition from concordant to incongruent branches and three independent evolutionary lineages (IEL) were recognized within the DASC as mentioned above. The incongruences observed between individual gene genealogies suggest that the loci used for phylogenetic inferences of the DASC may harbor different evolutionary histories [46,47,50]. A similar result has also been inferred from the incongruence length difference (ILD) tests performed, which indicated that the loci were not congruent and should be analyzed separately [143]. Therefore, the concatenation of different loci for phylogenetic inferences within the DASC is an inadequate approach, as it tends to overestimate species diversity. Moreover, the conflict observed among gene trees can be reasonably explained by recombination events among individuals within a species, which in turn may indicate a lack of reproductive isolation [144,145,146,147]. Hence, given the extensive incongruent lineages observed among taxa within the *D. arecae* subclade, the GCPSR principle indicates that they are conspecific, representing a single IEL. Accordingly, it is suggested that 52 species previously described in the *D. arecae* subclade represent intraspecific variability, which is supported by the population genetic diversity analyses.

The degree of genetic diversity within the DASC revealed a high haplotype diversity above 95% and a substantial low nucleotide diversity for all loci and combined datasets. This is indicative of a high number of haplotypes that differ by only small differences that may be due to new polymorphisms [148]. As described by Grant and Bowen [149], the combination of high haplotype diversity and low nucleotide diversity can be a signature of a rapid demographic expansion from a small effective population size that enhances the retention of new mutations. Thus, it is hypothesized here that the DASC might be under a recent population expansion, which is consistent with the large number of unique haplotypes and polymorphic sites found in all loci and combined datasets. Further evidence for an excess of new mutations concomitant with recent population size expansion was suggested by the negative values of Tajima’s D neutrality test [150]. While positive significant Tajima’s D values are indicative of a balancing selection, where the absence of significant recombination maintains advantageous genetic diversity, negative significant Tajima’s D values suggest an excess of rare alleles in the population that have arisen after the fixation of a new beneficial genetic variant [97,151]. Thus, the present results suggest that the DASC may have escaped from an equilibrium model of evolution, which can be explained by recombination events, occurring mainly in ITS and *tef1* loci. This hypothesis was also corroborated by the topology of the phylogenetic networks built for the DASC.

Phylogenetic networks are a generalization of phylogenetic trees, used to display more complex evolutionary histories. They allow the representation of non-treelike evolutionary events (reticulations), such as recombination, hybridization and horizontal gene transfer, and thus, can be interpreted as a visualization of contradictory phylogenetic information [90,152,153]. The phylogenetic networks of the DASC were composed by parallel edges and boxlike polygons among virtually all taxa belonging to the *D. arecae* subclade, a characteristic of the presence of recombination events within the dataset. Thus, the present results suggest that the DASC is a population that may have undergone a recent expansion, which is mainly related to the *D. arecae* subclade that is a single entity producing a large number of offspring [148,154]. Recombination creates new genotypes by combining genetic material from distinct lineages, and in turn, enhances the population genetic diversity [155,156]. The recombination events among some taxa of the *D. arecae* subclade may have led to recently diverged individuals within the DASC that retained ancestral polymorphisms, as suggested by the presence of a high number of closely related haplotypes, evidencing a population under incomplete lineage sorting (ILS) [47,63]. The formulated hypotheses are in line with the existence of extensive phylogenetic incongruences between gene trees among taxa within the *D. arecae* subclade. Therefore, the *D. arecae* subclade should be considered as ongoing evolving lineages since the internal nodes and sub-branches indicate the possibility of active divergence and speciation.

Considering that gene concatenation was found to be unsuitable for species circumscription within the DASC, the above-mentioned formulated hypotheses were tested through the application of the coalescent-based methods single- and multi-rate Poisson Tree Processes (PTP and mPTP, respectively). Coalescent-based models are an efficient tool for studying the evolutionary processes that contribute to speciation, since they can infer the relationships among taxa and delimit IEL objectively even in the presence of gene–tree conflict [60]. PTP and similar coalescent-based methods use the distinct branching patterns between divergence (Poisson model) and intraspecific diversification (coalescent model) to distinguish between speciation and population processes, which is measured in terms of the number of nucleotide substitutions per site [86]. The main assumption of these methods is that within-species branching events will be substantially more frequent than between species and thus the transition between different branching patterns is the threshold used to predict species boundaries [157]. Recent studies have successfully applied coalescent methods to delimit boundaries of cryptic species complexes of fungi, where there is a dearth of distinctive morphological characters. For instance, Liu et al. [34] showed that the distinct lineages of *Colletotrichum siamense sensu latu* recognized as different species based on gene concatenation were recognized as a single species when applying coalescent methods. Similarly, coalescent methods have been successfully used in the identification of the number of species in the *Alternaria alternata* [47] and *Fusarium oxysporum* [48] species complexes. In the present study, both PTP and mPTP recognized three species within the DASC—*D. arecae*, *D. chiangmaiensis* and *D. smilacicola*—as suggested by the GCPSR principle. Moreover, both methods inferred that the *D. arecae* subclade should be recognized as a single species, concordant with the results suggested by population genetic diversity analyses. Thus, overestimated species in the *D. arecae* subclade, obtained in the concatenated multilocus analyses, were not supported by coalescent-based analyses. A few recent studies have also applied coalescent models to resolve other important species complexes in *Diaporthe*. Hilário et al. [36,37] applied the General Mixed Yule Coalescent (GMYC) and PTP models to reliably delimit the boundaries of *D. amygdali* and *D. eres*, which drastically reduced the number of taxa that were previously recognized as different lineages related to both species.

Phylogenetic informativeness (PI) profiles were generated to compare each locus with respect to the species hypothesis inferred based on the multilocus phylogenetic analyses. Previous studies have shown that *tef1* is the most informative locus out of the five common loci used for molecular identification within the *Diaporthe* genus [7,9,23,30,32]. However, although *tef1* locus showed the highest number of informative characters to resolve the DASC, the pairwise homoplasy index (PHI) test revealed significant intragenic recombination, and the Tajima’s D test gave significant negative values, which can be also indicative of recombination events within the population at that locus. Moreover, the individual phylograms of the *cal* and *his3* loci were more congruent with the backbone structure of the three well-supported subclades within the DASC observed in all the multilocus analyses and predicted by the GCPSR principle. In addition, PI profiles ranked *cal* as the most phylogenetic informative locus to infer the species limits of the DASC. Thus, the present study suggests that the definition of the optimal set of loci that can be used for species identification in *Diaporthe* may depend on the clade under analysis. For the DASC, the *cal* locus seems to be the most appropriate locus to infer species limits, although the evolutionary relationships among taxa become better resolved and supported when all five loci are simultaneously used for phylogenetic inferences, as corroborated by previous studies, e.g., ref. [32].

Integrating PI over specific periods of time provides information for ranking loci, since the integration area will be largest for the loci that have the highest probability of substitution in the given time period [80,81]. An interesting pattern was observed in the PI profile of ITS. ITS showed the lowest informative characters to resolve the DASC, suggesting that this locus might not be suitable for species delimitation within the DASC, as already suggested for other *Diaporthe* species complexes [7,9,36]. Nonetheless, while ITS is the least informative locus as the tree approaches its root, a substantial peak in the PI profile of ITS corresponding to the specific relative period of time in which the *D. arecae* subclade radiates into several branches. This indicates that ITS ranks as the most informative marker to infer intraspecific variation within the DASC. Although ITS has been widely used in fungal systematics to delimit species and to understand evolutionary relationships [158,159], several known issues related to the effectiveness of this region have already been observed, including the overestimation and underestimation of fungal diversity [30,160,161,162]. Several studies have shown ITS to be uninformative for accurate species identification in *Diaporthe* due to the lack of interspecific variation [1,8,30,43], which has also been observed in the present study. Nonetheless, it might be a suitable locus to test evolutionary hypotheses, such as the occurrence of recombination between strains.

PI plots quantify and display a predicted signal without accounting for phylogenetic noise. Hence, the results presented here should be considered carefully in the light of homoplasy, which is likely to rise or diminish the utility of loci during certain periods of time different from the peak informativeness for a given profile [80,81]. A high degree of homoplasy has been detected among ITS sequences within the DASC. Homoplasy may arise from reticulation events during the evolutionary history and, as a result, can be seen as an indirect measure of recombination [163] shown to be statistically significant among ITS sequences. Thus, the ITS peak observed in the PI profiling are likely to be influenced by the presence of homoplasy among the ITS sequences.

Morphology, as well as ecological traits, are also used to delimit species of fungi. However, species defined based on morphology or ecology often comprise cryptic species when the PSR is applied [44,164,165,166]. In this regard, the formulated hypothesis, of three putative species within the DASC, was also tentatively tested for both the morphological and ecological traits of all taxa belonging to the DASC. For many years, taxonomic studies in *Diaporthe* have been primarily based on Morphological Species Recognition (MSR), according to which, species in the *Diaporthe* were diagnosed by a set of morphological characters [167,168]. However, MSR was shown to be unreliable in reflecting the evolutionary history of the genus, as morphological characters within the *Diaporthe* are highly conserved and display great plasticity depending on environmental conditions [14]. Similarly, in the present study, based on published taxonomic descriptions of the species belonging to the DASC, it was evident that they present morphological indistinctiveness. Due to the subjectivity of characterizing some morphological structures, a hierarchical cluster analysis (HCA) was performed based on the length-to-width (L/W) ratios of alpha and beta conidia, which are discrete and easily identifiable structures whose characterization is naturally subject to greater objectivity. Although the dendrograms of the L/W conidial ratios yielded three to five different clusters of species within the DASC, according to the conidia used in the HCA, they did not support any of the clades or subclades observed in the combined and individual phylograms. Moreover, morphological characters did not discriminate between the three species delimited within the DASC. Thus, morphological characters are not reliable in delimiting species within the DASC, which showed cryptic speciation when the L/W ratios of alpha conidia were compared. Likewise, the differences detected between the L/W ratios of alpha and beta conidia of taxa belonging to the *D. arecae* subclade are simply a reflection of the intraspecific variability and character plasticity of *D. arecae*. It is worth mentioning that using standardized media and growth conditions can probably result in more stable and reliable morphological characters for diagnosis coupled with molecular data for the species recognized within the DASC, as already suggested by Mostert et al. [14]. For instance, it has already been shown that temperatures above 30 °C or a dextrose concentration seems to influence the production of beta conidia in certain *Diaporthe* species [14]. Likewise, although a higher variability in the L/W of beta conidia was observed, it is more likely that it represents character plasticity than morphospecies within the DASC.

Besides morphological characters, host plants have also been extensively used in the past as a key feature in the identification of species in *Diaporthe*. Nonetheless, studies have long shown that one *Diaporthe* species colonizes more than one host species, and that host switching appears to have occurred frequently during speciation [11,18,169,170]. These observations were confirmed by the results obtained in the present study, since taxa belonging to the *D. arecae* subclade were introduced based on collections from several different plant hosts belonging to 25 different plant families, and two species (*D. oculi* and *D. pseudooculi*) were found to be associated with diseased human eyes [42]. Considering that the Ecological Species Recognition (ESR) diagnoses different species as a set of lineages occupying a specific ecological niche (e.g., host plant or locality), evolving separately from all other lineages [167,168], the well-supported branches recognized in a phylogenetic inference might be used as a guide to find diagnostic ecological differences between taxa belonging to these branches [34]. However, the present results also showed a clear lack of phylogeographical association among taxa belonging to the DASC, as most well-supported branches in the complex show a wide geographical distribution and are not restricted to a specific locality or host plant. The detection of significant recombination within closely related taxa should be considered as an important method to justify a species [171]. Thus, to further test the possible correlation between the genetic divergence of clades within the DASC and their ecological niche, the well-supported branches recognized in the phylogenetic inference were tested for genetic exchange to assess their evolutionary independence. According to the present results, significant genetic recombination within some branches and between some of the paired branches was detected, suggesting a lack of reproductive isolation between most species introduced in the DASC. For instance, isolates of branches b and g showed significant recombination between themselves and with isolates of all remaining branches in the phylogenetic inference, although the results are likely to be influenced by the presence of significant recombination between the branches themselves. Nevertheless, clades within which no significant recombination was detected revealed significant recombination with some other branches. Therefore, the ecological aspects of taxa within the DASC suggest an absence of host plant and/or geographic barriers to gene flow in nature, providing further evidence to support the hypotheses formulated by the phylogenetic and population genetic diversity analyses.

Although all the analyses carried out clearly showed that *D. chiangmaiensis* and *D. smilacicola* are delimited from *D. arecae*, significant recombination was detected between both species and the *D. arecae* subclade. Hence, the detection of significant recombination between these lineages may be the result of a recent speciation process, i.e., the three lineages may have radiated from a recent common ancestor, since some alleles are not expected to be reciprocally monophyletic in the initial stages of speciation [54,63]. This hypothesis is in line with the relative branch distances observed in the phylogenetic networks for *D. smilacicola* and *D. chiangmaiensis*, which appear to have just emerged from the complex reticulation of branches that constitutes the evolutionary relationships in the *D. arecae* subclade. Moreover, it is also supported by the incongruences observed in some of the single gene phylograms that do not present the backbone structure of three well-supported subclades within the DASC. Furthermore, the existence of a putative hybrid in *Diaporthe* was recently reported [43]. It is therefore worth mentioning that the three species recognized in the complex may also be linked by occasional hybridization, which would also justify the incongruences detected. However, this hypothesis could only be tested with genome-scale data and the use of a larger number of isolates of *D. smilacicola* and *D. chiangmaiensis*.

Although the analyses conducted here are clear in delimiting *D. arecae*, *D. chiangmaiensis* and *D. smilacicola* as three distinct sister lineages, virtually nothing is known about the ecology of these lineages, including their host ranges and lifestyles. The genetic diversity analyses performed here raise several questions regarding the speciation process in *Diaporthe* and how it may affect the pathology of species recognized within the DASC. The forces driving the intraspecific variation in *Diaporthe* species reported by a few authors, e.g., ref. [7] is still poorly explored. For instance, Manawasighe et al. [21] demonstrated that the genetic variation of *D. eres* associated with grapevine dieback in China were positively correlated with their geographic location. Nonetheless, the same conclusions were not obtained by Chaisiri et al. [55], who compared Chinese and European *D. eres* isolates and found no significant differences between the genetic diversity of the two geographical populations. Moreover, they found no association between the groups in the Chinese population of *D. eres* and their geographic distribution. Similarly, in the present study, there was no phylogeographic correlation between *D. arecae* isolates (*D. arecae* subclade). Therefore, it is suggested that further studies towards the genetic diversity of *D. arecae* and their country of origin, with a greater number of strains, should be conducted to better clarify if certain genotypes are associated with specific ecological niches.

Population divergence and its intraspecific genetic diversity has frequently impaired the interpretation of *Diaporthe* phylogenies and the accurate identification of *Diaporthe* spp. However, the problem of reliably identifying species in *Diaporthe* has practical consequences when studying the phylogenetic relationships in this genus due to their recurrent association with plant diseases [172]. The accurate identification and naming of fungal pathogens are essential to understand the aspects of their phytopathology, including epidemiology, disease surveillance and control, as well as plant health inspection [173,174]. In this regard, clarification of the species boundaries within the DASC significantly improves the knowledge of taxonomy and host diversity in *D. arecae* and highlights the unknown potential of this species as an important phytopathogenic agent. The great majority of *D. arecae* isolates have been reported as minor pathogens on a wide range of plant hosts, mostly associated with leaf spots [31,64,107,108,110,120,121,122,123,125], diseased branches, twigs, stems, trunks and shoots [10,21,39,41,104,117], as well as rotten plant parts [24,39,112,119]. Nonetheless, *D. arecae* (as *S. arecae*) has been introduced as a cause of severe post-harvest fruit rot of *Areca catechu* [38] and has also been reported (as *D. limonicola* and *D. melitensis*) to be associated with a devasting dieback disease of *Citrus* plants in some Europe countries [10]. Thus, the presence of certain genotypic variants of *D. arecae* in some hosts can lead to outbreaks of major infections. This is particularly relevant considering the current scenario of global climate change, due to which plant communities come under pressure which may facilitate the emergence of more aggressive *D. arecae* strains capable of colonizing new hosts [175,176]. Furthermore, changing environments may represent an opportunity for fungi to switch from an endophytic or saprophytic lifestyle to a pathogenic lifestyle [21], which would not be surprising if found in *D. arecae* as it has been recorded as pathogens, saprobes and endophytes on different plant hosts. For instance, the ability of *D. arecae* to switch from an endophytic to a pathogenic lifestyle has previously been commented on by Srivastava et al. [38] who isolated *D. arecae* from both rotten and healthy-looking fruits from arecanut, suggesting that *D. arecae* might be present in *Areca catechu* fruits as an endophyte or a latent pathogen.

The three isolates in this study were obtained from foliar lesions of ornamental palms, but their pathogenicity has not been proven. Moreover, this was the first report on *D. arecae* strains from *Chamaerops humilis* from Portugal, representing a new host and geographical record. Thus, future studies should aim to better understand the phytopathogenic potential of these isolates of *D. arecae*, especially the genotypic variant previously identified as *D. chamaeropicola* [64], due to its potential to represent a threat for certain important Portuguese crops, such as *Vitis vinifera* and *Pyrus* spp., from which minor pathogenic strains of *D. arecae* have often been isolated [21,104]. To date, *Diaporthe arecae* has been recorded on six different *Arecaceae* hosts, including *Areca catechu* (as *S. arecae*, *D. limonicola* and *D. pseudophoenicicola*) [38,65], *Arenga engleri* (as *D. arengae*) [24], *Calamus castaneus* (as *D. arengae* and *D. arecae*) [67], *Chamaerops humilis* (as D*. chamaeropicola* and *D. pseudophoenicicola*) ([64], present study), and *Chrysalidocarpus lutescens* (as *D. chrysalidocarpi*) [110], *Phoenix canariensis* and *P. dactilyfera* (as *D. pseudophoenicicola*) ([24,26,64], present study), indicating that this may be a frequent species of *Diaporthe* occurring on palms. Nonetheless, the extent of *Diaporthe* spp. associated with *Arecaceae* hosts is highly overlooked and only a few species have been studied using morphomolecular analyses, making most old records unreliable. Furthermore, the ecology of *D. arecae* on *Arecaceae* needs further research to establish its potential as a possible threat to certain palm species. Although Srivastava et al. [38] first reported *D. arecae* (as *S. arecae*) causing a severe post-harvest fruit rot of *Areca catechu*, subsequent records of *D. arecae* on *Arecaceae* hosts were associated with either symptomless or endophytic occurrences [105,111,113,114,126,127] and minor diseases, such as leaf spots ([64,110], present study).

## 6. Conclusions

Molecular analyses based on the GCPSR principle and PTP coalescent models provided strong evidence that all species previously described in the *D. arecae* subclade are conspecific. Further analyses, i.e., the PHI test and population genetic diversity, coupled with morphological indistinctiveness, have reinforced the absence of reproductive isolation, as well as host plant and geographic barriers to gene flow. Yet, additional analyses are needed to better understand the genetic diversity of *D. arecae* through the isolation of a greater number of strains, as well as to establish its phytopathogenic potential for *Arecaceae* hosts and other important crops worldwide. Our results suggest that speciation events in *Diaporthe* are highly overestimated. Previous studies have accepted well-supported clades as distinct species using phylogenetic analyses based on concatenation of multilocus DNA sequence data. However, phenotypic plasticity associated with insufficient phylogenetic resolution often misleads species identification, which is erroneously used to describe new taxa. Hence, it is here advocated that individual gene genealogies must always be checked for incongruences and carefully analyzed prior to the description of new *Diaporthe* species. Furthermore, this study has suggested that the optimal set of loci for species identification in *Diaporthe* may depend on the clade under analysis. A critical analysis of the informativeness of different loci must be carried out to clarify which of them is most likely to best infer the evolutionary relationships between taxa. In addition, upcoming studies on the *Diaporthe* genus should also implement coalescent methods to provide accurate support for multilocus phylogenies. The integrative taxonomic approach carried out here can clarify species boundaries in most clades where the use of highly polymorphic sequences for common loci hinders the clear interpretation of phylogenetic inferences. Therefore, this methodology provides a solid framework that can be applied for species delimitation in morphologically conserved fungi.

## Figures and Tables

**Table 1:** Collection details and GenBank accession numbers of taxa included in the phylogenetic analyses.

Taxon ^1^	Culture ^2^ and Status ^3^	Host	Country	GenBank Accession Number ^4^				
				ITS	*tef1*	*tub2*	*cal*	*his3*
*Diaporthe arecae*	CBS 535.75	*Citrus* sp.	Suriname	KC343033	KC343759	KC344001	KC343275	KC343517
	CBS 161.64^IT^	*Areca catechu*	India	KC343032	KC343758	KC344000	KC343274	KC343516
	SM30	*Calamus castaneus*	Malaysia	MN651492	MT077090	MT077061	N/A	N/A
*D. arecae* (“*D. eugeniae*”)	CBS 444.82	*Eugenia aromatica*	Indonesia	KC343098	KC343824	KC344066	KC343340	KC343582
*D. arecae* (“*D. perseae*”)	CBS 151.73	*Persea americana*	Netherlands	KC343173	KC343899	KC344141	KC343415	KC343657
*D. arecae* (syn. *D. acuta*)	CGMCC 3.19600^T^	*Pyrus pyrifolia*	China	MK626957	MK654802	MK691124	MK691225	MK726161
	PSCG 045	*Pyrus pyrifolia*	China	MK626956	MK654809	MK691223	MK691123	MK726160
*D. arecae* (syn. *D. anhuiensis*)	CNUCC 201901^T^	*Cunninghamia lanceolata*	China	MN219718	MN224668	MN227008	MN224549	MN224556
	CNUCC 201902^PT^	*Cunninghamia lanceolata*	China	MN219727	MN224669	MN227009	MN224550	MN224557
*D. arecae* (syn. *D. arengae*)	CBS 114979^T^	*Arenga engleri*	Hong Kong	KC343034	KC343760	KC344002	KC343276	KC343518
	SM28	*Calamus castaneus*	Malaysia	MN651480	MT077093	MT077062	N/A	N/A
	SM29	*Calamus castaneus*	Malaysia	MN651482	MT077094	MT077063	N/A	N/A
	SM35	*Calamus castaneus*	Malaysia	MN651483	MT077099	MT077068	N/A	N/A
	SM38	*Calamus castaneus*	Malaysia	MN651484	MT077097	MT077066	N/A	N/A
	SM39	*Calamus castaneus*	Malaysia	MN651485	MT077098	MT077067	N/A	N/A
	SM41	*Calamus castaneus*	Malaysia	MN651481	MT077095	MT077064	N/A	N/A
	SM45	*Calamus castaneus*	Malaysia	MN651486	MT077096	MT077065	N/A	N/A
	SM49	*Calamus castaneus*	Malaysia	MN651487	MT077089	MT077069	N/A	N/A
*D. arecae* (syn. *D. averrhoae*)	SCHM 3605^H^	*Averrhoa carambola*	China	AY618930	N/A	N/A	N/A	N/A
*D. arecae* (syn. *D. bounty*)	BRIP 59361a^H^	*Malus domestica*	Australia	OM918690	OM960599	OM960617	N/A	N/A
*D. arecae* (syn. *D. camelliae-oleiferae*)	HNZZ027^T^	*Camellia oleifera*	China	MZ509555	MZ504707	MZ504718	MZ504685	MZ504696
	HNZZ030	*Camellia oleifera*	China	MZ509556	MZ504708	MZ504719	MZ504686	MZ504697
*D. arecae* (syn. *D. ceratozamiae*)	CBS 131306^T^	*Ceratozamia robusta*	Australia	JQ044420	N/A	N/A	N/A	N/A
*D. arecae* (syn. *D. cercidis*)	CFCC 52565^T^	*Cercis chinensis*	China	MH121500	MH121542	MH121582	MH121424	MH121460
	CFCC 52566	*Cercis chinensis*	China	MH121501	MH121543	MH121583	MH121425	MH121461
*D. arecae* (syn. *D. chamaeropicola*)	CDP 0460^T^	*Chamaerops humilis*	Portugal	MT022111	MT011074	MT011080	MT011068	N/A
*D. arecae* (syn. *D. chrysalidocarpi*)	SAUCC 194.33^PT^	N/A	China	MT822561	MT855874	MT855758	MT855645	MT855530
	SAUCC 194.35^T^	N/A	China	MT822563	MT855876	MT855760	MT855646	MT855532
*D. arecae* (syn. *D. delonicis*)	MFLU 16-1059^H^	*Delonix regia*	Thailand	MT215490	N/A	MT212209	N/A	N/A
*D. arecae* (syn. *D. drenthii*)	BRIP 66523	*Macadamia* sp.	South Africa	MN708228	MN696525	MN696536	N/A	N/A
	BRIP 66524^T^	*Macadamia* sp.	South Africa	MN708229	MN696526	MN696537	N/A	N/A
*D. arecae* (syn. *D. endocitricola*)	ZHKUCC 20-0012^T^	*Citrus grandis*	China	MT355682	MT409336	MT409290	MT409312	N/A
	ZHKUCC 20-0013^PT^	*Citrus grandis*	China	MT355683	MT409337	MT409291	MT409313	N/A
*D. arecae* (syn. *D. fraxini-angustifoliae*)	BRIP 54781^IT^	*Fraxinus angustifolia*	Australia	JX862528	JX862534	KF170920	N/A	N/A
	MFLUCC 15-0748	*Vitis vinifera*	China	KT459428	KT459446	KT459430	KT459462	N/A
*D. arecae* (syn. *D. fulvicolor*)	CGMCC 3.19601^T^	*Pyrus pyrifolia*	China	MK626859	MK654806	MK691236	MK691132	MK726163
	PSCG 057	*Pyrus pyrifolia*	China	MK626858	MK654810	MK691233	MK691131	MK726164
*D. arecae* (syn. *D. gossiae*)	BRIP 59730a^H^	*Sesbania* sp.	Australia	OM918693	OM960602	OM960620	N/A	N/A
*D. arecae* (syn. *D. guangxiensis*)	JZB 320091	*Vitis vinifera*	China	MK335769	MK523564	MK500165	MK736724	N/A
	JZB 320094^T^	*Vitis vinifera*	China	MK335772	MK523566	MK500168	MK736727	N/A
*D. arecae* (syn. *D. hongheensis*)	KUMCC 21-0457^T^	*Mangifera indica*	China	OM001331	ON468649	ON468658	ON715010	N/A
	KUMCC 21-0458	*Mangifera indica*	China	OM001330	ON468650	ON468659	ON715009	N/A
*D. arecae* (syn. *D. howardiae*)	BRIP 59697a^H^	*Agave* sp.	Australia	OM918695	OM960604	OM960622	N/A	N/A
*D. arecae* (syn. *D. huangshanensis*)	CNUCC 201903^T^	*Camellia oleifera*	China	MN219729	MN224670	MN227010	N/A	MN224558
	CNUCC 201904^PT^	*Camellia oleifera*	China	MN219730	MN224671	MN227011	N/A	MN224559
*D. arecae* (syn. *D. hunanensis*)	HNZZ023^T^	*Camellia oleifera*	China	MZ509550	MZ504702	MZ504713	MZ504680	MZ504691
	HNZZ025	*Camellia oleifera*	China	MZ509551	MZ504703	MZ504714	MZ504681	MZ504692
*D. arecae* (syn. *D. krabiensis*)	MFLUCC 17-2481^T^	Submerged wood	Thailand	MN047101	MN433215	MN431495	N/A	N/A
*D. arecae* (syn. *D. limonicola*)	CBS 142549^T^	*Citrus limon*	Malta	NR_154980	MF418501	MF418582	MF418256	MF418342
	CBS 142550	*Citrus limon*	Malta	MF418423	MF418502	MF418583	MF418257	MF418343
	CPC 27869	*Citrus limon*	Malta	MF418419	MF418498	MF418579	MF418253	MF418339
	HNHK02	*Areca catechu*	China	MN424515	MN424557	MN424529	MN424571	MN424543
	HNQH03	*Areca catechu*	China	MN424526	MN424568	MN424540	MN424582	MN424554
	HNQH02	*Areca catechu*	China	MN424525	MN424567	MN424539	MN424581	MN424553
*D. arecae* (syn. *D. liquidambaris*)	SCHM 3621^H^	*Liquidambar formosana*	China	AY601919	N/A	N/A	N/A	N/A
*D. arecae* (syn. *D. litchiicola*)	BRIP 54900^T^	*Litchi chinensis*	Australia	JX862533	JX862539	KF170925	N/A	N/A
*D. arecae* (syn. *D. loropetali*)	SCHM 3615^H^	*Loropetalum chinense*	China	AY601917	N/A	N/A	N/A	N/A
*D. arecae* (syn. *D. meliae*)	CFCC 53089^T^	*Melia azedarach*	China	MK432657	ON081654	MK578057	N/A	ON081662
	CFCC 53090	*Melia azedarach*	China	MK432658	ON081655	MK578058	N/A	ON081663
*D. arecae* (syn. *D. melitensis*)	CBS 142551^T^	*Citrus limon*	Malta	MF418424	MF418503	MF418584	MF418258	MF418344
	CPC 27875	*Citrus limon*	Malta	MF418425	MF418504	MF418585	MF418259	MF418345
*D. arecae* (syn. *D. millettiae*)	GUCC 9167^T^	Plant foliage	China	MK398674	MK480609	MK502089	MK502086	N/A
	MFLUCC 20-0183	*Celtis formosana*	China	MW114351	MW192214	MW148271	MW151589	N/A
*D. arecae* (syn. *D. musigena*)	CBS 129519^T^	*Musa* sp.	Australia	KC343143	KC343869	KC344111	KC343385	KC343627
*D. arecae* (syn. *D. nelumbonis*)	BCRC FU30382^R^	*Nelumbo nucifera*	China	KT821501	N/A	LC069368	N/A	N/A
*D. arecae* (syn. *D. norfolkensis*)	BRIP 59718a^H^	*Mangifera indica*	Australia	OM918699	OM960608	OM960626	N/A	N/A
*D. arecae* (syn. *D. oculi*)	MAFF 246252^T^	*Homo sapiens*	Japan	LC373514	LC373516	LC373518	N/A	N/A
*D. arecae* (syn. *D. osmanthi*)	GUCC 9165^T^	*Camellia sinensis*	China	MK398675	MK480610	MK502091	MK502087	N/A
	SAUCC 194.21	*Camellia sinensis*	China	MT822549	MT855862	MT855746	MT855634	MT855518
*D. arecae* (syn. *D. pandanicola*)	MFLUCC 17-0607^T^	*Pandanus* sp.	Thailand	MG646974	N/A	MG646930	N/A	N/A
	SAUCC 194.82	*Milletia reticulata*	China	MT822610	MT855922	MT855807	MT855689	MT855578
*D. arecae* (syn. *D. pascoei*)	BRIP 54847^IT^	*Persea americana*	Australia	JX862532	JX862538	KF170924	N/A	N/A
*D. arecae* (syn. *D. pescicola*)	MFLUCC 16-0105^T^	*Prunus persica*	China	KU557555	KU557623	KU557579	KU557603	N/A
	MFLUCC 16-0108	*Prunus persica*	China	KU557558	KU557626	KU557582	KU557606	N/A
	PSCG 036	*Pyrus* × *bretschneideri*	China	MK626855	MK654796	MK691226	MK691116	MK726159
	PSCG 037	*Pyrus* × *bretschneideri*	China	MK626857	MK654799	MK691230	MK691130	MK726157
*D. arecae* (syn. *D. phyllanthicola*)	SCHM 3680^H^	*Phyllanthus emblicae*	China	AY620819	N/A	N/A	N/A	N/A
*D. arecae* (syn. *D. podocarpi-macrophylli*)	CGMCC 3.18281^T^	*Podocarpus macrophyllus*	Japan	KX986774	KX999167	KX999207	KX999278	KX999246
	LC 6229	*Olea europaea*	Italy	KX986771	KX999164	KX999204	KX999277	KX999243
*D. arecae* (syn. *D. pseudomangiferae*)	CBS 101339^T^	*Mangifera indica*	Dominican Republic	KC343181	KC343907	KC344149	KC343423	KC343665
	CBS 388.89	*Mangifera indica*	Mexico	KC343182	KC343908	KC344150	KC343424	KC343666
*D. arecae* (syn. *D. pseudooculi*)	MAFF 246452^T^	*Homo sapiens*	Japan	LC373515	LC373517	LC373519	N/A	N/A
*D. arecae* (syn. *D. pseudophoenicicola*)	CBS 176.77	*Mangifera indica*	Iraq	KC343183	KC343909	KC344151	KC343425	KC343667
	CBS 462.69^T^	*Phoenix dactylifera*	Spain	KC343184	KC343910	KC344152	KC343426	KC343668
	CDP 0047	*Chamaerops humilis*	Portugal	MT002357	MT011069	MT011075	MT011065	N/A
	CDP 0358	*Phoenix dactylifera*	Portugal	MT004743	MT011073	MT011079	MT011067	N/A
	HNHK01	*Areca catechum*	China	MN424514	MN424556	MN424528	MN424570	MN424542
	HNHK03	*Areca catechum*	China	MN424516	MN424558	MN424530	MN424572	MN424544
	HNQZ01	*Areca catechum*	China	MN424520	MN424562	MN424534	MN424576	MN424548
	HNWC01	*Areca catechum*	China	MN424517	MN424559	MN424531	MN424573	MN424545
	HNWC02	*Areca catechum*	China	MN424518	MN424560	MN424532	MN424574	MN424546
	HNWN03	*Areca catechum*	China	MN424524	MN424566	MN424538	MN424580	MN424552
	LC 6150	*Phoenix canariensis*	Uruguay	KY011891	KY011902	N/A	N/A	N/A
	LC 6151	*Phoenix canariensis*	Uruguay	KY011892	KY011903	N/A	N/A	N/A
*D. arecae* (syn. *D. pterocarpicola*)	MFLUCC 10-0580a^T^	*Pterocarpus indicus*	Thailand	JQ619887	JX275403	JX275441	JX197433	N/A
	MFLUCC 10-0580b^IT^	*Pterocarpus indicus*	Thailand	JQ619888	JX275404	JX275442	JX197434	N/A
*D. arecae* (syn. *D. schimae*)	CFCC 53103^T^	*Schima superba*	China	MK432640	MK578116	MK578043	MK442962	MK442987
	CFCC 53104	*Schima superba*	China	MK432641	MK578117	MK578044	MK442963	MK442988
*D. arecae* (syn. *D. searlei*)	BRIP 66528^T^	*Macadamia* sp.	South Africa	MN708231	N/A	MN696540	N/A	N/A
*D. arecae* (syn. *D. sennae*)	CFCC 51636^T^	*Cassia bicapsularis*	China	KY203724	KY228885	KY228891	KY228875	N/A
	CFCC 51637^PT^	*Cassia bicapsularis*	China	KY203725	KY228886	KY228892	KY228876	N/A
*D. arecae* (syn. *D. spinosa*)	CGMCC 3.19602^T^	*Pyrus pyrifolia*	China	MK626849	MK654811	MK691234	MK691129	MK726156
	PSCG 388	*Pyrus pyrifolia*	China	MK626860	MK654798	MK691229	MK691128	MK726171
*D. arecae* (syn. *D. taiwanensis*)	NTUCC 18-105-1^T^	*Ixora* sp.	China	MT241257	MT251199	MT251202	MT251196	N/A
	NTUCC 18-105-2	*Ixora* sp.	China	MT241258	MT251200	MT251203	MT251197	N/A
*D. arecae* (syn. *D. taoicola*)	MFLUCC 16-0117^T^	*Prunus persica*	China	KU557567	KU557635	KU557591	N/A	N/A
	MFLUCC 16-0118	*Prunus persica*	China	KU557568	KU557636	KU557592	N/A	N/A
	PSCG 485	*Pyrus pyrifolia*	China	MK626869	MK654812	MK691227	MK691120	MK726173
*D. arecae* (syn. *D. viciae*)	JZB 320179^T^	*Vicia villosa*	China	OP626092	OP627280	OP627281	N/A	OP627279
*D. arecae* (syn. *D. viniferae*)	JZB 320071^T^	*Vitis vinifera*	China	MK341550	MK500107	MK500112	MK500119	N/A
	JZB 320072	*Vitis vinifera*	China	MK341551	MK500108	MK500113	MK500120	N/A
*D. arecae* (syn. *D. annellsiae*)	BRIP 59731a^H^	*Mangifera indica*	Australia	OM918687	OM960596	OM960614	N/A	N/A
*D. chiangmaiensis*	MFLUCC 18-0544^T^	*Magnolia liliifera*	Thailand	OK393703	OL439483	N/A	N/A	N/A
	MFLUCC 18-0935	*Magnolia liliifera*	Thailand	OK393704	OL439484	N/A	N/A	N/A
*D. chiangmaiensis* (“*D.* cf. *heveae* 2”)	CBS 681.84	*Hevea brasiliensis*	India	KC343117	KC343843	KC344085	KC343359	KC343601
*D. chiangmaiensis* (“*D.* cf. *heveae*”)	BR74	*Calamus castaneus*	Malaysia	MN651490	MT077091	MT077079	N/A	N/A
	SM36	*Calamus castaneus*	Malaysia	MN651489	MT077092	MT077080	N/A	N/A
*D. citri*	CBS 134239	*Citrus sinensis*	USA	KC357553	KC357522	KC357456	KC357488	MF418280
	CBS 135422^ET^	*Citrus* sp.	USA	KC843311	KC843071	KC843187	KC843157	MF418281
*D. corylicola*	CFCC 53986^T^	*Corylus heterophylla*	China	MW839880	MW815894	MW883977	MW836684	MW836717
	CFCC 54696	*Corylus heterophylla*	China	MW839881	MW815907	MW883990	MW836697	MW836730
*D. longicolla*	ATCC 60325^T^	*Glycine max*	USA	KJ590728	KJ590767	KJ610883	KJ612124	KJ659188
	CBS 116023	*Glycine max*	USA	KC343198	KC343924	KC344166	KC343440	KC343682
*D. sennicola*	CFCC 51634^T^	*Cassia bicapsularis*	China	KY203722	KY228883	KY228889	KY228873	KY228879
	CFCC 51635	*Cassia bicapsularis*	China	KY203723	KY228884	KY228890	KY228874	KY228880
*D. smilacicola*	CFCC 54582^T^	*Smilax glabra*	China	OP955933	OP959770	OP959776	OP959779	OP959788
	CFCC 58764	*Smilax glabra*	China	OP955934	OP959769	OP959775	OP959778	OP959787

^1^ Taxon or strain’s previous name is noted in brackets if different from current name for taxa which were synonymized (indicated by syn.) or resolved in the present study; ^2^ Acronyms of culture collections, ATCC: American Type Culture Collection, Virginia, USA; BCRC: Bioresource Collection and Research Center, Taiwan; BRIP: Plant Pathology Herbarium, Department of Primary Industries, Dutton Park, Queensland, Australia; CBS: CBS-KNAW Fungal Biodiversity Centre, Utrecht, The Netherlands; CDP: culture collection of D.S. Pereira, housed at the Lab Bugworkers|M&B-BioISI|Tec Labs—Innovation Centre, Faculty of Sciences, University of Lisbon, Lisbon, Portugal; CFCC: China Forestry Culture Collection Center, Beijing, China; CGMCC: China General Microbiological Culture Collection Center, China; CNUCC: Capital Normal University Culture Collection Center, Beijing, China; CPC: working collection of P.W. Crous, housed at CBS; GUCC: Guizhou University Culture Collection; JZB: culture collection of Institute of Plant and Environmental Protection, Beijing Academy of Agriculture and Forestry Sciences, Beijing 100097, China; KUMCC: Culture Collection of Kunming Institute of Botany, Kunming, China; LC: working collection of Lei Cai, housed at Laboratory State Key Laboratory of Mycology, Institute of Microbiology, Chinese Academy of Sciences, China; MAFF: Gene Bank Project, Ministry of Agriculture, Forestry and Fisheries, Tsukuba, Japan; MFLU: Herbarium of Mae Fah Luang University, Chiang Rai, Thailand; MFLUCC: Mae Fah Luang University Culture Collection, Chiang Rai, Thailand; NTUCC: Department of Plant Pathology and Microbiology, National Taiwan University Culture Collection, Taiwan, China; PSCG: personal culture collection of Y.S. Guo, China; SAUCC: Shandong Agricultural University Culture Collection, China; SCHM: Mycological Herbarium of South China Agricultural University, Guangzhou, China; ZHKUCC: University of Agriculture and Engineering Culture Collection, China. ^3^ Status of the strains or specimens are noted by bold superscript ET (ex-epitype), H (holotype), IT (ex-isotype), PT (ex-paratype), R (reference) and T (ex-type); ^4^ N/A: sequences not available; *cal*: partial calmodulin gene; ITS: partial cluster of nrRNA genes, including the nuclear 5.8S rRNA gene and its flanking internal transcribed spacer regions ITS1 and ITS2; *tef1*: partial translation elongation factor 1-alpha gene; *tub2*: partial beta-tubulin gene.

**Table 2:** Synopsis of the alignment properties, statistics, results and nucleotide substitution models used for phylogenetic analyses.

Analysis ^1^	Characters Summary	5-loci Dataset ^2^					
		ITS	*tef1*	*tub2*	*cal*	*his3*	Combined
Number of strains/number of species		60/29, including 8/4 as outgroup taxa					
Total characters		490	341	376	461	456	2124
Invariable characters (%)		394 (80.4%)	168 (49.3%)	247 (65.7%)	292 (63.3%)	323 (70.8%)	1424 (67.0%)
MP	Parsimony-informative characters (%)	85 (17.3%)	165 (48.4%)	115 (30.6%)	155 (33.6%)	116 (25.4%)	636 (29.7%)
	Parsimony-uninformative characters	11	8	14	14	17	64
	Tree length (TL)	206	322	231	364	236	1655
	Consistency index (CI)	0.558	0.730	0.693	0.629	0.763	0.555
	Homoplasy index (HI)	0.442	0.270	0.307	0.371	0.237	0.445
	Retention index (RI)	0.875	0.885	0.872	0.821	0.893	0.778
	Rescaled consistency index (RC)	0.488	0.646	0.604	0.516	0.681	0.431
ML/BA	Unique alignment patterns/alignment sites (%)	112/484 (23.1%)	173/331 (52.3%)	132/373 (35.4%)	183/461 (39.7%)	151/456 (33.1%)	751/2105 (35.7%)
	Invariant sites (%)	80.2%	47.7%	65.4%	63.3%	70.8%	66.5%
	Undetermined characters or gaps (%)	7.8%	8.6%	7.9%	8.4%	7.4%	8.0%
	Nucleotide substitution models *	TN93 +G+I	HKY +G	TN93 +G	GTR +G+I	GTR +G	Partitioned
**Analysis ^1^**	**Characters summary**	**4-loci dataset ^2^**					
		**ITS**	***tef1***	***tub2***	***cal***	**Combined**	
Number of strains/number of species		83/39, including 8/4 as outgroup taxa					
Total characters		490	341	376	461	1668	
Invariable characters (%)		383 (78.2%)	167 (49.0%)	241 (64.1%)	269 (58.4%)	1060 (63.5%)	
MP	Parsimony-informative characters (%)	94 (19.2%)	169 (49.6%)	119 (31.6%)	159 (34.5%)	541 (32.4%)	
	Parsimony-uninformative characters	13	5	16	33	67	
	Tree length (TL)	250	344	261	431	1642	
	Consistency index (CI)	0.532	0.692	0.644	0.608	0.488	
	Homoplasy index (HI)	0.468	0.308	0.356	0.392	0.512	
	Retention index (RI)	0.887	0.888	0.867	0.819	0.767	
	Rescaled consistency index (RC)	0.472	0.614	0.558	0.498	0.374	
ML/BA	Unique alignment patterns/alignment sites (%)	130/488 (26.6%)	182/331 (55.0%)	145/374 (38.8%)	201/461 (43.6%)	658/1654 (39.78%)	
	Invariant sites (%)	78.1%	47.4%	63.9%	58.4%	63.3%	
	Undetermined characters or gaps (%)	8.3%	8.6%	8.4%	8.3%	8.4%	
	Nucleotide substitution models *	TN93 +G+I	GTR +G	GTR +G+I	GTR +G+I	Partitioned	
**Analysis** **^1^**	**Characters summary**	**3-loci dataset** **^2^**					
		**ITS**	***tef1***	***tub2***	**Combined**		
Number of strains/number of species		114/53, including 8/4 as outgroup taxa					
Total characters		490	341	376	1207		
Invariable characters (%)		374 (76.3%)	156 (45.7%)	224 (59.6%)	754 (62.5%)		
MP	Parsimony-informative characters (%)	98 (20.0%)	174 (51.0%)	130 (34.6%)	402 (33.3%)		
	Parsimony-uninformative characters	18	11	22	51		
	Tree length (TL)	303	476	358	1518		
	Consistency index (CI)	0.488	0.592	0.567	0.417		
	Homoplasy index (HI)	0.512	0.408	0.433	0.583		
	Retention index (RI)	0.891	0.851	0.835	0.758		
	Rescaled consistency index (RC)	0.435	0.504	0.473	0.316		
ML/BA	Unique alignment patterns/alignment sites (%)	143/490 (29.2%)	206/341 (60.4%)	167/376 (44.4%)	516/1207 (42.8%)		
	Invariant sites (%)	8.3%	11.1%	8.9%	9.3%		
	Undetermined characters or gaps (%)	76.3%	45.8%	59.6%	62.5%		
	Nucleotide substitution models *	TN93 +G+I	GTR +G+I	GTR +G+I	Partitioned		

^1^ BA: Bayesian analysis; ML: maximum likelihood; MP: maximum parsimony; ^2^ *cal*: partial calmodulin gene; *his3*: partial histone H3 gene; ITS: partial cluster of nrRNA genes, including the nuclear 5.8S rRNA gene and its flanking internal transcribed spacer regions ITS1 and ITS2; *tef1*: partial translation elongation factor 1-alpha gene; *tub2*: partial beta-tubulin gene; * G, I: models of evolution assuming a discrete gamma distribution (G) and/or estimation of proportion of invariable sites (I); GTR: general time reversible model; HKY: Hasegawae–Kishonoe–Yano model; TN93: Tamura–Nei model.

**Table 3:** Pairwise homoplasy index (PHI) test results for recombination of the *Diaporthe arecae* species complex based on concatenated and single gene sequence alignments of the 5-, 4- and 3-loci combined datasets.

Dataset Tested ^1^	Φ*_w_*-Statistic (*p*-Value) ^2^		
	5-loci	4-loci	3-loci
ITS	0.19 (4.34 × 10^−4^) *	0.23 (0.01) *	0.27 (0.02) *
*tef1*	0.11 (0.02) *	0.13 (2.94 × 10^−3^) *	0.17 (1.12 × 10^−3^) *
*tub2*	0.10 (0.07)	0.12 (9.92 × 10^−3^) *	0.14 (0.23)
*cal*	0.20 (0.10)	0.19 (0.99)	N/A
*his3*	0.13 (0.61)	N/A	N/A
Combined	0.16 (0.00) *	0.19 (0.00) *	0.22 (0.00) *

^1^ *cal*: partial calmodulin gene; *his3*: partial histone H3 gene; ITS: partial cluster of nrRNA genes, including the nuclear 5.8S rRNA gene and its flanking internally transcribed spacer regions ITS1 and ITS2; *tef1*: partial translation elongation factor 1-alpha gene; *tub2*: partial beta-tubulin gene; ^2^ 5-loci: combined dataset based on ITS, *tef1*, *tub2*, *cal* and *his3* loci; 4-loci: combined dataset based on ITS, *tef1*, *tub2* and *cal* loci; 3-loci: combined dataset based on ITS, *tef1* and *tub2* loci; N/A: not applicable, locus excluded from the dataset; * Positive for recombination, PHI test yielded a *p* < 0.05.

**Table 4:** Genetic diversity and neutrality analysis of the *Diaporthe arecae* species complex.

Dataset Tested ^1^		N ^2^	Measures of Genetic Diversity ^2^						Tajima’s D ^3^
			h	S	Hd ± SD	π ± SD	η	θ	
5-loci	ITS	52	31	51	0.977 ± 0.008	0.030 ± 0.002	57	0.031	−0.09823 ^ns^
	*tef1*	52	31	72	0.961 ± 0.016	0.037 ± 0.003	75	0.060	−1.31045 ^ns^
	*tub2*	52	27	67	0.963 ± 0.012	0.033 ± 0.001	71	0.047	−1.06861 ^ns^
	*cal*	52	33	73	0.965 ± 0.015	0.032 ± 0.002	91	0.057	−1.52753 ^ns^
	*his3*	52	27	58	0.963 ± 0.011	0.021 ± 0.003	62	0.035	−1.43297 ^ns^
	Combined	52	42	321	0.992 ± 0.005	0.030 ± 0.002	356	0.045	−1.18300 ^ns^
4-loci	ITS	75	40	52	0.980 ± 0.005	0.028 ± 0.001	58	0.029	−0.09624 ^ns^
	*tef1*	75	38	63	0.969 ± 0.009	0.034 ± 0.002	67	0.054	−1.19098 ^ns^
	*tub2*	75	36	67	0.967 ± 0.009	0.028 ± 0.001	71	0.047	−1.32256 ^ns^
	*cal*	75	43	99	0.975 ± 0.008	0.031 ± 0.002	122	0.071	−1.92574 ^ss^
	Combined	75	57	281	0.993 ± 0.003	0.030 ± 0.001	318	0.049	−1.33832 ^ns^
3-loci	ITS	106	60	59	0.987 ± 0.003	0.028 ± 0.001	65	0.031	−0.25953 ^ns^
	*tef1*	106	52	85	0.974 ± 0.006	0.035 ± 0.002	105	0.081	−1.86768 ^ss^
	*tub2*	106	56	88	0.980 ± 0.005	0.029 ± 0.001	107	0.066	−1.83873 ^ss^
	Combined	106	81	233	0.995 ± 0.002	0.030 ± 0.001	277	0.055	−1.51365 ^ns^

^1^ *cal*: partial calmodulin gene; *his3*: partial histone H3 gene; ITS: partial cluster of nrRNA genes, including the nuclear 5.8S rRNA gene and its flanking internally transcribed spacer regions ITS1 and ITS2; *tef1*: partial translation elongation factor 1-alpha gene; *tub2*: partial beta-tubulin gene; ^2^ h: number of haplotypes; Hd: haplotype (gene) diversity; N: sample size, i.e., number of sequences (taxa); S: number of polymorphic (segregating) sites; SD: standard deviation; η: total number of mutations, Eta; π: nucleotide diversity (per site), Pi; θ: Watterson estimator (theta (per site) from Eta); ^3^ Statistical significance is noted as superscript ns (not statistically significant, *p* > 0.10) and superscript ss (statistically significant, *p* < 0.05).

**Table 5:** Synopsis of the morphological data on asexual morphs of *Diaporthe arecae*, including the synonyms proposed in this study.

Taxon ^1^	Conidiomata	Conidiogenous Layer ^2^	Conidia	Reference
*Diaporthe arecae* (H.C. Srivast., Zakia & Govindar.) R.R. Gomes, C. Glienke & Crous ≡ *Subramanella arecae* H.C. Srivast., Zakia & Govindar. (CBS H-7808^IH^)	*Pycnosclerotium* formed along the sclerotium cortex, lacking ostiole, exuding conidia through irregular openings, 160–360 × 240–860 μm	*Conidiophores* distinct, long, thin, hyaline, simple	*Alpha conidia* elliptic, hyaline, aseptate, 7.2–9.6 × 2.4 μm *Beta-conidia* needle-shaped, slightly curved, hyaline, aseptate, 14.4–24 × 1.2 μm *Gamma conidia* not observed	[24,38]
*Diaporthe acuta* Y.S. Guo & G.P. Wang * (CGMCC 3.19600^T^)	*Pycnidia* globose or irregular, dark brown to black, 230–544 μm diam.	N/A	*Alpha conidia* fusiform to oval, acutely rounded ends, hyaline, aseptate, bi- or multiguttulate, 6–9.5 × 2–3 μm ($\overset{-}{x}$ = 7.8 × 2.6 μm, n = 50; L/W = 3) *Beta* and *gamma conidia* not observed	[104]
*Diaporthe anhuiensis* H. Zhou & C.L. Hou * (CNUCC 201901^T^)	*Pycnidia* globose, fuscous to black, exuding whitish to cream conidial droplets from ostiole, 250–340 μm diam.	*Conidiophores* cylindrical, tapering towards apex, hyaline, unbranched, 10.5–25.2 × 1.5–2.7 μm	*Alpha conidia* spindly or fusoid, hyaline, aseptate, bi-guttulate, rarely multiguttulate, 7.6–10.4 × 2.2–3.6 μm ($\overset{-}{x}$ = 8.8 × 2.8 μm, n = 40) *Beta* and *gamma conidia* not observed	[105]
*Diaporthe arengae* R.R. Gomes, C. Glienke & Crous * (CBS 114979^T^)	*Pycnidia* subglobose, black, exuding cream conidial droplets through central ostiole, up to 250 μm diam.	*Conidiophores* cylindrical, straight to sinuous, hyaline apex, pale brown base, 0–6-septate smooth, branched, densely aggregated, 10–60 × 2.5–4 μm *Conidiogenous cells* cylindrical, terminal and lateral, slightly tapering towards apex (1–1.5 μm), phialidic (with periclinal thickening), with collarette not flared (up to 2 μm long), 8–15 × 1.5–2.5 μm	*Alpha conidia* fusoid-ellipsoid, tapering towards ends, subobtuse apex, flattened hilum at base, hyaline, aseptate, guttulate, (5–)6–7(–9) × (2–)2.5(–3) μm *Beta conidia* rarely observed, subcylindrical, bluntly rounded apex, truncate base, hyaline, aseptate, smooth, 20–25 × 1.5 μm *Gamma conidia* not observed	[24]
*Diaporthe averrhoae* (C.Q. Chang, Z.D. Jiang & P.K. Chi) Y.H. Gao & L. Cai * ≡ *Phomopsis averrhoae* C.Q. Chang, Z.D. Jiang & P.K. Chi (SCHM 3605^H^)	*Pycnidia* of eustroma, compressed triangle or triangle, unilocular, brown to dark brown, with thinner wall at the base, 188–388 × 83–175 μm	*Conidiophores* hyaline, septate, branched, 8.5–36 × 1.4–2.0 μm *Conidiogenous cells* hyaline, phialidic	*Alpha conidia* fusiform, hyaline, aseptate, biguttulate, 6.0–8.4 × 1.4–1.8 μm *Beta conidia* filiform, mostly hamate, hyaline, aseptate, 10–25.5 × 0.5–0.9 μm G*amma conidia* not observed	[26,106]
*Diaporthe* *camelliae-oleiferae* Q. Yang * (HNZZ027^T^)	*Pycnidia* globose, dark brown to black, exuding pale-yellow conidial droplets from ostiole, 500–660 μm diam.	*Conidiophores* reduced to conidiogeneous cells. *Conidiogenous cells* cylindrical, tapering towards apex, straight, terminal, aseptate, densely aggregated, (7.5–)10–14(–15.5) × 1.5–2.3 μm (n = 30)	*Alpha conidia* ellipsoidal to fusiform, hyaline, aseptate, bi-guttulate, 5–6.5(–7.5) × 1.9–2.3 μm (n = 30) *Beta conidia* filiform, sinuous at one end, hyaline, aseptate, eguttulate, (26.5–)28.5–31(–33) × 0.8–1.2 μm (n = 30) G*amma conidia* not observed	[107]
*Diaporthe ceratozamiae* Crous & R.G. Shivas * (CBS 131306^T^)	*Pycnidia* subglobose, black, exuding yellow conidial droplets from ostiole, up to 300 μm diam.	*Conidiophores* cylindrical, straight to sinuous, hyaline, 1–3-septate, smooth, branched, densely aggregated, 15–30 × 3–4 μm *Conidiogenous cells* cylindrical, terminal, and lateral, slightly tapering towards apex (1–1.5 μm), phialidic (with periclinal thickening), with collarette not flared (1 μm long) *Paraphyses* cylindrical, straight, flexuous, hyaline, usually 1–2-septate at base, smooth, wall thickened, unbranched or branched at base, extending above conidiophores, up to 60 μm long and 1.5–2.5 μm wide at base	*Alpha conidia* fusiform, tapering towards ends, acutely rounded apex, subtruncate base, hyaline, aseptate, (6.5–)8–9(–10) × 2–2.5(–3) μm *Beta conidia* and *gamma conidia* not observed	[108]
*Diaporthe cercidis* C.M. Tian & Q. Yang * (CFCC 52565^T^)	*Pycnidia* discoid (ectostromatic disc), with a solitary undivided circular locule, nearly flat, grey to brown, with one ostiole, 135–200 μm diam.	*Conidiophores* cylindrical, tapering towards apex, straight or slightly curved, unbranched, phialidic, 7–17 × 1.4–2.1 μm	*Alpha conidia* fusiform to oval, hyaline, aseptate, bi-guttulate, 6.5–10 × 3–3.5 μm ($\overset{-}{x}$ = 8.6 × 3.3 μm, n = 30) *Beta conidia* filiform, straight or hamate, hyaline, aseptate, eguttulate, 20–28.5 × 1–1.3 μm ($\overset{-}{x}$ = 25.5 × 1.2 μm, n = 30) G*amma conidia* not observed	[109]
*Diaporthe chamaeropicola* D.S. Pereira & A.J.L. Phillips * (CDP 0460^T^)	*Pycnidia* subglobose, dark-brown to black, lacking an ostiole, exuding a creamy mucoid conidial mass through irregular fissures on pycnidial wall, up to 4 mm diam.	*Conidiophores* reduced to conidiogenous cells *Conidiogenous cells* cylindrical, occasionally ampulliform, tapering towards apex, straight, hyaline, aseptate or 1–3-septate, smooth, unbranched or branched, with collarette (up to 1 µm long), enteroblastic (with periclinal thickening and 1–2 annellations), dimorphic, short conidiogenous cells 4.9–19.4 × 0.9–2.6 µm ($\overset{-}{x}$ = 13.66 × 1.75 µm), long conidiogenous cells 15.2–49.2 × 1.1–2.7 µm ($\overset{-}{x}$ = 29.54 × 1.75 µm) *Paraphyses* cylindrical, straight, flexuous, tapering towards apex, hyaline, 1–2(–3)-septate at base, smooth, unbranched or branched at base, extending above conidiogeneous cells, 26.6–78.8 μm ($\overset{-}{x}$ = 53.57 µm) long	*Alpha conidia* cylindrical to ellipsoidal, rounded apex, obtuse to truncate base, straight to slightly curved, hyaline, aseptate, smooth, biguttulate, 5.6–9.4 × 1.7–3 μm ($\overset{-}{x}$ = 7.53 × 2.31 µm, L/W = 3.33) *Beta* and *gamma conidia* not observed	[64]
*Diaporthe chrysalidocarpi* S.T. Huang, J.W. Xia, W.X. Sun, & X.G. Zhang * (SAUCC 194.35^T^)	*Pycnidia* subglobose, black, exuding white or yellowish creamy conidial droplets from central ostiole	*Conidiophores* subcylindrical, swelling at base, straight or curved, hyaline, septate, smooth, branched, 27.5–35 × 1.4–2 μm *Conidiogenous cells* cylindrical, tapering towards apex, terminal, straight or sinuous, phialidic, 10.5–23 × 1.4–1.8 μm	*Beta conidia* filiform, subtruncate base, tapering towards base, straight or slightly curved, hyaline, aseptate, 28–32.5 × 1.2–1.6 μm ($\overset{-}{x}$ = 30.3 × 1.3 μm, n = 20) *Alpha* and g*amma conidia* not observed	[110]
*Diaporthe delonicis* R.H. Perera, E.B.G. Jones & K.D. Hyde * (MFLU 16-1059^H^)	*Pycnidia* globose or near-globose, brown to dark brown, exuding white creamy conidial droplets, 78–190 μm ($\overset{-}{x}$ = 120 μm) diam.	*Conidiophores* subcylindrical, hyaline, 6.4–15.2 × 1.4–2.2 μm ($\overset{-}{x}$ = 11.6 × 1.9 μm) *Conidiogenous cells* cylindrical, tapering towards apex, with prominent collarette, phialidic, 5.3–10.5 × 1.3–2.5 μm ($\overset{-}{x}$ = 7.9 × 1.9 μm)	*Alpha conidia* fusoid, obtuse ends, hyaline, aseptate, 4-guttulate, smooth, 4.4–9 × 1.3–2.2 μm ($\overset{-}{x}$ = 7.7 × 1.8 μm) *Beta conidia* filiform, slightly curved at one end, rounded ends, hyaline, aseptate, smooth, 16–23 × 1–1.7 μm ($\overset{-}{x}$ = 19.4 × 1.2 μm) G*amma conidia* not observed	[111]
*Diaporthe drenthii* Y.P. Tan, Akinsanmi & R.G. Shivas * (BRIP 66524^T^)	*Pycnidia* globose or irregular, dark brown to black, up to 1 mm diam.	*Conidiophores* hyaline, smooth, densely aggregated, 15–25 μm long *Conidiogeneous cells* cylindrical, straight or flexuous, hyaline, phialidic, 10–20 × 1–2.5 μm	*Alpha conidia* fusiform, acute ends, hyaline, aseptate, 5.5–8.5 × 1.5–2 μm *Beta conidia* sparse, curved, 25–35 × 1 μm *Gamma conidia* not observed	[112]
*Diaporthe endocitricola* Z.Y. Dong, M. Luo, M.M. Xiang & K.D. Hyde * (ZHKUCC 20-0012^T^)	*Pycnidia* subglobose or lageniform, multilocular, exuding hyaline to dark black creamy conidial droplets from ostiole, 124–790 × 111–635 μm ($\overset{-}{x}$ = 353 × 289 μm)	*Conidiophores* cylindrical, hyaline, 12–40 × 1–3 μm ($\overset{-}{x}$ = 26 × 2 μm)	*Alpha conidia* cylindrical to ellipsoid, hyaline, aseptate, multi-guttulate, 6–8 × 2–3 μm ($\overset{-}{x}$ = 7 × 3 μm) *Beta conidia* filiform, straight or slightly curved at one end, hyaline, aseptate, 12–30 × 1–2 μm ($\overset{-}{x}$ = 19 × 2 μm) G*amma conidia* fusiform, hyaline, multi-guttulate	[113]
*Diaporthe fraxini-angustifoliae* R.G. Shivas, J. Edwards & Y.P. Tan * (BRIP 54781^IT^)	*Pycnidia* subglobose, rarely with ostiolar beaks (up to 100 μm high), exuding tan to white conidial droplets from ostiole	*Conidiophores* reduced to conidiogenous cells or cylindrical to lageniform, straight to sinuous, hyaline to pale brown, 1-septate, 5–30 × 1.5–4 μm *Conidiogenous cells* cylindrical, hyaline, tapering towards apex, phialidic, 5–15 × 1–2 μm	*Alpha conidia* scarce, cylindrical to oval, attenuated ends, hyaline to subhyaline, (4–)5–8.5(–10) × 2–3 μm *Beta conidia* abundant, flexuous to lunate, mostly curved through 45°–180° in upper third, truncate base, narrowed towards acute apex, hyaline, aseptate, (16–)17–21(–22) × 1 μm G*amma conidia* not observed	[39]
*Diaporthe fulvicolor* Y.S. Guo & G.P. Wang * (CGMCC 3.19601^T^)	*Pycnidia* globose or irregular, dark brown to black, 174–316 μm diam.	*Conidiophores* cylindrical, straight, hyaline, 1-septate, unbranched, smooth, densely aggregated, 5.5–8 × 2.5–3.5 μm *Conidiogeneous cells* ampulliform, terminal, tapering towards apex, hyaline, 6.5–10 × 1.5–2.5 μm	*Alpha conidia* fusiform to oval, acutely rounded ends, hyaline, aseptate, bi- or multi-guttulate, 7–9 × 2–3 μm ($\overset{-}{x}$ = 7.8 × 2.5 μm, n = 50; L/W = 3.1) *Beta* and *gamma conidia* not observed	[104]
*Diaporthe guangxiensis* Dissanayake, X.H. Li & K.D. Hyde * (JZB 320094^T^)	*Pycnidia* globose, dark brown to black, 250–1550 μm ($\overset{-}{x}$ = 1.1 mm, n = 20) diam.	*Conidiophores* cylindrical, straight or sinuous, slightly tapering towards apex, terminal, aseptate, densely aggregated, 21–35 × 1.5–2.5 μm ($\overset{-}{x}$ = 27 × 2 μm)	*Alpha conidia* fusiform or oval, obtuse ends, hyaline, 5.3–7.8 × 1.5–3.2 μm ($\overset{-}{x}$ = 6.8 × 2.5 μm, n = 40) *Beta conidia* filiform, hamate, tapering towards ends, hyaline, aseptate, guttulate, 20–32 × 1–1.5 μm ($\overset{-}{x}$ = 27 × 1.5 μm, n = 20) *Gamma conidia* not observed	[21]
*Diaporthe huangshanensis* H. Zhou & C. L. Hou * (CNUCC 201903^T^)	*Pycnidia* globose, brown to black, exuding whitish translucent conidial droplets from apex, 210–270 μm diam.	*Conidiophores* cylindrical, straight to sinuous, hyaline, branched, 12.1–23.5 × 1.1–2.9 μm	*Alpha conidia* ellipsoidal to olivary body, hyaline, aseptate, bi-to multi-guttulate, 5.7–8.4 × 2.7–4.5 μm ($\overset{-}{x}$ = 6.9 × 3.5 μm, n = 40) *Beta conidia* filiform, straight or hamate, partially guttulate, one end rounded and other acute and curved, 19.5–30 × 1.1–2.1 μm ($\overset{-}{x}$ = 24.1 × 1.5 μm, n = 30) *Gamma conidia* not observed	[105]
*Diaporthe hunanensis* Q. Yang * (HNZZ023^T^)	*Pycnidia* globose, black, 180–300 μm diam.	*Conidiophores* reduced to conidiogeneous cells. *Conidiogenous cells* cylindrical, straight or slightly curved, aseptate, phialidic, (8–)9–15(–16.5) × 1.7–2.1 μm (n = 30)	*Alpha conidia* ellipsoidal, obtuse ends, hyaline, aseptate, bi-guttulate, 6.5–7.5(–8.5) × 2.4–2.9 μm (n = 30) *Beta* and *gamma conidia* not observed	[107]
*Diaporthe krabiensis* Dayarathne * (MFLUCC 17-2481^T^)	*Pycnidia* globose or irregular, uniloculate or multiloculate, black, 117–145 × 130–140 μm	*Conidiophores* cylindrical, straight to sinuous, 2–3-septate, branched, densely aggregated, rarely reduced to conidiogenous cells *Conidiogenous cells* subcylindrical, tapering towards apex, hyaline, phialidic (with periclinal thickening), with flared collarette, 15–32 × 0.9–1.4 μm ($\overset{-}{x}$ = 28.5 × 1.2 μm, n = 20)	*Beta conidia* fusiform to hooked, hyaline, aseptate, smooth, 15–32 × 0.9–1.4 μm ($\overset{-}{x}$ = 28.5 × 1.2 μm, n = 20) *Alpha* and *gamma conidia* not observed	[114]
*Diaporthe limonicola* Guarnaccia & Crous * (CBS 142549^T^)	*Pycnidia* dark brown to black, exuding whitish translucent to cream conidial droplets from ostiole, 250–670 μm diam.	*Conidiophores* cylindrical, straight, hyaline, 1-septate, smooth, densely aggregated, 5–20 × 1.5–4 μm *Conidiogenous cells* cylindrical, terminal, tapering towards apex, hyaline, phialidic, 5–12 × 1–2 μm *Paraphyses* hyaline, 1–3-septate, smooth, intermingled among conidiophores, up to 90 μm long and 1–2 μm diam. at apex	*Alpha conidia* fusiform, acute ends, hyaline, aseptate, mono- to biguttulate 5.5–8.5 × 1.5–2.5 μm ($\overset{-}{x}$ = 6.8 × 2.1 μm, L/W = 2.8) *Beta conidia* filiform, curved, tapering towards ends, hyaline, aseptate, eguttulate, 15–26.5 × 1–2 μm ($\overset{-}{x}$ = 22.7 × 1.4 μm, L/W = 16.2) *Gamma conidia* fusiform to subcylindrical, acute or rounded apex, hyaline, multiguttulate, 9–15.5 × 1–2 μm ($\overset{-}{x}$ = 10.7 × 1.4 μm, L/W = 7.6)	[10]
*Diaporthe liquidambaris* (C.Q. Chang, Z.D. Jiang & P.K. Chi) Udayanga & Castl. * ≡ Phomopsis liquidambaris C.Q. Chang, Z.D. Jiang & P.K. Chi (SCHM 3621^H^)	*Pycnidia* of eustroma, tuberous or irregular, unilocular to multilocular, 143–350 × 88–250 μm	*Conidiophores* hyaline, septate, sympodially branched, 10–25 × 1.7–3.0 μm *Conidiogenous cells* hyaline, phialidic	*Alpha conidia* fusiform, acute ends, hyaline, aseptate, biguttulate, 6.5–8.1 × 1.7–2.2 μm *Beta conidia* filiform, hamate, hyaline, aseptate, 10.5–24.5 × 0.6–1 μm G*amma conidia* not observed	[106,115]
*Diaporthe litchiicola* R.G. Shivas, Grice & Y.P. Tan [as “*litchicola*”] * (BRIP 54900^T^)	*Pycnidia* subglobose, with black cylindrical ostiolate neck (up to 1.5 mm), up to 400 μm diam.	*Conidiophores* reduced to conidiogeneous cells *Conidiogenous cells* cylindrical, straight to sinuous, hyaline, tapering towards apex, smooth, 20–45 × 1.5–2 μm	*Alpha conidia* fusiform to oval, tapered at ends, cylindrical to ellipsoidal, hyaline, smooth, guttulate, (5–)6.5–9.5(–10) × 1.5–2(–2.5) μm *Beta conidia* flexuous to lunate, (17–)20–32(–37) × 1–1.5 μm G*amma conidia* not observed	[39]
*Diaporthe loropetali* (C.Q. Chang, Z.D. Jiang & P.K. Chi) Y.H. Gao & L. Cai * ≡ Phomopsis loropetali C.Q. Chang, Z.D. Jiang & P.K. Chi (SCHM 3615^H^)	*Pycnidia* of eustroma, ampullate or tuberous, unilocular, with darker and thicker wall near the ostiole, 163–338 × 88–218 μm	*Conidiophores* filiform, hyaline, septate, branched, 10–29 × 1.4–2.1 μm *Conidiogenous cells* hyaline, phialidic	*Alpha conidia* fusiform to lanceolate, acute apex, obtuse base, hyaline, aseptate, biguttulate, 6.2–8.4 × 1.5–1.9 μm *Beta conidia* filiform, straight or curved, hyaline, aseptate, 14–31 × 0.6–1.2 μm G*amma conidia* not observed	[26,116]
*Diaporthe meliae* C.M. Tian & Qin Yang * (CFCC 53089^T^)	*Pycnidia* discoid (ectostromatic disc), with an undivided locule, dark brown, with one ostiole, (325–)135–200(–385) μm diam. (n = 30)	*Conidiophores* reduced to conidiogeneous cells. *Conidiogenous cells* cylindrical, tapering towards apex, straight or slightly curved, branched, hyaline, (13.5–)15–26.5(–28) × 1.3–2.1(–2.3) μm (n = 30)	*Alpha conidia* fusiform, hyaline, aseptate, multiguttulate, (6.7–)8–9.5(–10) × (2–)2.1–2.3 μm (L/W = 3.4–4.5, n = 30) *Beta* and *gamma conidia* not observed	[117]
*Diaporthe melitensis* Guarnaccia & Crous * (CBS 142551^T^)	*Pycnidia* dark brown to black, exuding whitish translucent to yellowish conidial droplets from ostiole, 250–650 μm diam.	*Conidiophores* cylindrical, straight, hyaline, 1-septate, smooth, densely aggregated, 5–15 × 1.5–5.5 μm *Conidiogenous cells* cylindrical, terminal, tapering towards apex, hyaline, phialidic, 6–12 × 1–3 μm	*Alpha conidia* fusiform, acute ends, hyaline, aseptate, 1–4-guttulate, 4.5–7 × 1.5–3 μm ($\overset{-}{x}$ = 5.9 × 2.2 μm, L/W = 2.7) *Beta* and *gamma conidia* not observed	[10]
*Diaporthe millettiae* H. Long, K.D. Hyde & Yong Wang bis * (GUCC 9167^T^)	*Pycnidia* subglobose to irregular, with up to 1 mm necks when present, multilocular, ostiolate, 1.5–1.8 mm diam.	*Conidiophores* reduced to conidiogeneous cells or cylindrical, hyaline to pale yellowish-brown, 1-septate, 10–23 × 1–2.5 μm *Conidiogenous cells* cylindrical to flexuous, tapering towards apex, hyaline, 8–18 × 1.5–3 μm	*Alpha conidia* fusiform, narrowed towards ends, hyaline, mostly biguttulate, 4.5–9 × 2–3.5 μm *Beta conidia* scarce to abundant, flexuous to J-shaped, hyaline, 17.5–32 × 1–2 μm G*amma conidia* not observed	[118]
*Diaporthe musigena* Crous & R.G. Shivas * (CBS 129519^T^)	*Pycnidia* subglobose, with elongated black necks, exuding yellow conidial droplets through ostiole, up to 250 μm diam.	*Conidiophores* cylindrical, straight to sinuous, hyaline, 1–3-septate, smooth, branched, densely aggregated, 15–40 × 1.5–2.5 μm *Conidiogenous cells* cylindrical, terminal and lateral, slightly tapering towards apex (0.5–1 μm), phialidic (with periclinal thickening), with collarette not flared (2–5 μm long) *Paraphyses* cylindrical, straight, flexuous, hyaline, septate, unbranched or branched, extending above conidiophores, up to 80 μm long and 2–2.5 μm wide at base	*Alpha conidia* fusiform, tapering towards ends, straight to slightly curved, acutely rounded apex, subobtuse base, hyaline, aseptate, smooth, guttulate, (7–)8–10(–12) × (2–)2.5(–3) μm *Beta conidia* observed in older cultures, spindle-shaped, acutely rounded apex, truncate base, tapering more prominently in upper third, straight to curve, hyaline, aseptate, smooth, (14–)19–22(–25) × (1.5–)2 μm *Gamma conidia* ellipsoid to fusoid, acutely rounded apex, subtruncate to acutely rounded base, hyaline, aseptate, smooth, 7–9 × 4–5 μm	[119]
*Diaporthe nelumbonis* Sawada ex R. Kirschner * ≡ *Phyllosticta nelumbonis* Sawada (BPI 352726^H^) (R. Kirschner 4114^R^)	*Pycnidia* slightly applanate, brown, ostiolate, 55–87 × 80–125 μm	*Conidiophores* reduced to conidiogenous cells or with a separate basal cell that often turns into an intercalary conidiogenous cell *Conidiogenous cells* pyriform to obclavate or lageniform, conspicuously narrowed apex, terminal or intercalary, with minute periclinal thickening, (3–)4.5–7.5(−9) × 2–3 μm (n = 30) in ^H^, (6–)6.5–10(−11) × (1.5–)2–3 (n = 20) in ^R^	*Alpha conidia* oblong-ellipsoidal, straight or slightly curved, rounded apex, attenuated towards base, hyaline, aseptate, mostly biguttulate, (6–)6.5–8(−9) × 2–2.5 μm (n = 30) in ^H^, (5–)6–7 × (1.5–)2 μm (n = 30) in ^R^ *Beta* and *gamma conidia* not observed	[120]
*Diaporthe oculi* Mochiz. & Kaz. Tanaka * (MAFF 246252^T^)	*Pycnidia* globose to depressed globose, with cylindrical, central, dark brown ostiolar neck (150–480 × 80–140 μm diam.), exuding yellow to pink conidial mass, 90–250 × 110–310 μm diam.	*Conidiophores* reduced to conidiogenous cells *Conidiogeneous cells* cylindrical to lageniform, phialidic, 6–15 × 2–5 μm	*Alpha conidia* fusoid-ellipsoid, hyaline, aseptate, 5–8.5 × 2–3 μm ($\overset{-}{x}$ = 6.7–2.4 μm, L/W = 2.3–3.2, n = 50), *Beta* and *gamma conidia* not observed	[42]
*Diaporthe osmanthi* H. Long, K.D. Hyde & Yong Wang bis * (GUCC 9165^T^)	*Pycnidia* globose, subglobose or irregular, with up to 1 mm necks when present, multilocular, ostiolate, up to 1–1.5 mm diam.	*Conidiophores* reduced to conidiogeneous cells or cylindrical, hyaline to pale yellowish-brown, 1-septate, 20.5–61 × 1–3 μm *Conidiogenous cells* cylindrical to flexuous, tapering towards apex, hyaline, 10–15 × 1.5–3 μm	*Alpha conidia* fusiform, narrowed towards ends, hyaline, biguttulate, 5.5–8.5 × 2–3 μm *Beta conidia* scarce to abundant, flexuous to J-shaped, hyaline, 20–31.5 × 1–2.5 μm G*amma conidia* not observed	[118]
*Diaporthe pascoei* R.G. Shivas, J. Edwards & Y.P. Tan * (BRIP 54847^IT^)	*Pycnidia* with ostiolar beaks (mostly up to 1.5 mm high), exuding conidial droplets from ostiole	*Conidiophores* cylindrical, straight, hyaline, 1–2-septate near base, unbranched, 5–40 × 2–3 μm *Conidiogenous cells* cylindrical, terminal, hyaline, tapering towards apex, phialidic, 5–30 × 2–3 μm	*Alpha conidia* scarce, cylindrical, rounded apex, slightly attenuated base, hyaline, (3.5–)4–5 × 1–2 μm *Beta conidia* abundant, flexuous to lunate, often curved up to 90° at apex, truncated base, narrowed towards apex, hyaline, (15–)19–31(–39) × 1–1.5 μm G*amma conidia* not observed	[39]
“*Diaporthe perseae*” (CBS 151.73)	*Pycnidia* globose, black, exuding cream conidial droplets through central ostiole, up to 400 μm diam.	*Conidiophores* cylindrical, straight to sinuous, hyaline, 1–3-septate, smooth, branched, densely aggregated, 15–35 × 3–4 μm *Conidiogenous cells* cylindrical, terminal and lateral, slightly tapering towards apex (1–1.5 μm), phialidic (with periclinal thickening), with prominent collarette (up to 5 μm long), 8–17 × 1.5–2.5 μm *Paraphyses* subcylindrical, obtuse ends, hyaline, 2–4-septate, smoooth, up to 60 μm long and 3 μm diam.	*Alpha conidia* fusoid to ellipsoid, tapering towards ends, straight, subobtuse apex, subtruncate base, hyaline, aseptate, smooth, guttulate, (6–)7–8(–9) × 2(–2.5) μm *Beta conidia* spindle-shaped, tapering from lower third towards apex, curved, acutely rounded apex, truncate base, hyaline, aseptate, smooth, (15–)22–25(–28) × 1.5(–2) μm *Gamma conidia* ellipsoid to fusoid, acutely rounded apex, subtruncate base, hyaline, aseptate, smooth, 9–14× 1.5–2 μm	[24]
*Diaporthe pescicola* Dissanayake, J.Y. Yan, X.H. Li & K.D. Hyde * (MFLUCC 16-0105^T^)	*Pycnidia* globose, dark brown to black, up to 300 μm diam.	*Conidiophores* cylindrical, straight or sinuous, terminal, slightly tapering towards apex, aseptate, densely aggregated, 21–35 × 1.5–2.5 μm ($\overset{-}{x}$ = 27 × 2 μm)	*Alpha conidia* fusiform or oval, obtuse ends, hyaline, biguttulate, 6–8.5 × 2–3 μm ($\overset{-}{x}$ = 8 × 3 μm) *Beta conidia* filiform, hamate, tapering towards ends, hyaline, aseptate, 18–37 × 1–1.5 μm ($\overset{-}{x}$ = 27 × 1.5 μm) *Gamma conidia* not observed	[41]
*Diaporthe phyllanthicola* (C.Q. Chang, Z.D. Jiang & P.K. Chi) Y.H. Gao & L. Cai * ≡ *Phomopsis phyllanthicola* C.Q. Chang, Z.D. Jiang & P.K. Chi (SCHM 3680^H^)	*Pycnidia* of eustroma, triangle, tuberous or irregular, unilocular to multilocular, with darker and thicker wall at the base, 185–425 × 100–125 μm	*Conidiophores* hyaline, septate, branched, 12.5–29 × 1.7–2.6 μm *Conidiogenous cells* hyaline, phialidic	*Alpha conidia* fusiform, hyaline, aseptate, eguttulate or biguttulate, 6.6–8.2 × 1.5–1.8 μm *Beta conidia* filiform, curved or hamate, hyaline, aseptate, 13.5–26.5 × 0.6–0.9 μm G*amma conidia* not observed	[26,106]
*Diaporthe podocarpi-* *-macrophylli* Y.H. Gao & L. Cai * (CGMCC 3.18281^T^)	*Pycnidia* subglobose, dark brown to black, exuding yellowish translucent conidial droplets from ostiole, 250–699 μm diam.	*Alpha conidiophores* cylindrical, straight to sinuous, sometimes inflated, hyaline, septate, branched, in dense clusters, 6–18 × 1.5–3 μm ($\overset{-}{x}$ = 12.3 × 2.1 μm, n = 30) *Beta conidiophores* cylindrical to clavate, straight, hyaline, septate, branched, smooth, 10.5–27 × 1.5–2.5 μm ($\overset{-}{x}$ = 15.3 × 2.1 μm, n = 30)	*Alpha conidia* fusiform, acute ends, hyaline, aseptate, biguttulate, 3.5–8.5 × 1–3 μm ($\overset{-}{x}$ = 6.3 × 2.1 μm, n = 50) *Beta conidia* filiform, curved, tapering towards ends, truncate base, hyaline, aseptate, eguttulate, 8.5–31.5 × 0.5–2 μm ($\overset{-}{x}$ = 19.5 × 1.1 μm, n = 30) *Gamma conidia* not observed	[26]
*Diaporthe pseudomangiferae* R.R. Gomes, Glienke & Crous * (CBS 101339^T^)	*Pycnidia* globose, with elongated necks with central ostioles, exuding yellow–orange to cream conidial droplets, up to 300 μm diam.	*Conidiophores* cylindrical, straight to sinuous, hyaline, 1–3-septate, smooth, branched, densely aggregated, 20–30 × 2–2.5 μm *Conidiogenous cells* cylindrical, terminal and lateral, slightly tapering towards apex, phialidic, with flared collarette (up to 3 μm long), 10–15 × 2–3 μm *Paraphyses* cylindrical, straight to flexuous, hyaline, septate, smooth, unbranched or branched at base, extending above conidiophores, up to 80 μm long and 2–3 μm wide at base	*Alpha conidia* fusiform, tapering towards ends, acutely rounded apex, truncate base, hyaline, aseptate, smooth, guttulate to granular, (6–)7–9(–10) × (2–)2.5(–3) μm *Beta* and *gamma conidia* not observed	[24]
*Diaporthe pseudooculi* Mochiz. and Kaz. Tanaka * (MAFF 246452^T^)	*Pycnidia* globose to depressed globose, with cylindrical to papillate, central ostiolar neck (100–220 × 45–130 μm diam.), exuding white to yellow conidial mass, 220–330 × 180–280 μm diam.	*Conidiophores* hyaline, 5–12 × 2–5 μm *Conidiogeneous cells* cylindrical, phialidic, 12–18 × 2 μm *Paraphyses* filamentous, 50–65 × 1.5–2.5 μm	*Alpha conidia* ellipsoid, hyaline, aseptate, 6–9 × 2–3.5 μm ($\overset{-}{x}$ = 7.3–2.8 μm, L/W = 2.1–3.2, n = 50) *Beta conidia* sigmoid, hyaline, aseptate, 21.5–33.5 × 1.2–1.7 μm ($\overset{-}{x}$ = 27–1.4 μm, n = 30) *Gamma conidia* not observed	[42]
*Diaporthe pseudophoenicicola* R.R. Gomes, C. Glienke & Crous * (CBS 462.69^T^)	*Pycnidia* globose, with neck, exuding yellow-orange conidial droplets through ostiole, up to 400 μm diam.	*Conidiophores* cylindrical, straight to curved, hyaline, 1–3-septate, smooth, branched, densely aggregated, 12–45 × 1.5–3 μm *Conidiogenous cells* cylindrical, terminal and lateral, slightly tapering towards apex, phialidic (with periclinal thickening), with collarette flared (2–5 μm long), 8–15 × 1.5–2.5 μm *Paraphyses* cylindrical, hyaline, 1–3-septate, smoooth, straigh to flexuous, extending above conidiophores, up to 100 μm long and 3 μm wide at base	*Alpha conidia* fusiform, tapering towards ends, straight, acutely rounded apex, truncate base, hyaline, aseptate, smooth, granular, (6–)7–8(–9) × (2–)2.5(–3) μm *Beta* and *gamma conidia* not observed	[24]
*Diaporthe pterocarpicola* Udayanga, Xing Z. Liu and K.D. Hyde * (MFLUCC 10-0580a^T^)	*Pycnidia* hemi-spherical, with slightly elongated black neck, exuding yellowish translucent conidial droplets from ostiole, up to 75 × 120 μm	*Conidiophores* subcylindrical to cylindrical, wide at base, straight to sinuous, hyaline, unbranched, densely aggregated, 7–18 × 1.5–3.5 μm, 2.5–3.5 wide at base *Conidiogenous cells* cylindrical, terminal, slightly tapering towards apex, phialidic (with periclinal thickening), 1–2 μm diam. *Paraphyses* occasionally present, cylindrical, straight to flexuous, hyaline, septate, smooth, unbranched, extending above conidiophores, up to 25 μm long and 1.5–2 μm wide at base	*Alpha conidia* ellipsoid or clavate, subtruncate base, hyaline, aseptate, multiguttulate, (5–)6–7(–8) × (2–)2.5(–3.5) μm *Beta* and *gamma conidia* not observed	[31]
*Diaporthe schimae* C.M. Tian and Q. Yang * (CFCC 53103^T^)	*Pycnidia* globose, exuding cream to yellowish translucent conidial droplets from ostiole, (150–)180–300(–373) μm diam.	*Conidiophores* reduced to conidiogeneous cells. *Conidiogenous cells* straight, slightly tapering towards apex, hyaline, septate, unbranched	*Alpha conidia* scarce, ellipsoidal to spindle-shaped, hyaline, aseptate, 4-guttulate, (7.5–)8–8.5(–9) × 2.5–3 μm *Beta conidia* filiform, straight to sinuous at one end, hyaline, aseptate, eguttulate, (25–)27.5–38.5(–40.5) × 1–1.5 µm *Gamma conidia* not observed	[121]
*Diaporthe searlei* R.G. Shivas, Akinsanmi & Y.P. Tan * (BRIP 66528^T^)	*Pycnidia* globose or irregular, dark brown to black, up to 1 mm diam.	*Conidiophores* hyaline, smooth, densely aggregated, 15–45 μm long *Conidiogeneous cells* cylindrical, straight or flexuous, hyaline, phialidic, 10–35 × 1–2.5 μm	*Alpha conidia* fusiform, acute ends, hyaline, aseptate, 5–9 × 1.5–2 μm *Beta* and *gamma conidia* not observed	[112]
*Diaporthe sennae* C.M. Tian and Qin Yang * (CFCC 51636^T^)	*Pycnidia* circular to ovoid, uniloculate and undivided, ectostromatic disc brown to dark, with one ostiole, (400–)500–600(–680) μm ($\overset{-}{x}$ = 570 μm, n = 20) diam.	*Conidiophores* reduced to conidiogeneous cells. *Conidiogenous cells* straight or slightly curved, hyaline, phialidic	*Alpha conidia* ellipsoidal to oval, hyaline, aseptate, smooth, biguttulate, rarely 3-guttulate, (5–)5.5–6.3(–6.5) × 1.5–1.7(–1.8) μm ($\overset{-}{x}$ = 6 × 1.6 μm, n = 50) *Beta conidia* straight to hamate, hyaline, aseptate, smooth, (17.3–)18.4–20(–23.3) × 0.9 μm ($\overset{-}{x}$ = 19.1 × 0.9 μm, n = 50) *Gamma conidia* not observed	[122]
*Diaporthe spinosa* Y.S. Guo and G.P. Wang * (CGMCC 3.19602^T^)	*Pycnidia* globose, dark brown to black, 124–172 μm diam.	*Conidiophores* ampulliform, hyaline, 1-septate, smooth, unbranched, densely aggregated, 6–9 × 3–4.5 μm *Conidiogeneous cells* cylindrical, straight, terminal, tapering towards apex, hyaline, 8–29 × 1.5–2.5 μm	*Alpha conidia* fusiform to oval, acutely rounded ends, hyaline, aseptate, bi- or multi-guttulate, 5.5–8 × 2–3.5 μm ($\overset{-}{x}$ = 7 × 2.6 μm, n = 50; L/W = 2.7) *Beta conidia* filiform, curved, tapering towards ends, multi-guttulate, 18.5–30.5 × 1–1.5 μm ($\overset{-}{x}$ = 25.1 × 1.3 μm, n = 38; L/W = 19.3) *Gamma conidia* not observed	[104]
*Diaporthe taiwanensis* H.A. Ariyaw. and I. Tsai * (NTUCC 18-105-1^T^)	*Pycnidia* irregular, with hairy neck, exuding yellowish conidial droplets, up to 270 μm diam.	*Conidiophores* cylindrical, hyaline, septate, branched, 11–15 × 1–2.5 μm *Conidiogenous cells* subcylindrical, straight to curved, tapering towards apex, hyaline, 7–8.5 × 1–2.5 μm	*Alpha conidia* fusiform, acute ends, hyaline, aseptate, 1–3-guttulate, 7–9.5 × 2.5–3 μm *Beta conidia* acutely rounded and curved apex, hyaline, smooth, 24–30 × 1–2 μm *Gamma conidia* not observed	[123]
*Diaporthe taoicola* Dissanayake, X.H. Li & K.D. Hyde * (MFLUCC 16-0117^T^)	*Pycnidia* globose, black, multilocular, exuding cream conidial droplets from central ostiole, up to 300 μm diam.	*Conidiophores* cylindrical, straight to sinuous, hyaline, smooth, densely aggregated, 10–25 × 2–3 μm *Conidiogenous cells* cylindrical, terminal and lateral, slightly tapering towards apex, phialidic, 9–16 × 1.5–2 μm *Paraphyses* cylindrical, with obtuse ends, hyaline, 1–3-septate, smooth, extending above conidiophores	*Alpha conidia* fusoid to ellipsoid, subobtuse apex, bluntly rounded base with flattened hilum, tapering towards ends, straight, hyaline, smooth, guttulate, 7–9 × 2–3 μm ($\overset{-}{x}$ = 8 × 3 μm) *Beta conidia* spindle-shaped, curved, tapering towards subacutely rounded apex, truncate base, hyaline, aseptate, 20–25 × 1.5–2 μm ($\overset{-}{x}$ = 19 × 2 μm) *Gamma conidia* not observed	[41]
*Diaporthe viciae* W.S. Zhao, Q. Ning and J.Y. Yan * (JZB 320179^T^)	*Pycnidia* oval to round, black, 150–200 × 150–250 μm	*Conidiophores* cylindrical, aseptate, densely aggregated, 15–32.5 μm long *Conidiogenous cells* cylindrical, terminal and lateral, phialidic	*Alpha conidia* fusiform or oval, hyaline, 2–5-guttulate, 7–10 × 2–4 μm ($\overset{-}{x}$ = 8.3 × 3 μm, n = 50) *Beta* and *gamma conidia* not observed	[124]
*Diaporthe viniferae* Dissanayake, X.H. Li & K.D. Hyde * (JZB 320071^T^)	*Pycnidia* globose, dark brown to black, 363–937 μm ($\overset{-}{x}$ = 529 μm, n = 20) diam.	*Conidiophores* not observed *Conidiogenous cells* not observed	*Alpha conidia* fusiform or oval, obtuse ends, hyaline, bi-guttulate, 5–8.3 × 1.3–2.5 μm ($\overset{-}{x}$ = 6.4 × 2.1 μm) *Beta conidia* filiform, hamate, tapering towards ends, hyaline, aseptate, 23–35 × 1–1.5 μm ($\overset{-}{x}$ = 28 × 1.3 μm, n = 40) *Gamma conidia* not observed	[21]

^1^ Species synonymized in the present study under *Diaporthe arecae* are noted with a superscript asterisk (*); status of the strains or specimens are noted by superscript H (holotype), IH (isotype), IT (ex-isotype), R (reference) and T (ex-type); ^2^ N/A = not available, i.e., feature not mentioned by the respective authors in the taxonomic description of the species; Note: seven species synonymized in the present study under *Diaporthe arecae* were excluded from this synopsis due to lack of morphological data regarding their asexual morphs. *Diaporthe annellsiae* Y.P. Tan and R.G. Shivas, *Diaporthe bounty* Y.P. Tan and R.G. Shivas, *Diaporthe gossiae* Y.P. Tan and R.G. Shivas, *Diaporthe howardiae* Y.P. Tan & R.G. Shivas and *Diaporthe norfolkensis* Y.P. Tan & R.G. Shivas were introduced by Tan and Shivas [125] based on the diagnosis of sequence data obtained apparently from the type specimens and no taxonomic descriptions were provided. *Diaporthe hongheensis* E.F. Yang and Tibpromma (KUMCC 21-0457^T^) was introduced by Yang et al. [126] based on morpho-molecular analyses, but only the sexual morph was observed on the host tissue, and no sporulation was observed in culture. *Diaporthe pandanicola* Tibpromma and K.D. Hyde was introduced by Tibpromma et al. [127] based on the diagnosis of sequence data, since no sporulation was observed in culture.

**Table 6:** Summary of host, country and ecological group for all type specimens proposed as synonymous to *Diaporthe arecae*.

Taxon ^1^	Host	Country	Ecological Group ^2^	Reference
***Diaporthe arecae***	Fruit of *Areca catechu* (*Arecaceae*)	India	Potential pathogen	[38]
*Diaporthe acuta* *	Diseased branches of *Pyrus pyrifolia* (*Rosaceae*)	China (Hubei)	Pathogen	[104]
*Diaporthe annellsiae* *	Fruit of *Mangifera indica* (*Anacardiaceae*)	Australia (Western Australia)	UN	[125]
*Diaporthe anhuiensis* *	Leaves of *Cunninghamia lanceolata* (*Cupressaceae*)	China (Anhui)	Endophyte	[105]
***Diaporthe arengae*** *	*Arenga engleri* (*Arecaceae*)	China (Hong Kong)	UN	[24]
*Diaporthe averrhoae* *	Branches of *Averrhoa carambola* (*Oxalidaceae*)	China (Fujian)	UN	[106]
*Diaporthe bounty* *	Leaf spots of *Malus domestica* (*Rosaceae*)	Australia (Norfolk Island)	Potential pathogen	[125]
*Diaporthe camelliae-oleiferae* *	Leaf spots of *Camellia oleifera* (*Theaceae*)	China (Hunan)	Potential pathogen	[107]
*Diaporthe ceratozamiae* *	Leaf spots of *Ceratozamia robusta* (*Zamiaceae*)	Australia (Queensland)	Potential pathogen	[108]
*Diaporthe cercidis* *	Twigs and branches of *Cercis chinensis* (*Fabaceae*)	China (Jiangsu)	UN	[109]
***Diaporthe chamaeropicola*** *	Leaf spots of *Chamaerops humilis* (*Arecaceae*)	Portugal (Lisbon)	Potential pathogen	[64]
***Diaporthe chrysalidocarpi*** *	Leaf spots of *Chrysalidocarpus lutescens* (*Arecaceae*)	China (Yunnan)	Potential pathogen	[110]
*Diaporthe delonicis* *	Seed pods of *Delonix regia* (*Fabaceae*)	Thailand (Chiang Rai)	Saprophyte	[111]
*Diaporthe drenthii* *	Rotten husk of *Macadamia* sp. (*Proteaceae*)	South Africa (KwaZulu-Natal)	Pathogen	[112]
*Diaporthe endocitricola* *	Fruits of *Citrus grandis* (*Rutaceae*)	China (Guangdong)	Endophyte	[113]
*Diaporthe fraxini-angustifoliae* *	Diseased stems of *Fraxinus angustifolia* (*Oleaceae*)	Australia (Victoria)	Potential pathogen	[39]
*Diaporthe fulvicolor* *	Diseased branches of *Pyrus pyrifolia* (*Rosaceae*)	China (Hubei)	Pathogen	[104]
*Diaporthe gossiae* *	Stem of *Sesbania* sp. (*Fabaceae*)	Australia (Western Australia)	UN	[125]
*Diaporthe guangxiensis* *	Diseased trunk of *Vitis vinifera* (*Vitaceae*)	China (Guangxi)	Pathogen	[21]
*Diaporthe hongheensis* *	Branch of *Mangifera indica* (*Anacardiaceae*)	China (Yunnan)	Saprophyte	[126]
*Diaporthe howardiae* *	Leaf spots of *Agave* sp. (*Asparagaceae*)	Australia (Norfolk Island)	Potential pathogen	[125]
*Diaporthe huangshanensis* *	Leaves of *Camellia oleifera* (*Theaceae*)	China (Anhui)	Endophyte	[105]
*Diaporthe hunanensis* *	Leaf spots of *Camellia oleifera* (*Theaceae*)	China (Hunan)	Potential pathogen	[107]
*Diaporthe krabiensis* *	Submerged wood of *Bruguiera* sp. (*Rhizophoraceae*)	Thailand (Krabi)	Saprophyte	[114]
*Diaporthe limonicola* *	Branch canker of *Citrus limon* (*Rutaceae*)	Malta (Gozo)	Pathogen	[10]
*Diaporthe liquidambaris* *	Branches of *Liquidambar formosana* (*Altingiaceae*)	China (Fujian)	UN	[106]
*Diaporthe litchiicola* *	Diseased *Litchi chinensis* (*Sapindaceae*)	Australia (Queensland)	Potential pathogen	[39]
*Diaporthe loropetali* *	Branches of *Loropetalum chinense* (*Hamamelidaceae*)	China (Hunan)	UN	[116]
*Diaporthe meliae* *	Branche canker of *Melia azedarach* (*Meliaceae*)	China (Shandong)	Potential pathogen	[117]
*Diaporthe melitensis* *	Branch canker of *Citrus limon* (*Rutaceae*)	Malta (Gozo)	Pathogen	[10]
*Diaporthe millettiae* *	Leaves of *Millettia reticulata* (*Fabaceae*)	China (Guangxi)	UN	[118]
*Diaporthe musigena* *	Necrotic leaves of *Musa* sp. (*Musaceae*)	Australia (Queensland)	Potential pathogen	[119]
*Diaporthe nelumbonis* *	Leaf spots of *Nelumbo nucifera* (*Nelumbonaceae*)	China (Taiwan, Taipei)	Potential pathogen	[120]
*Diaporthe norfolkensis* *	Panicle of *Mangifera indica* (*Anacardiaceae*)	Australia (Norfolk Island)	UN	[125]
*Diaporthe oculi* *	Diseased human eye	Japan (Gifu)	Pathogen	[42]
*Diaporthe osmanthi* *	Leaves of *Osmanthus fragrans* (*Oleaceae*)	China (Guangxi)	UN	[118]
*Diaporthe pandanicola* *	Leaves of *Pandanus* sp. (*Pandanaceae*)	Thailand (Chumphon)	Endophyte	[127]
*Diaporthe pascoei* *	Roten fruit of *Persea Americana* (*Lauraceae*)	Australia (Victoria)	Potential pathogen	[39]
*Diaporthe pescicola* *	Shoots of *Prunus persica* (*Rosaceae*)	China (Hubei)	Pathogen	[41]
*Diaporthe phyllanthicola* *	Branches of *Phyllanthus emblica* (*Phyllanthaceae*)	China (Fujian)	UN	[106]
*Diaporthe podocarpi-macrophylli* *	Leaves of *Podocarpus macrophyllus* (*Podocarpaceae*)	Japan	UN	[26]
*Diaporthe pseudomangiferae* *	*Mangifera indica* (*Anacardiaceae*)	Dominican Republic	UN	[24]
*Diaporthe pseudooculi* *	Diseased human eye	Japan (Gifu)	Pathogen	[42]
***Diaporthe pseudophoenicicola*** *	Dead tops of green leaves on *Phoenix dactylifera* (*Arecaceae*)	Spain (Mallorca)	UN	[24]
*Diaporthe pterocarpicola* *	Leaf spot of *Pterocarpus indicus* (Fabaceae)	Thailand (Chiang Rai)	Potential pathogen	[31]
*Diaporthe schimae* *	Leaf spots of *Schima superba* (*Theaceae*)	China (Jiangxi)	Potential pathogen	[121]
*Diaporthe searlei* *	Rotten husk of *Macadamia* sp. (*Proteaceae*)	South Africa (Mpumalanga)	Pathogen	[112]
*Diaporthe sennae* *	Diseased twigs and branches of *Senna bicapsularis* (*Fabaceae*)	China (Guangxi)	Potential pathogen	[122]
*Diaporthe spinosa* *	Diseased branches of *Pyrus pyrifolia* (*Rosaceae*)	China (Jiangsu)	Pathogen	[104]
*Diaporthe taiwanensis* *	Leaf spots of *Ixora chinensis* (*Rubiaceae*)	China (Taiwan, Taoyuan)	Pathogen	[123]
*Diaporthe taoicola* *	Shoots of *Prunus persica* (*Rosaceae*)	China (Hubei)	Pathogen	[41]
*Diaporthe viciae* *	Stems of *Vicia villosa* (*Fabaceae*)	China (Guangxi)	Endophyte	[124]
*Diaporthe viniferae* *	Diseased trunk of *Vitis vinifera* (*Vitaceae*)	China (Guangxi)	Pathogen	[21]

^1^ Species synonymized in the present study under *Diaporthe arecae* are noted with a superscript asterisk (*); species originally described from *Arecaceae* hosts are highlighted in bold; ^2^ UN: unknown, information not mentioned by the respective authors; the ecological group “potential pathogen” stands for those species recovered from symptomatic tissues, but for which pathogenicity tests were not conducted to prove their pathogenicity.

## Data Availability

All data generated or analysed in this study are included in this article and its supplementary information files. All sequence data are available in the NCBI GenBank, following the accession numbers in the manuscript.

## Supplementary Materials

The following supporting information can be downloaded at: https://www.mdpi.com/article/10.3390/microorganisms11112717/s1, Figures S1–S5: Phylogenetic trees generated from maximum likelihood analyses based on ITS, *tef1*, *tub2*, *cal* and *his3* sequence data, respectively, for all species of the *Diaporthe arecae* species complex and related species. Figures S6–S8: Phylogenetic trees generated from maximum likelihood analysis for species of the *Diaporthe arecae* species complex and related species with available sequence data of ITS, *tef1*, *tub2*, *cal* and *his3* (5-loci), ITS, *tef1*, *tub2* and *cal* (4-loci) and ITS, *tef1* and *tub2* (3-loci), respectively. Figure S9: Maximum likelihood species delimitation scheme obtained from the multi-rate Poisson tree process (mPTP) analysis of the *Diaporthe arecae* species complex and related species, based on combined dataset of 2-loci (ITS and *tef1*).
