# Supplementary material for: Diaporthe Species on Palms: Molecular Re-Assessment and Species Boundaries Delimitation in the D. arecae Species Complex
Source: Microorganisms. 2023 Nov 6;11(11):2717. doi: 10.3390/microorganisms11112717 (PMC10673533; doi:10.3390/microorganisms11112717)
Supplement: Supplementary file 1 [file microorganisms-11-02717-s001.zip › microorganisms-2663415-supplementary.pdf]

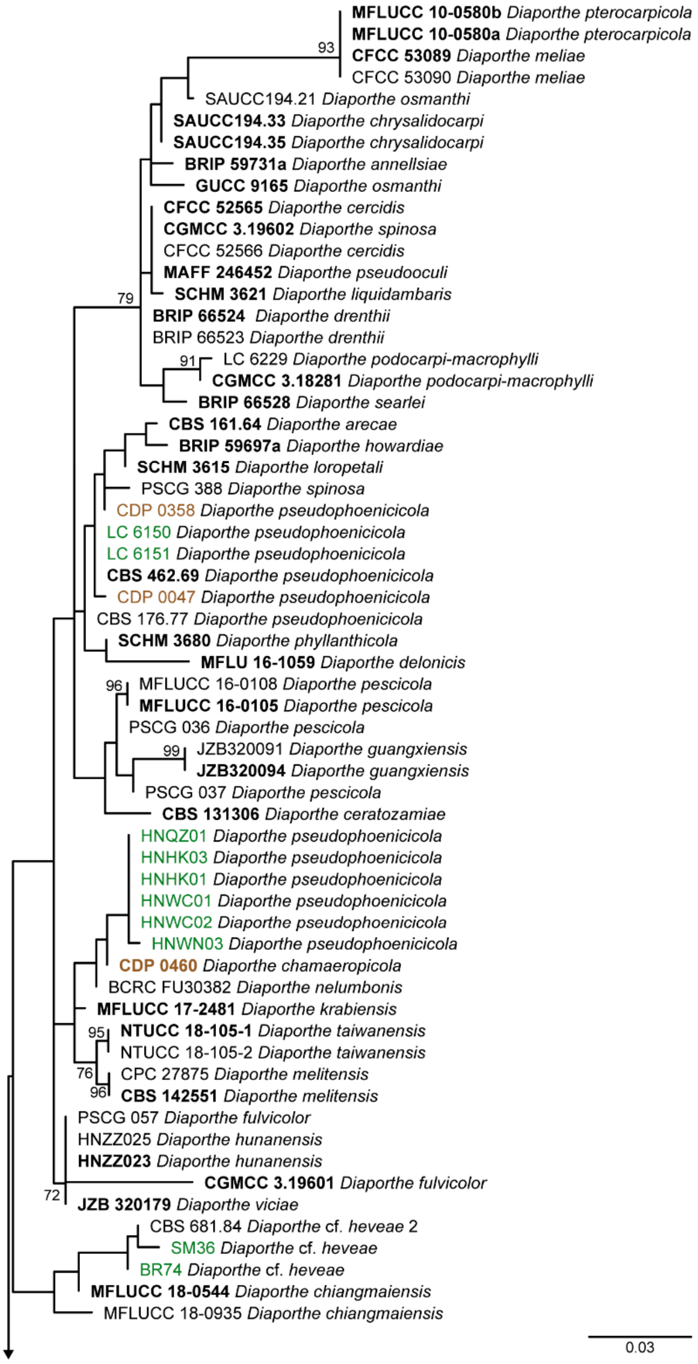

Figure S1. Continued.

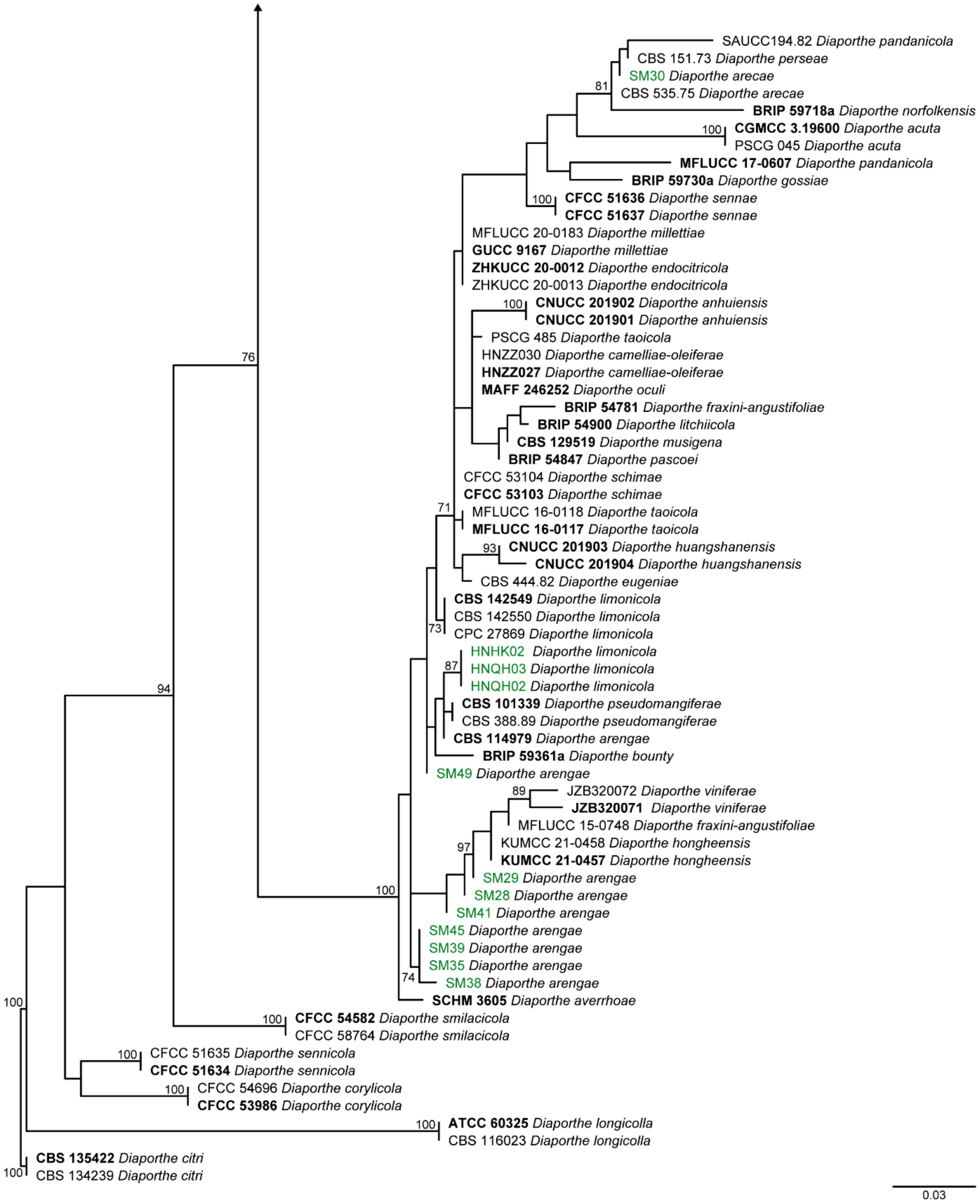

Figure S1. Phylogenetic tree generated from maximum likelihood analysis based on ITS sequence data of the *Diaporthe arecae* species complex and related species. Bootstrap support values for maximum likelihood (ML-BS ≥ 70 %) are shown at the nodes. Strains with type status are indicated in bold font. The isolates from this study are presented in brown typeface and the additional isolates from palm tissues included in the analyses are presented in green typeface. The scale bar represents the expected number of nucleotide changes per site. The tree is rooted to *Diaporthe citri* (CBS 134239 and CBS 135422).

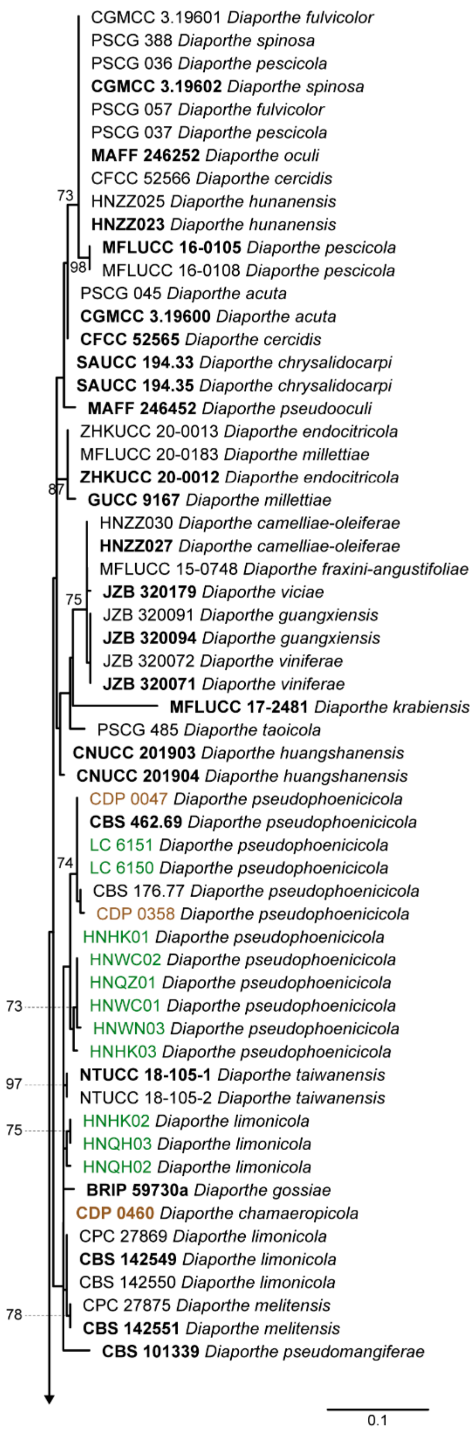

Figure S2. Continued.

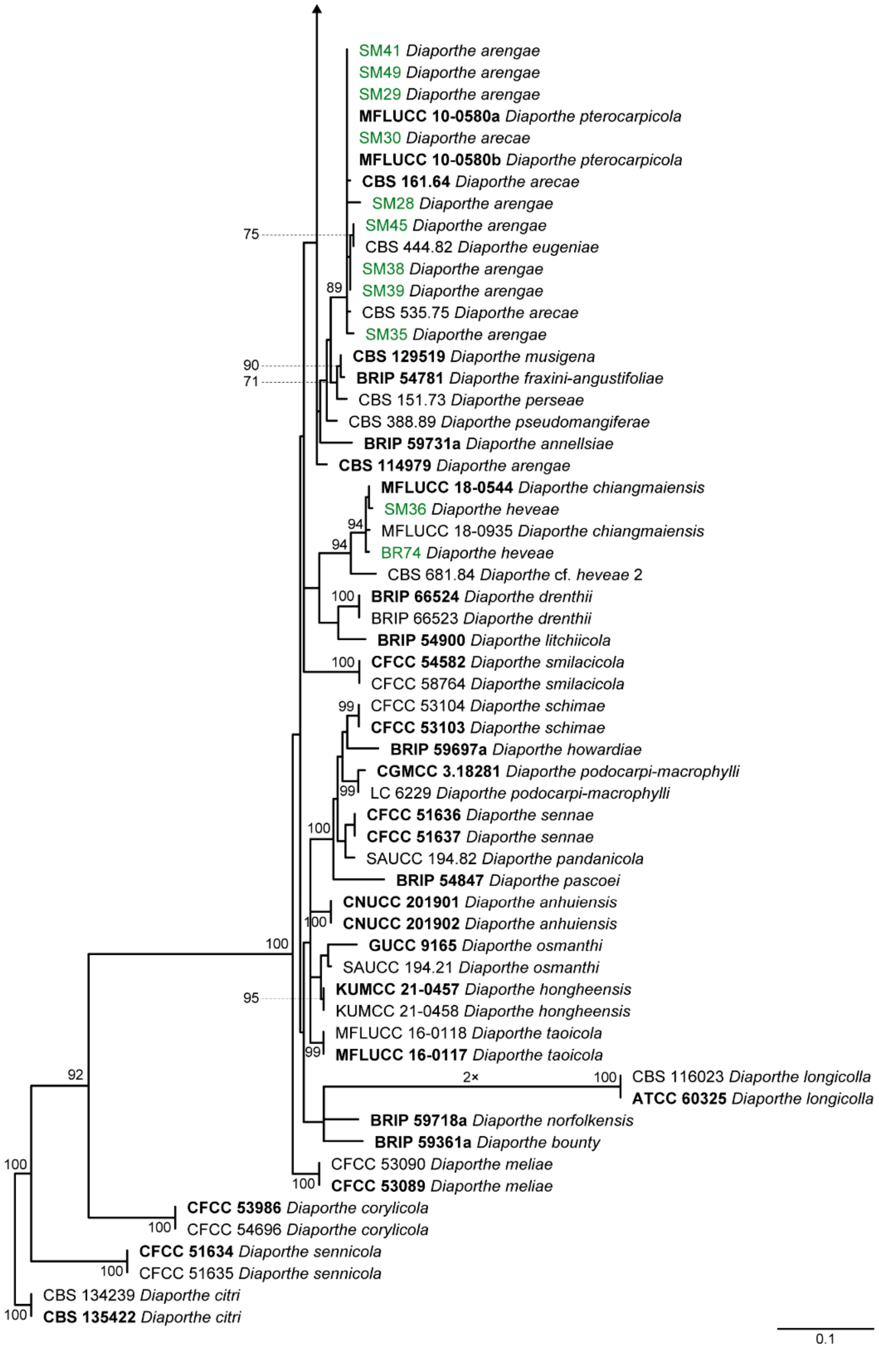

Figure S2. Phylogenetic tree generated from maximum likelihood analysis based on *tef1* sequence data of the *Diaporthe arecae* species complex and related species. Bootstrap support values for maximum likelihood (ML-BS ≥ 70 %) are shown at the nodes. Strains with type status are indicated in bold font. The isolates from this study are presented in brown typeface and the additional isolates from palm tissues included in the analyses are presented in green typeface. The scale bar represents the expected number of nucleotide changes per site. The tree is rooted to *Diaporthe citri* (CBS 134239 and CBS 135422).

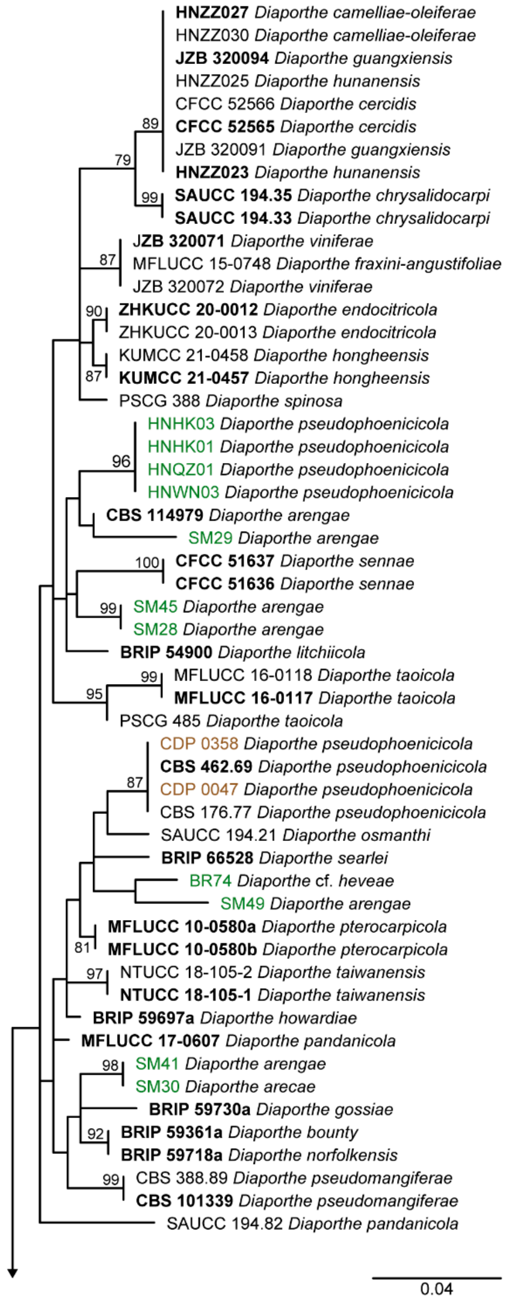

Figure S3. Continued.

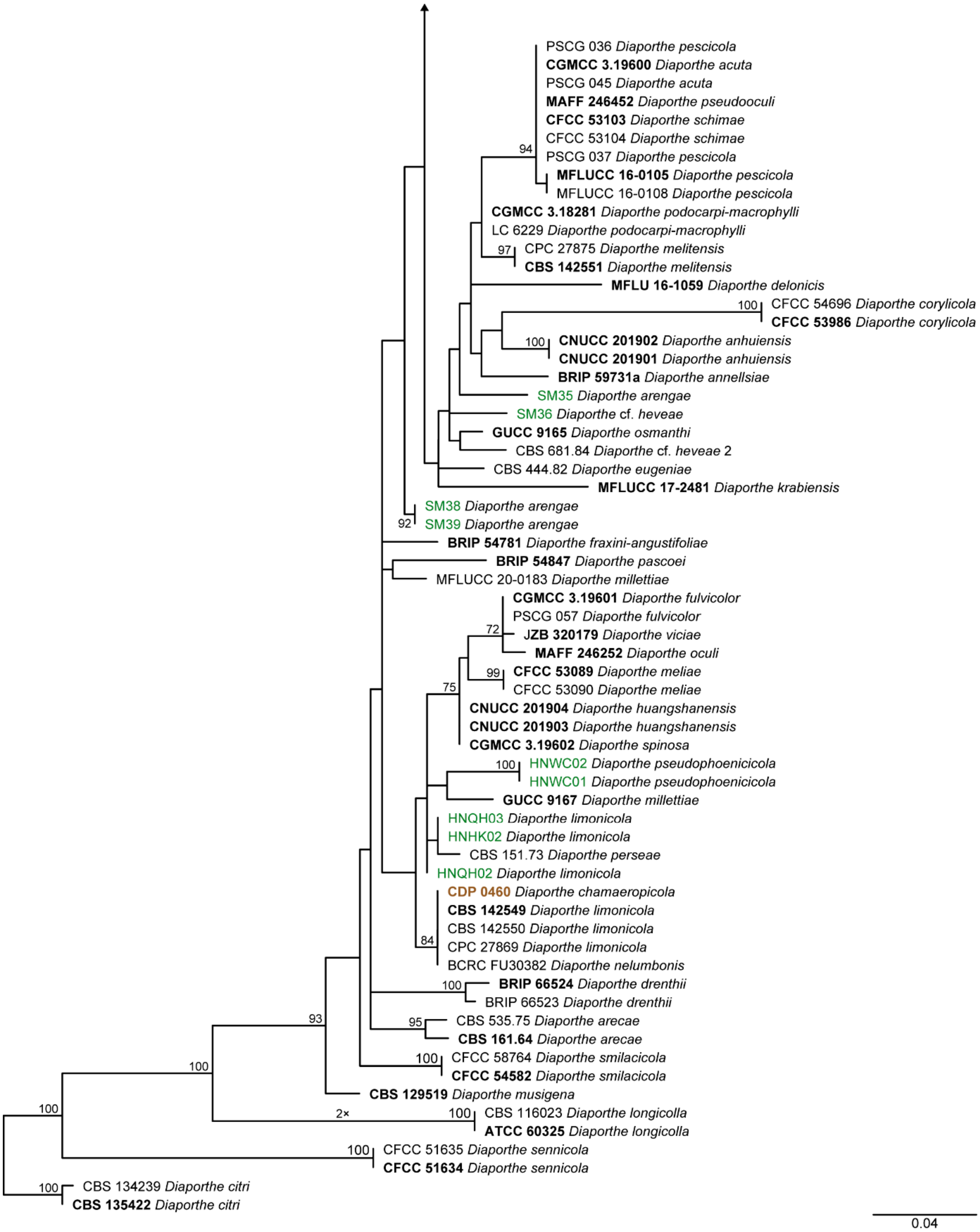

Figure S3. Phylogenetic tree generated from maximum likelihood analysis based on *tub2* sequence data of the *Diaporthe arecae* species complex and related species. Bootstrap support values for maximum likelihood (ML-BS ≥ 70 %) are shown at the nodes. Strains with type status are indicated in bold font. The isolates from this study are presented in brown typeface and the additional isolates from palm tissues included in the analyses are presented in green typeface. The scale bar represents the expected number of nucleotide changes per site. The tree is rooted to *Diaporthe citri* (CBS 134239 and CBS 135422).

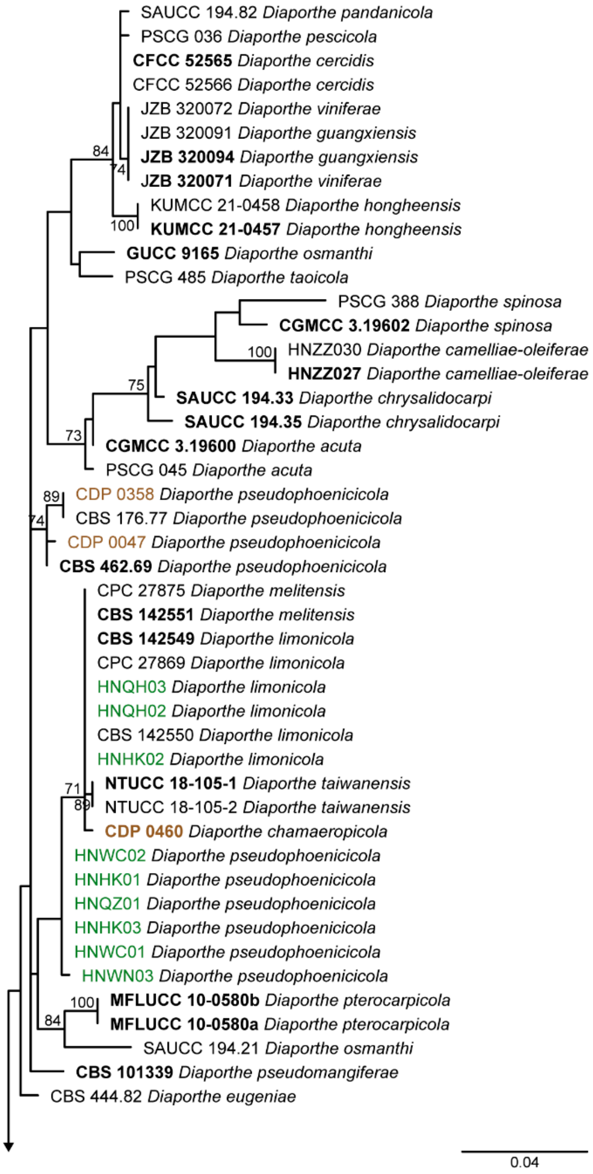

Figure S4. Continued.

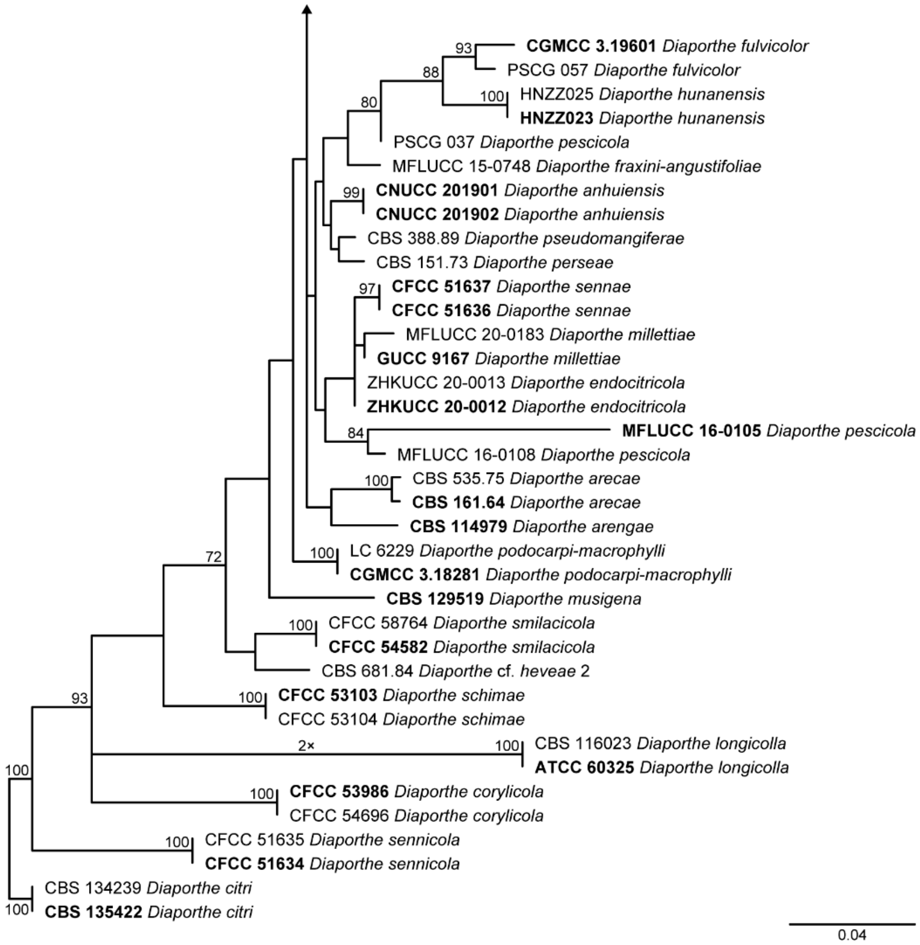

Figure S4. Phylogenetic tree generated from maximum likelihood analysis based on *cal* sequence data of the *Diaporthe arecae* species complex and related species. Bootstrap support values for maximum likelihood (ML-BS ≥ 70 %) are shown at the nodes. Strains with type status are indicated in bold font. The isolates from this study are presented in brown typeface and the additional isolates from palm tissues included in the analyses are presented in green typeface. The scale bar represents the expected number of nucleotide changes per site. The tree is rooted to *Diaporthe citri* (CBS 134239 and CBS 135422).

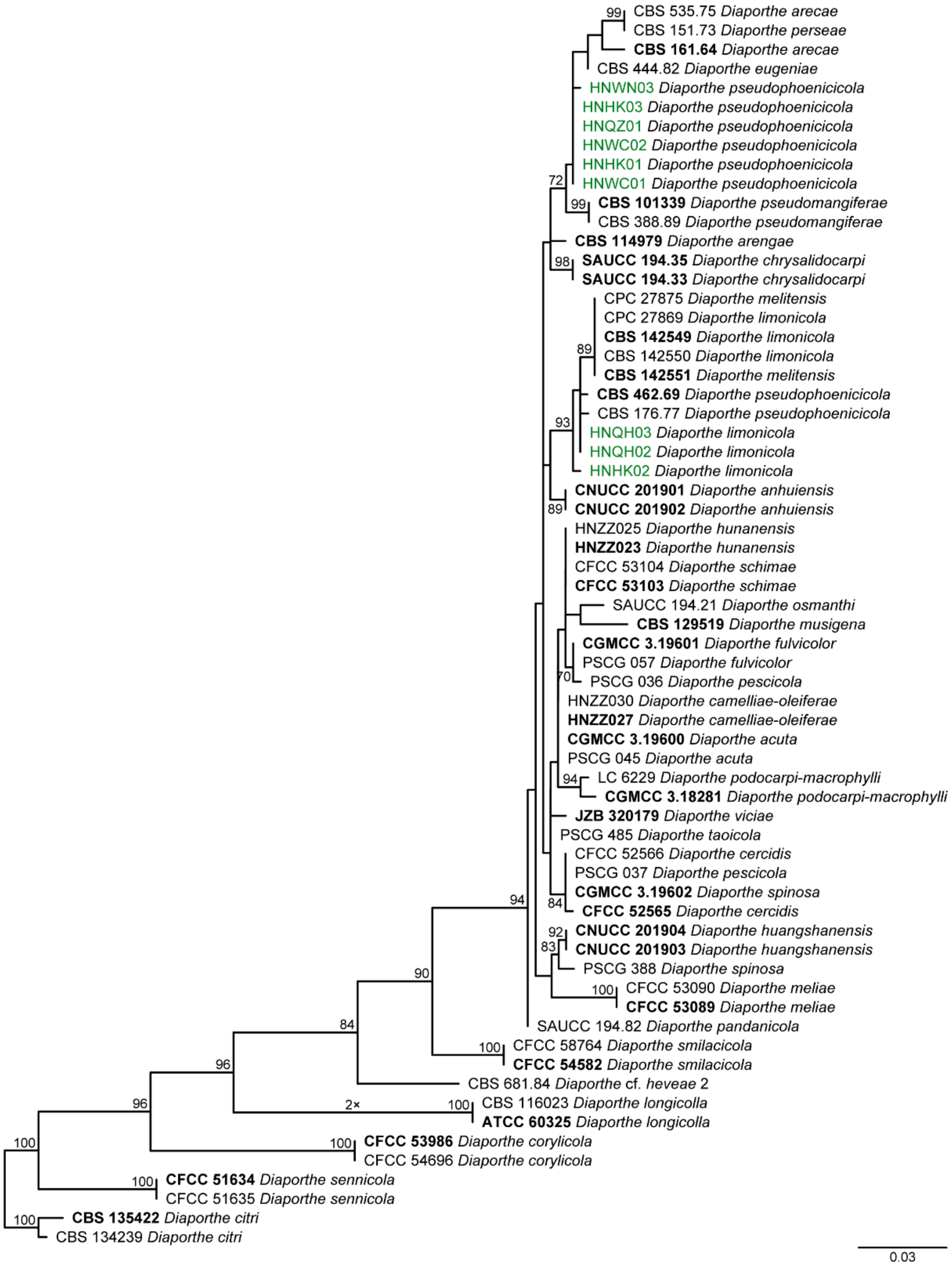

Figure S5. Phylogenetic tree generated from maximum likelihood analysis based on *his3* sequence data of the *Diaporthe arecae* species complex and related species. Bootstrap support values for maximum likelihood (ML-BS ≥ 70 %) are shown at the nodes. Strains with type status are indicated in bold font. The additional isolates from palm tissues included in the analyses are presented in green typeface. The scale bar represents the expected number of nucleotide changes per site. The tree is rooted to *Diaporthe citri* (CBS 134239 and CBS 135422).

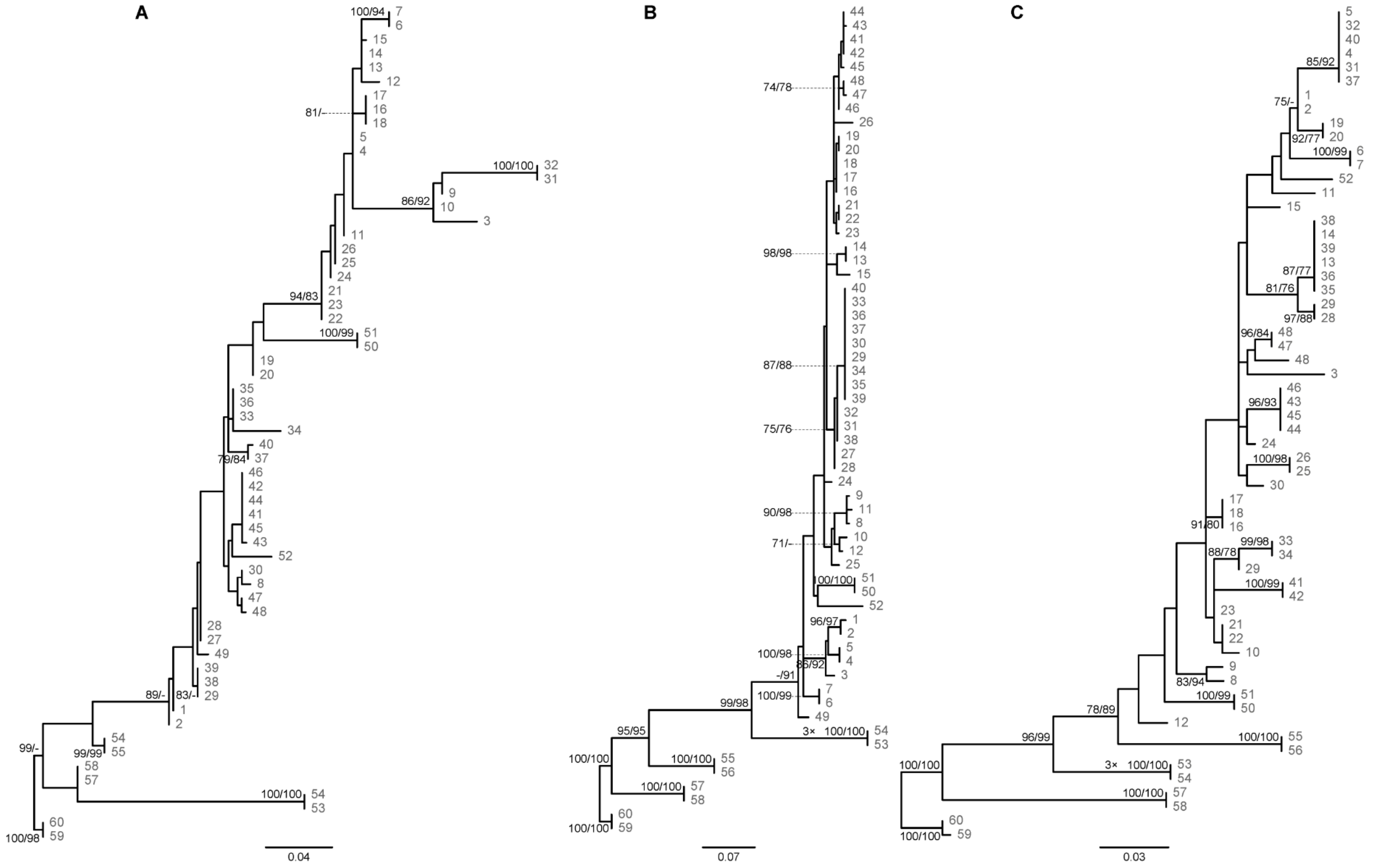

Figure S6. Continued.

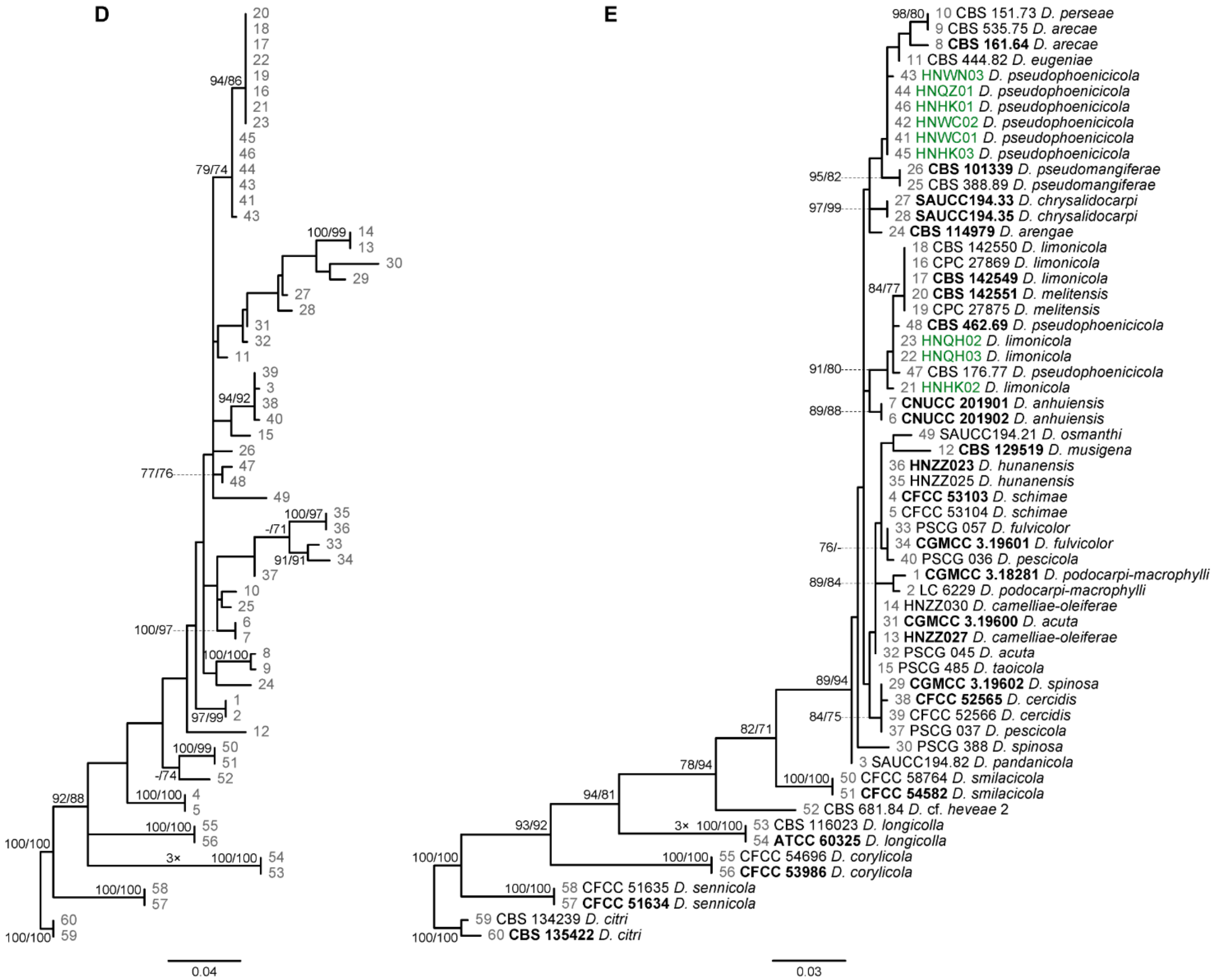

Figure S6. Phylogenetic trees generated from maximum likelihood analysis of the *Diaporthe arecae* species complex and related species. A. Based on ITS sequence data. B. Based on *tef1* sequence data. C. Based on *tub2* sequence data. D. Based on *cal* sequence data. E. Based on *his3* sequence data. Bootstrap support values for maximum likelihood and maximum parsimony (ML-BS/MP-BS ≥ 70 %) are shown at the nodes. Taxa numbers were generated, and the corresponding strains and species are shown in the *his3*-phylogram (panel E). Strains with type status are indicated in bold font. The additional isolates from palm tissues included in the analyses are presented in green typeface. The scale bars represent the expected number of nucleotide changes per site. The trees are rooted to *Diaporthe citri* (CBS 134239 and CBS 135422).

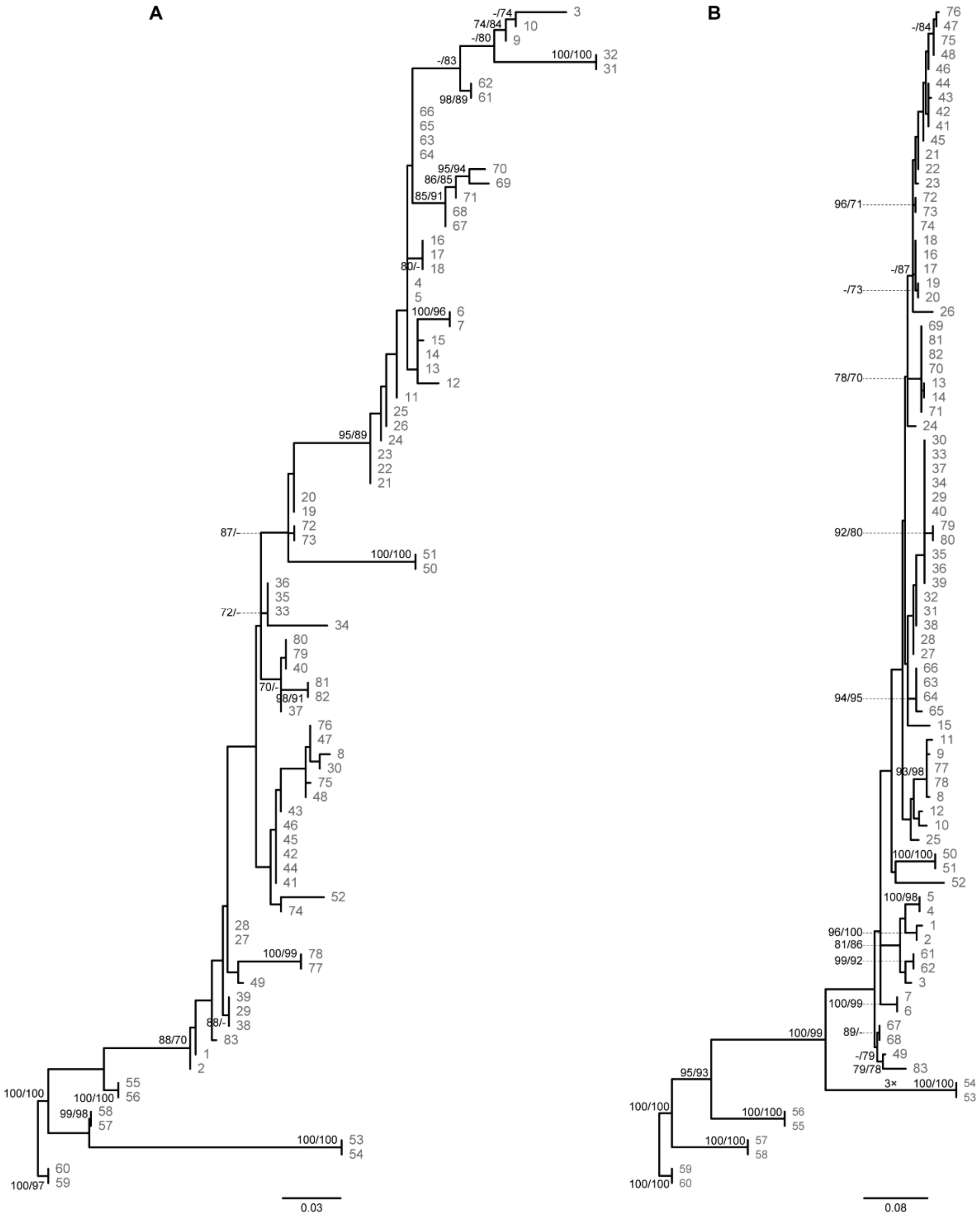

Figure S7. Continued.

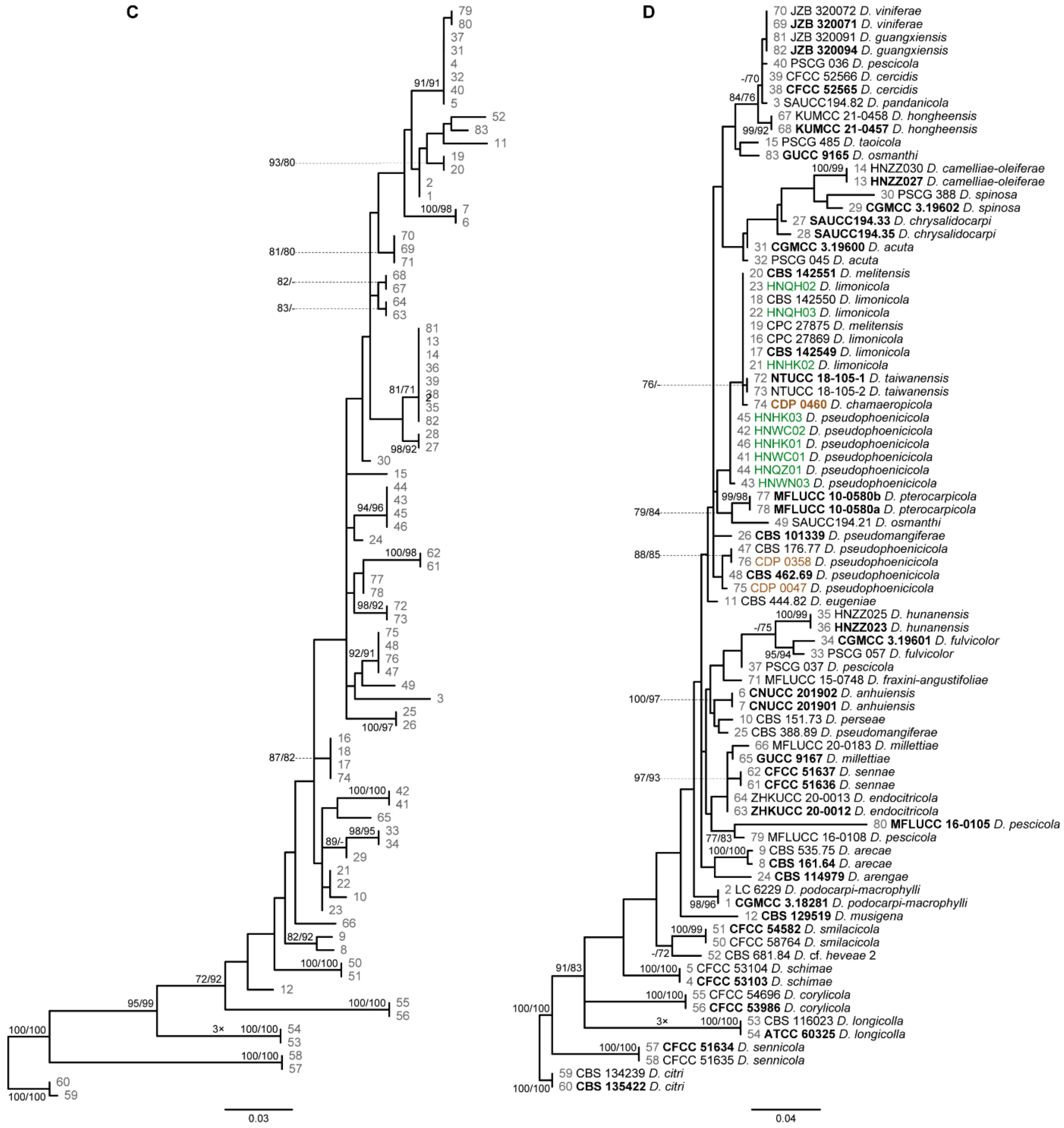

Figure S7. Phylogenetic trees generated from maximum likelihood analysis of the *Diaporthe arecae* species complex and related species. A. Based on ITS sequence data. B. Based on *tef1* sequence data. C. Based on *tub2* sequence data. D. Based on *cal* sequence data. Bootstrap support values for maximum likelihood and maximum parsimony (ML-BS/MP-BS ≥ 70 %) are shown at the nodes. Taxa numbers were generated, and the corresponding strains and species are shown in the *cal*-phylogram (panel D). Strains with type status are indicated in bold font. The isolates from this study are presented in brown typeface and the additional isolates from palm tissues included in the analyses are presented in green typeface. The scale bars represent the expected number of nucleotide changes per site. The trees are rooted to *Diaporthe citri* (CBS 134239 and CBS 135422).

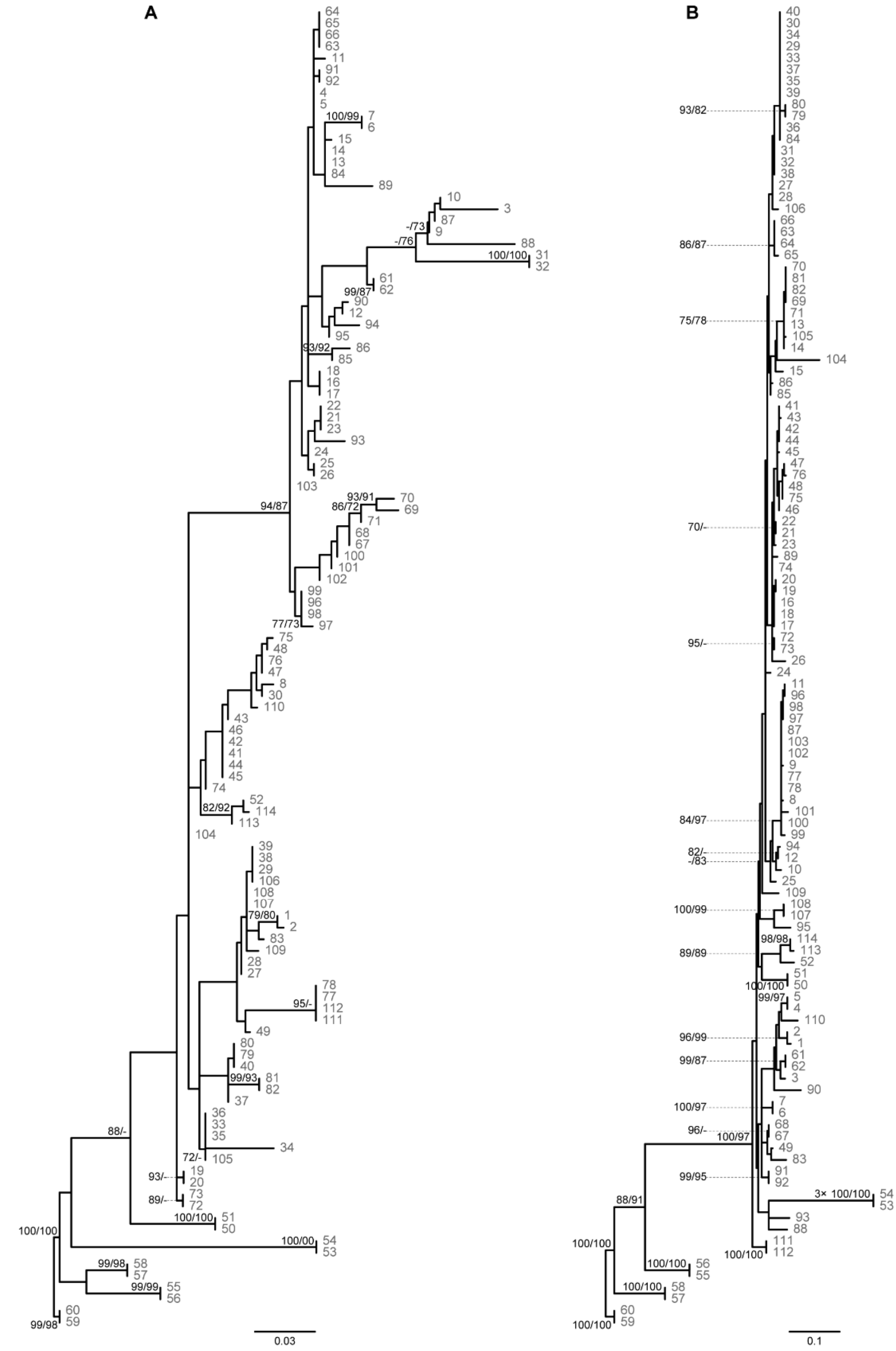

Figure S8. Continued.

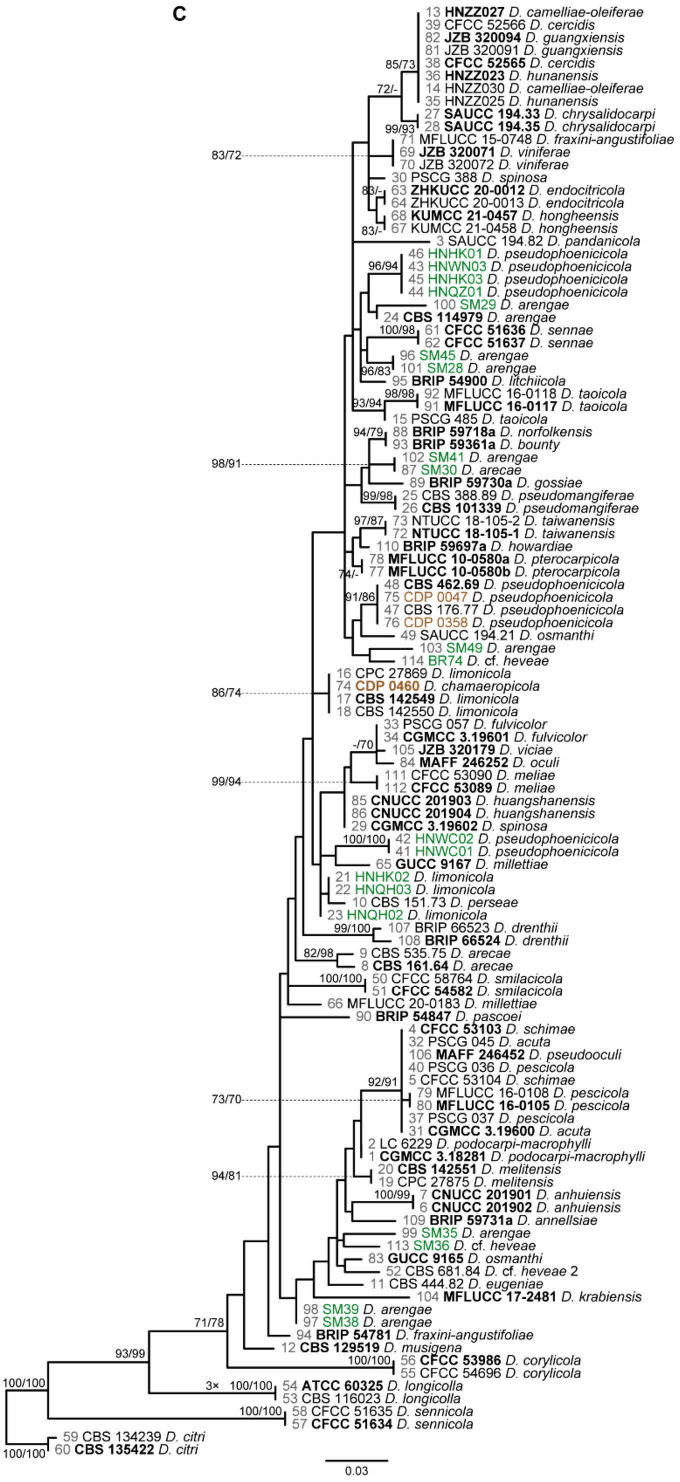

Figure S8. Phylogenetic trees generated from maximum likelihood analysis of the *Diaporthe arecae* species complex and related species. A. Based on ITS sequence data. B. Based on *tef1* sequence data. C. Based on *tub2* sequence data. Bootstrap support values for maximum likelihood and maximum parsimony (ML-BS/MP-BS ≥ 70 %) are shown at the nodes. Taxa numbers were generated, and the corresponding strains and species are shown in the *tub2*-phylogram. Strains with type status are indicated in bold font. The isolates from this study are presented in brown typeface and the additional isolates from palm tissues included in the analyses are presented in green typeface. The scale bars represent the expected number of nucleotide changes per site. The trees are rooted to *Diaporthe citri* (CBS 134239 and CBS 135422).

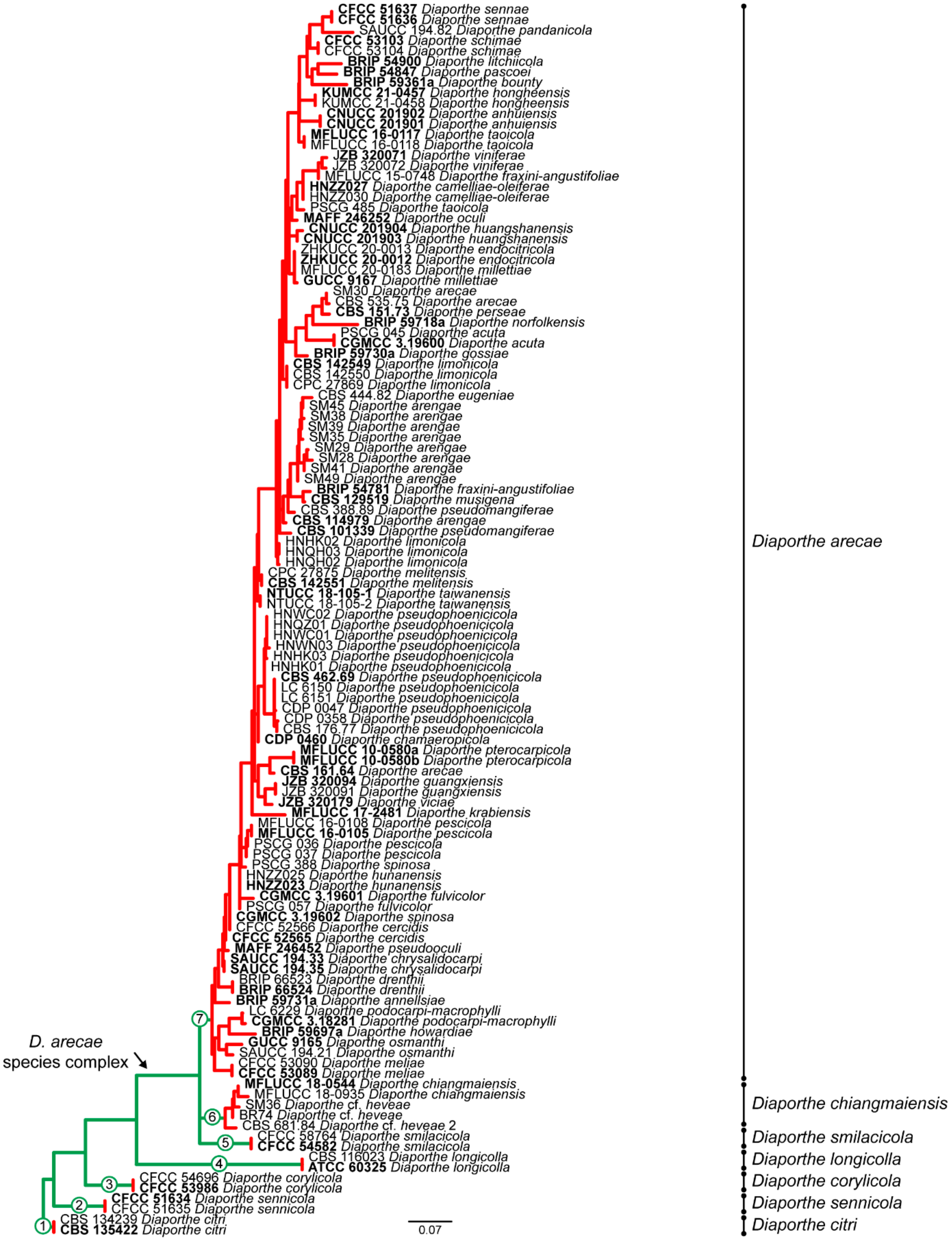

Figure S9. Maximum likelihood species delimitation scheme obtained from the multi-rate Poisson tree process (mPTP) analysis of the *Diaporthe arecae* species complex and related species, based on combined dataset of 2-loci (ITS and *tef1*). Blue-colored branches illustrate the speciation process and red-colored branches illustrate the coalescent/population process. Putative species clusters are represented as transitions from blue-colored to red-colored branches or as terminal blue-colored branches and are highlighted by numbered circles (1–7). Strains with type status are indicated in bold font. The scale bar represents the expected number of nucleotide changes per site.
